# Identification of Novel Antibacterials Using Machine Learning Techniques

**DOI:** 10.3389/fphar.2019.00913

**Published:** 2019-08-27

**Authors:** Yan A. Ivanenkov, Alex Zhavoronkov, Renat S. Yamidanov, Ilya A. Osterman, Petr V. Sergiev, Vladimir A. Aladinskiy, Anastasia V. Aladinskaya, Victor A. Terentiev, Mark S. Veselov, Andrey A. Ayginin, Victor G. Kartsev, Dmitry A. Skvortsov, Alexey V. Chemeris, Alexey Kh. Baimiev, Alina A. Sofronova, Alexander S. Malyshev, Gleb I. Filkov, Dmitry S. Bezrukov, Bogdan A. Zagribelnyy, Eugene Lane, Maria M. Puchinina, Olga A. Dontsova

**Affiliations:** ^1^Institute of Biochemistry and Genetics Russian Academy of Science (IBG RAS) Ufa Scientific Centre, Ufa, Russia; ^2^Moscow Institute of Physics and Technology (State University), Dolgoprudny, Russia; ^3^Department of Chemistry, Lomonosov Moscow State University, Moscow, Russia; ^4^Insilico Medicine, Inc. Johns Hopkins University, Rockville, MD, United States; ^5^Skolkovo Institute of Science and Technology, Skolkovo, Russia; ^6^Department of Chemistry and A.N. Belozersky Institute of Physico-Chemical Biology, Lomonosov Moscow State University, Moscow, Russia; ^7^InterBioScreen ltd, Chernogolovka, Russia; ^8^Faculty of Biology and Biotechnologies, Higher School of Economics, Moscow, Russia; ^9^Faculty of Bioengineering and Bioinformatics, Lomonosov Moscow State University, Moscow, Russia; ^10^Faculty of Medicine, Lomonosov Moscow State University, Moscow, Russia; ^11^Computer Technologies Lab, ITMO University, St. Petersburg, Russia

**Keywords:** novel antibacterials, machine learning techniques, translation inhibitors, virtual screening, Kohonen-based SOM

## Abstract

Many pharmaceutical companies are avoiding the development of novel antibacterials due to a range of rational reasons and the high risk of failure. However, there is an urgent need for novel antibiotics especially against resistant bacterial strains. Available *in silico* models suffer from many drawbacks and, therefore, are not applicable for scoring novel molecules with high structural diversity by their antibacterial potency. Considering this, the overall aim of this study was to develop an efficient *in silico* model able to find compounds that have plenty of chances to exhibit antibacterial activity. Based on a proprietary screening campaign, we have accumulated a representative dataset of more than 140,000 molecules with antibacterial activity against *Escherichia coli* assessed in the same assay and under the same conditions. This intriguing set has no analogue in the scientific literature. We applied six *in silico* techniques to mine these data. For external validation, we used 5,000 compounds with low similarity towards training samples. The antibacterial activity of the selected molecules against *E. coli* was assessed using a comprehensive biological study. Kohonen-based nonlinear mapping was used for the first time and provided the best predictive power (av. 75.5%). Several compounds showed an outstanding antibacterial potency and were identified as translation machinery inhibitors* in vitro* and *in vivo*. For the best compounds, MIC and CC_50_ values were determined to allow us to estimate a selectivity index (SI). Many active compounds have a robust IP position.

## Introduction

To the current date, a huge number of diverse small-molecule compounds have been reported as having antibacterial activity against different bacterial strains (Kohanski et al., 2010; Mohr, 2016; Naeem et al., 2016; Kaczor et al., 2017). However, almost all of them were discovered more than a half-century ago, and they are of natural origin, for example, penicillins (Fleming, 2001), cephalosporins (Brotzu, 1948), tetracyclines (Bryer et al., 1948), aminoglycosides (Schatz et al., 1944), and macrolides (McGuire et al., 1952). Some trivial structural modifications were introduced into their structure to improve pharmacokinetic features, reduce off-target side effects, and overcome bacterial resistance, which resulted in a broader range of next-in-class analogues, which were brought to market as well (Abouelhassan et al., 2019; Guan et al., 2019). On the contrary, fluoroquinolones [FQs, e.g., ciprofloxacin (Bauernfeind and Petermuller, 1983)] and linezolid (Spangler et al., 1996) are classified as synthetic antibiotics bearing a structure suitable for modification, and it is not surprising that more than 40 FQs were launched. For instance, lascufloxacin (Kishii et al., 2017), a broad-spectrum antibacterial drug, by Kyorin, is currently undergoing registration procedure in Japan as an oral formulation, while tedizolid, a linezolid analogue, developed by Merck & Co., was approved in 2014 (USA) against acute bacterial skin and skin structure infection (ABSSSI). According to Thomson Integrity Database, more than 4,000 molecules have been claimed as antibacterials during the past 5 years, including the most recent nontrivial 2-oxo-1,3-oxazolidines (2017 US 463908) by Johns Hopkins University, 1*H*-imidazo[4,5-*c*]quinolines by Pfizer (2018 US 629152), and 2-oxo-1,2-dihydrospiro-indoles by Shaanxi University of Science Technology (2018 CN 10285257). Twenty new antibacterial chemotypes have been discussed in the *Journal of Medicinal Chemistry* for the last 2 years (see *Supporting Information*). Many pharmaceutical companies, including big pharma alliances, have recently focused on antibacterial vaccines in their pre-clinical and clinical pipelines, for instance, VLA-1701 (Phase II) (Clinialtrialsgov, NIH, 2018c), ETEC (Phase I) (Clinicaltrialsgov, NIH, 2019b), GC-3107 (Phase I) (Clinicaltrialsgov, NIH, 2017a), PF-06842433 (Phase II) (Clinicaltrialsgov, NIH, 2018a), and PF-06886992 (Phase I), Vi-TCV (Phase III) (Clinicaltrialsgov, NIH, 2018b), rhGM-CSF (Phase II/III) (Clinicaltrialsgov, NIH, 2019d), and LEP-F1/GLA-SE (Phase I) (Clinicaltrialsgov, NIH, 2019c). Several small-molecule antibacterial compounds are currently evaluated in different clinical trials, including *N*-thiadiazolo-substituted piperidine (DS-2969; Phase I, Daiichi Sankyo), two boron-containing molecules [(GSK-070 (Clinicaltrialsgov, NIH, 2019a) and VNRX-5133 (Clinicaltrialsgov, NIH, 2017b); Phase I, GSK, and Phase I, VenatoRx Pharmaceuticals, respectively], benzimidazole-substituted 2*H*-chromen (tegoprazan; registered in 2018 for the treatment of gastroesophageal reflux disease in Korea, RaQualia), novel monobactam (BOS-228; Phase II, Novartis), 2-oxo-3,4-dihydro-1,8-naphthyridine (afabicin bis; Phase II, GSK), substituted 3-phenyl-1*H*-pyrrol-olorofim (Phase II, F2G Ltd.), original 1,6-diazabicyclo[3.2.1]octane-2-carboxamide (nacubactam, a β-lactamase inhibitor; Phase I, Roche), and 1*H*-pyrrolo[3,2-*b*]pyridine (TBA-7371; against tuberculosis, Phase I, AstraZeneca). At first glance, there are no principal barriers in this field; however, this speculative conclusion is rather illusory. *De facto*, biological evaluation of many molecules was discontinued due to the lack of efficiency and resistance barriers. The rate of failure outcomes within this sector is close to that observed in anticancer indication. Anyhow, a relatively high risk of failure makes this area much less attractive for the drug design and development in contrast to other easy-to-use therapeutic niches. Indeed, in recent years, global pharmaceutical players have shied away from this field and have shifted focus to more lucrative long-term treatments to manage generally chronic conditions (Projan, 2003). Considering the industry’s reluctance to invest and support the development of new small-molecule antibiotics, academia and minor pharmaceutical companies are strategically positioned to play a key role in the initial stages of lead identification and optimization. Therefore, the improvement of primarily hit identification programs can dramatically extend a pool of promising lead candidates. Under these conditions, machine learning techniques can be reasonably regarded as one of the most appropriate and effective tools to perform rational selection of the most attractive compounds and to achieve significant success during initial rounds of HTS, thereby providing many diverse starting points for subsequent optimization and development.

Although many QSAR models for describing and predicting the antibacterial activity of small-molecule compounds have been published to date, they are mostly focused on an individual class of compounds or on a pre-defined scaffold (Morjan et al., 2015; Leemans et al., 2016). As a rule, such models are not applicable for diverse compound libraries at all, because input parameters, for example, molecular descriptors, are mainly selected to properly describe the chemical space around a chemotype studied. There are some examples of generalized *in silico* models for the prediction of antibacterial potency of heterogeneous series of molecules (**Table 1**). Most of them were trained with small- to moderate-sized training sets (Garcia-Domenech and de Julian-Ortiz, 1998; Tomas-Vert et al., 2000; Mishra et al., 2001; Cronin et al., 2002; Aptula et al., 2003; Molina et al., 2004; Murcia-Soler et al., 2004; Cherkasov, 2005; Gonzalez-Diaz et al., 2005; Marrero-Ponce et al., 2005; Yang et al., 2009) collected using three data sources of antibiotics (Glasby, 1978; Negwer, 1987; Maynard, 1996). As a result, they contain activity values determined in different assays and conditions with no information about their effective concentration. However, recently published models have utilized more comprehensive and qualitative databases (Karakoc et al., 2006; Yang et al., 2009; Wang et al., 2014; Masalha et al., 2018). For instance, in 2006, Karakoc and colleagues used a complete small-molecule collection that included 4,346 compounds bearing “*vecchio*” scaffolds, particularly 520 antibiotics, 562 bacterial metabolites, 958 drugs, 1,220 drug-like compounds, and 1,104 human metabolites (Karakoc et al., 2006). In 2018, Masalha et al. built a predictive model based on 3,500 molecules, but this dataset was collected using different sources that could provide a great bit of false-positive results (Masalha et al., 2018). Although the database contained compounds with high diversity in structure, most of them were well-known chemical entities and natural products (e.g., caffeine and ricinine), representing the active and inactive domains, respectively. In contrast, in this work, we utilized our large proprietary dataset of highly diverse molecules (*vide infra*) with low structural similarity towards the reported antibacterial compounds. This set was improved by antibacterial compounds obtained from Thomson Integrity Database.

**Table 1:** *In silico* models for the development of novel antibacterial compounds.

No.	N_total_	N_antibiotics_	Number of variables	Technique^a)^	Overall accuracy^b)^ (%)	Ref.
**1**	111	60	7	LDA	93.8/91.5^**^	(Garcia-Domenech and de Julian-Ortiz, 1998)
				ANN	89.0/97.9^**^	
**2**	664	249	62	ANN	94.8^**^	(Tomas-Vert et al., 2000)
**3**	59	24	17	LDA	85.0/84.0^***^	(Mishra et al., 2001)
**4**	661	249	6	LDA	92.6/93.6^*^	(Cronin et al., 2002)
				BLR	94.7/94.3^*^	
**5**	664	249	3	LDA	90.1^**^	(Aptula et al., 2003)
				BLR	92.1^**^	
**6**	351	213	7	LDA	91.0/89.0^***^	(Molina et al., 2004)
**7**	433	217	6	LDA	85.7/87.5^**^	(Murcia-Soler et al., 2004)
			62	ANN	98.7/91.4^**^	
**8**	667	363	7	LDA	92.9/94.0^**^	(Gonzalez-Diaz et al., 2005)
**9**	657	249	34	ANN	92.9^**^/100.0^***^	(Cherkasov, 2005)
**10**	2,030	1,006	8	LDA^c)^	90.4/89.3^**^/93.1^***^	(Marrero-Ponce et al., 2005)
**11**	4,346	520	62	kNN	95.0/95.0^*^/84.4^***^	(Karakoc et al., 2006)
**12**	611	230	36	SVC	100.0^*^/100.0^**^/98.1^***^	(Yang et al., 2009)
				kNN	97.7^**^/96.1^***^	
				DT	98.6^*^/92.3^**^/91.0^***^	
**13**	7,517	2,066	21	kNN^c)^	99.2^*^/81.8^**^/78.3^***^	(Wang et al., 2014)
**14**	2,230	1,051	3	LDA^c)^	85.6/87.2^**^/86.2^***^	(Castillo-Garit et al., 2015)
**15**	3,500	628	4	ISE	94.6/72.0^***^	(Masalha et al., 2018)

^a)^LDA, linear discriminant analysis; ANN, artificial neural network; BLR, binary logistic regression; kNN, k-nearest neighbors; MLR, multiple linear regression; SVC, support vector classification; DT, decision tree; ISE, iterative stochastic elimination. ^b)^*Cross-validation; **internal test set; ***external test set. ^c)^Models that demonstrated the highest quality with external test set

Furthermore, the predictive power of many published models was not verified by cross-validation or by using an external validation set of fairly diverse compounds (Garcia-Domenech and de Julian-Ortiz, 1998; Tomas-Vert et al., 2000; Aptula et al., 2003; Murcia-Soler et al., 2004; Gonzalez-Diaz et al., 2005). Nevertheless, only a small part of these models was employed in a routine virtual screening practice (Marrero-Ponce et al., 2005; Wang et al., 2014; Castillo-Garit et al., 2015; Masalha et al., 2018) and resulted in the discovery of novel hit compounds with a remarkable antibacterial activity (Gonzalez-Diaz et al., 2005; Wang et al., 2014; Masalha et al., 2018). In 2015, Castillo-Garit and co-workers performed a ligand-based virtual screening study of 116 molecules with reported antibacterial activity using the developed QSAR model (Castillo-Garit et al., 2015). The model demonstrated good predictive ability in differentiation between active and inactive molecules. In 2014, an *in silico* study was carried out by Wang et al. using Guangdong Small Molecule Tangible Library (7,500 compounds) to search for new anti-MRSA agents and led to the identification of 56 primarily hits (Wang et al., 2014). Among them, 12 compounds were not reported previously as anti-MRSA agents and exhibited good activity against three MRSA strains. However, for the best compounds, only MIC values against bacterial cell lines were measured with no information about, for example, cytotoxicity towards eukaryotic cells. Therefore, it is hard to assess the SI of these molecules and further perspectives. In contrast, in this study, CC_50_ values against the selected eukaryotic cell lines were determined to estimate this index for the most promising compounds.

For a long time, linear discriminant analysis (LDA) (Garcia-Domenech and de Julian-Ortiz, 1998; Mishra et al., 2001; Cronin et al., 2002; Aptula et al., 2003; Molina et al., 2004; Murcia-Soler et al., 2004; Gonzalez-Diaz et al., 2005; Marrero-Ponce et al., 2005; Karakoc et al., 2006; Castillo-Garit et al., 2015) and ANN (Garcia-Domenech and de Julian-Ortiz, 1998; Tomas-Vert et al., 2000; Murcia-Soler et al., 2004; Cherkasov, 2005; Karakoc et al., 2006) were the most popular machine learning methods that were used for prediction of antibacterial activity. On the contrary, few studies successfully implemented other *in silico* techniques, for example, binary logistic regression (BLR) (Cronin et al., 2002; Aptula et al., 2003), SVM (Yang et al., 2009; Wang et al., 2014), kNN (Karakoc et al., 2006; Yang et al., 2009; Wang et al., 2014), and decision tree (DT) (Yang et al., 2009). Therefore, herein, we placed particular focus on powerful and high-performance machine learning techniques that were not applied for antibacterials before, including Kohonen-based SOMs.

## Materials and Methods

### Biological Evaluation

#### High-Throughput Screening

Primary antibacterial activity of small-molecule compounds was assessed using our unique HTS platform described previously (Osterman et al., 2016). This approach allows us to estimate the mechanism of action of hit molecules based on the specific double-reporter system. Briefly, the red fluorescent protein gene rfp was placed under the control of a sulA promoter that was induced by SOS response. The gene of the fluorescent protein, katushka2S, was inserted downstream of the tryptophan attenuator. Two tryptophan codons were replaced by alanine codons, with simultaneous replacement of the complementary part of the attenuator to prevent the formation of a secondary structure that influences transcription termination. Thereby, the expression of katushka2S is observed only upon exposure to ribosome-stalling compounds. *E. coli* strains BW25113 or JW5503 were transfected with the designed plasmid called pDualrep2. As a result, it was possible to differentiate between three mechanisms of antibacterial action in “one-pot” format: DNA damage (expression of rfp), translation inhibition (expression of katushka2S), and others (inhibition of bacterial growth without expression of any reporter gene). The described assay was successfully validated using well-known antibacterial molecules and antibiotics (**Supplementary Figure 1**). Molecules were purchased from vendor collections and dissolved in DMSO at a concentration of 17 mg/ml (for the first round of HTS). Then, solutions of the compounds were spotted on agar plates with the reporter strain by a 96-channel pipetting head of a Janus liquid handling station (PerkinElmer) in a volume of 2 μl of each sample. Erythromycin (ERY, 1 μl) and levofloxacin (LVX, 1 μl) were added in each plate as control samples. After 16 h of incubation at 37°C, the Petri plates were scanned by a ChemiDoc system (Bio-Rad). Antibacterial activity was preliminary estimated by a thorough visual analysis, measurement of growth inhibition zone and MIC values: 0–4 mm (“−”), 4–7 mm (“+/−”), 7–11 mm (“+”), 11–16 mm (“++”; 25 µg/ml < MIC < 200 µg/ml), 16–20 mm (“+++”; 6.25 < MIC < 25), and 20–25 mm (“++++”; MIC < 6.25). Compounds with an insignificant growth inhibition area (“−,” “+/−,” and “+”; MIC > 200 µg/ml) were defined as inactive because of a relatively high concentration of compounds was used during this step. Molecules that caused strong inhibition of bacterial growth (“++,” “+++,” and “++++”) were classified as active.

#### *In Vitro* Translation Inhibition

##### ^14^C-Test

*E. coli* ΔtolC strain was used to assess translation inhibition *in vivo*. Bacterial cells were cultivated in M9 medium to OD600 0.3–0.5. Then, the tested molecule was added at a concentration of 10 times higher than the determined MIC value to the 200 µl of the cells. After 2-min incubation, 1 µl of ^14^C-labeled valine (256 mCi/mmol) was added to the sample. Cells were incubated further for 2 min at 37°C. After incubation was completed, the sample was centrifuged, culture medium was separated, and lysis was performed with 20 µl HU buffer. The resulting mixture (5–10 µl) was subjected to polyacrylamide gel electrophoresis. The 10% SDS–PAGE gel was run for 60 min at 120 V and stained with Coomassie Brilliant Blue dye. The detection of ^14^C-labeled valine was carried out after 48 h by means of Typhoon GE Phosphorimager.

##### In Vitro Luciferase Assay

*In vitro* transcribed firefly luciferase mRNA was translated in a cell-free system based on S30 cellular extract from *E. coli*. The samples were tested at a final concentration of 100 times lower than that used in the cell-based assay (*vide supra*). To investigate the effect of the selected molecules on the prokaryotic ribosome, a mixture of isolated ribosomes with a compound was kept at 37°C for 5 min without mRNA. Then, mRNA (200 ng) was added to the reaction mixture, and translation was initiated in a 10-ml reaction volume at 37°C for 30 min (Osterman et al., 2017). The translation of mRNA encoding luciferase was evaluated by measurement of enzyme activity using 0.1 mM d-luciferin and a spectrophotometer (PerkinElmer). Two control samples were used: negative (1% DMSO as an indicator that no translation inhibition occurred) and positive (ERY at a final concentration of 0.01 mg/ml as a translation inhibitor). All the measured values were normalized using the positive control baseline and expressed as a percentage.

#### MTT Test

Cytotoxicity was assessed using the MTT (3-(4,5-dimethylthiazol-2-yl)2,5-diphenyl tetrazolium bromide) assay following the standard protocol with some modifications. Four thousand cells per well for VA13 cell line and 2,500 cells per well for MCF7, HEK293T, and A549 cell lines were plated out in 135 μl of DMEM/F12 media in a 96-well plate and incubated at 37°C, 5% CO_2_ for 18 h before treatment. Then, the tested compound (15 μl, media/DMSO solution, the final DMSO concentration in the media was 0.5% or less) was added, and the cell samples were incubated for 72 h. The tested molecule in final concentrations of 50 nM–100 μM (eight dilutions), in triplicate, was applied. Doxorubicin (2 nM–6 μM) was used as a positive control. At the end of the incubation, MTT was added into the media (up to 0.5 mg/ml), and cells were incubated for 2 h followed by removal of the media and addition of DMSO (100 μl). The amount of MTT reduced by cells to its blue formazan derivative was measured spectrophotometrically at 565 nM using a plate reader and normalized to the values for cells treated with the media/DMSO only. CC_50_ value was calculated with “GraphPad Prism 5” software (GraphPad Software, Inc., San Diego, CA). Cytotoxicity of some compounds was also assessed by an independent biological team. Compounds were tested at a single concentration of 10 μM, and the survival rate was obtained.

#### Minimum Inhibitory Concentration

MICs in LB and M9 medium were determined using broth microdilution assay (Wiegand et al., 2008). The cell concentration was adjusted to approximately 5 × 10^5^ cells/ml. The tested compound was serially diluted twofold in a 96-well microplate (100 μl per well). Microplates were covered and incubated at 37°C with shaking. The OD600 of each well was measured, and the lowest concentration of the tested compound that resulted in no growth after 16–20 h was assigned to MIC value.

### Reference Database and Pre-Processing

The crude reference database for *in silico* modeling contained a total of 145,000 small-molecule compounds. Most of them (132,641 molecules) were outputted from our HTS campaign: 1,786 active and 130,855 inactive compounds (a hit rate for a random HTS was typical, 1.35). It should be especially noted that these compounds were highly dissimilar in structure to known antibiotics and antibacterial compounds because the prime goal of our previous work was to identify novel antibacterial scaffolds. The database was improved by the known antibacterial compounds obtained from Thomson Reuters Integrity database in order to increase the number of active samples and to cover the entire chemical space. In total, 12,347 molecules were added. Duplicate structures were removed using ChemoSoft software. Antibacterial molecules frequently contain specific substructures that are rather unusual in other therapeutic indications. Therefore, in this case, several medicinal chemistry filters (MCFs) cannot be properly applied to exclude undesired molecules. Thus, only “absolutely” nondrug-like molecules (e.g., metal-, silicon- and phospho-organic compounds, extensive linear aliphatic moieties, and sugars) as well as compounds containing highly toxic or unstable/reactive groups (e.g., strained heterocycles, isatines, hydroxamic acids, acylated imidazoles, and disulfides) were eliminated. Charged items were redrawn and presented in their neutral form, salt parts were deleted, and errors in structures were manually corrected. Then, the database was clustered using ChemoSoft software with the following parameters: Tanimoto similarity threshold ≥ 0.5 and the number of structures per cluster ≥ 5. In order to increase the common diversity of the dataset and to decrease the number of overrepresented structures, only 30 members with upper diversity coefficients per each cluster, as well as singletons, were retained. As a result, the final database contained 74,567 compounds (8,724 active and 65,843 inactive). The main parameters of the training dataset are listed in **Table 2**.

**Table 2:** Key features of the training dataset.

Number of compounds	Active	Inactive	Diversity*	Unique heterocycles			Clusters**	Av. cluster size	Singletons
				All	Active	Inactive			
74,567	8,724	65,843	0.86	3,961	1,146	3,370	2,021	15	22,521

*Reverse Tanimoto metrics; **min. cluster size, 5; max. cluster size, 30; similarity threshold, 0.5.

### Molecular Descriptors

Molecular descriptors (total of 1,749) were calculated for the whole training dataset using Dragon, ChemoSoft, MOE, and SmartMining (Pletnev et al., 2009) software tools. The number of descriptors was reduced to 1,243 by the omission of constant, near-constant, and highly correlated (*R* > 0.9) descriptors. *A priori*, we excluded a series of ordinary descriptors (e.g., number of exact and query substructures as well as fingerprints) to overcome overfitting, like in the case of β-lactams, fluoroquinolones, linezolid analogues, and other structure-biased antibacterials, and to objectively describe the input chemical space by a comprehensive set of key physicochemical molecular properties related with antibacterial potency. Then, the *t*-statistic was calculated for the remaining descriptors. Those with the best *t*-values were selected accounting their theoretical impact on the studied phenomenon (**Supplementary Table 1**) followed by PCA analysis (*Supporting Information*). As a result, we selected 40 molecular descriptors to perform the learning procedure. These include topological and electrotopological descriptors, lipophilicity and polarity indexes, the number of potential H-bond donors and H-bond acceptors, number of free-rotatable bonds and drug-likeness violation, atomic contribution to molar refractivity and autocorrelation, partial van der Waals surface area, and symmetry indexes (**Supplementary Table 2**).

### *In Silico* Modeling

#### SOM

SOM (Kohonen, 1990) is one of the most powerful machine learning techniques that map multidimensional data onto lower-dimensional subspaces where geometric relationships between points indicate their similarity. Considering this fact, the output may be easily interpreted. However, this method requires a large amount of input data in order to achieve an appropriate predictive power. Kohonen-based SOM was constructed in SmartMining Software. The map size was 30 × 30 nodes (2D representation, of total 900 nodes, random distribution threshold was 82 samples per neuron), tetragonal cell, learning epochs: 2,000, initial learning rate: 0.3 (*linear decay*), initial learning radius: 15 (*linear decay*), activation function: Gaussian, winning neuron was determined using the standard Euclidean metrics, initial weight coefficients: random distribution, input vector: 40 descriptors (*not normalized*). Three independent randomizations were used to assess the reproducibility and stability of the model. After the unsupervised training process was completed, neurons were prioritized based on the following privileged factor (PF): N*_i_* ^ab^ (%)/N*_i_* ^nab^ (%), where N*_i_* ^ab^ is the percent of antibacterials located in the *i*th neuron and while N*_i_* ^nab^ is the percent of nonantibacterials located in the same neuron and vice versa. PF value greater than 1 was used as a threshold to assign neurons to one of these two classes.

#### kNN

kNN (Zhang, 2016) is one of the simplest machine learning algorithms. However, its predictive performance and low computational costs make it one of the most used machine learning methods. This algorithm is based on feature similarity: the test sample is classified according to the nearest neighbors from the training dataset. However, the simplicity of kNN is associated with its inability to achieve an appropriate classification performance in case of complex data. In order to achieve the best predictive power, the following parameters of the classifier were varied: a number of neighbors (3–9, default 5), weights (“uniform” or “distance”), power parameter for the Minkowski metric (*p* = 1 for Manhattan distance and *p* = 2 for Euclidean distance).

#### Training Dataset Segmentation

Considering that the following *in silico* techniques use a supervised learning procedure, the randomized training datasets were subdivided into three categories in order to correctly estimate their classification accuracy: training set, cross-validation set, and internal test set (Balakin et al., 2004). The cross-validation subset was used to avoid model overfitting during the learning procedure, and the internal testing subset was used for pre-validation of the developed models. The learning settings were varied in order to reach the best classification accuracy. All the algorithms below were realized using scikit-learn library for Python 3.6.

#### GB

Gradient boosting (Friedman, 2001) is one of the most powerful machine learning methods. It is an ensemble technique, in which new models (*decision trees*) are added to correct the errors by the existing models. Models are added sequentially until no further improvements can be made. GB is relatively resistant to an increase in the number of decision trees, so this usually leads to greater performance. Increasing the maximum depth does not always improve the prediction quality and may lead to overfitting and an increase in training time. The learning parameters were varied in order to reach the best classification power. The default values were the following: The number of trees was 100, and maximum tree depth was 3.

#### RF

In contrast to GB, random forest (Breiman, 2001) is based on “fully grown” decision trees that are trained independently using a random sample of data. It should be noted that both GB and RF may be trained without preparation of the input data (scaling or normalization). One of the main advantages of RF compared with GB is the simplicity of model tuning. However, it is less resistant to an increase in the number of basic classifiers that also leads to a dramatic increase in computational costs. The default parameters of the model were the following: the number of trees was 10, and the maximum depth was not limited (building a tree until all leaves were empty or all leaves contained less than two elements).

#### FFN (Feedforward Neural Network)

Artificial neural networks (Sazli, 2006) usually perform slightly better than the classifiers described above. However, overfitting is the main problem during the training procedure. Thus, different regularization techniques, parameters tuning, and accurate feature selection are required to achieve an appropriate classification accuracy. Moreover, FFN training procedure requires intense computational cost than do the other classifiers. One of the main disadvantages of neural networks is their “black box” nature. It is hard to understand how the prediction has been made. The three-layer neural network was constructed as follows: 30 neurons in the input layer, 100–150 neurons in the second layer, 30–80 neurons in the third layer, and 1 neuron in the output layer. The number of learning epochs varied from 1000 to 2000; initial learning rate was 0.1 (linear decay coefficient 0.01); weights were initialized randomly; dropout technique was used to prevent overfitting.

#### SVM

SVM (Cortes and Vapnik, 1995) is a supervised machine learning algorithm that can be used for both classification and regression tasks. In this algorithm, each data item is plotted as a point in *n*-dimensional space with the value of each feature being the value of a particular coordinate. Then, classification is performed by finding the best hyperplane that differentiates two predefined classes. The main advantage of SVM is the possibility of using different kernels. Kernels are functions that transform low-dimensional input space to a higher-dimensional space where the classes can be separated. However, it is usually hard to choose hyperparameters of the SVM for sufficient generalization performance. The following parameters of SVM were used: penalty parameter (1.0 ≤ *C* ≤ 10.0) and kernel (linear, RBF, polynomial, and sigmoid).

### Experimental Validation

All the models described above were used to predict the antibacterial activity of novel molecules (5,000) randomly selected from the available vendor`s collections. These testing samples were selected using a threshold Tanimoto-based similarity value < 0.5 towards the training samples. All the compounds obtained were investigated for their antibacterial potency using the assays listed above. Biological results were then used to assess the prediction power of the models.

## Results

### High-Throughput Screening

We used extensive proprietary data on the antibacterial activity of small-molecule compounds obtained during our HTS campaign. Screening molecules were selected from the stocks based on the following core principles: a) a relatively low structural similarity towards the reported antibacterial compounds and antibiotics, b) maximum diversity in structure per each cluster, c) all the remaining singletons (molecules that were not fitted in any cluster) were included as well, and d) maximal covering of the common chemical space provided by suppliers. In general, we used two collections of commercially available compounds by ChemDiv and IBS. While ChemDiv stock mainly contains organic compounds of synthetic origin, IBS basically focuses on nature-based molecules and their close analogues. To our satisfaction, to the current date, these obstacles have been overcome, and we have recently initiated a follow-up HTS round with EA compounds.

### Reference Database and Pre-Processing

To estimate the quality of covering the whole chemical space by the pool of the selected compounds, we constructed Sammon-based nonlinear map using the descriptors listed above (Sammon, 1969). Prior to mapping, we performed clustering analysis and rejected molecules with high similarity in structure per each cluster. We also applied soft MCFs to the final database for the exclusion of marginal nondrug-like structures. During Sammon mapping, we observed that about 70% of the collections used were normally covered by the remaining molecules. Therefore, we can speculate that they reflect the key features of the collections used more reliably and objectively versus random selection. In other words, we were trying to reach maximal covering and diversity with a minimal number of compounds. The pre-processed database was then used as a training set for *in silico* modeling.

### Molecular Descriptor Feature Selection

**Figure 1:**
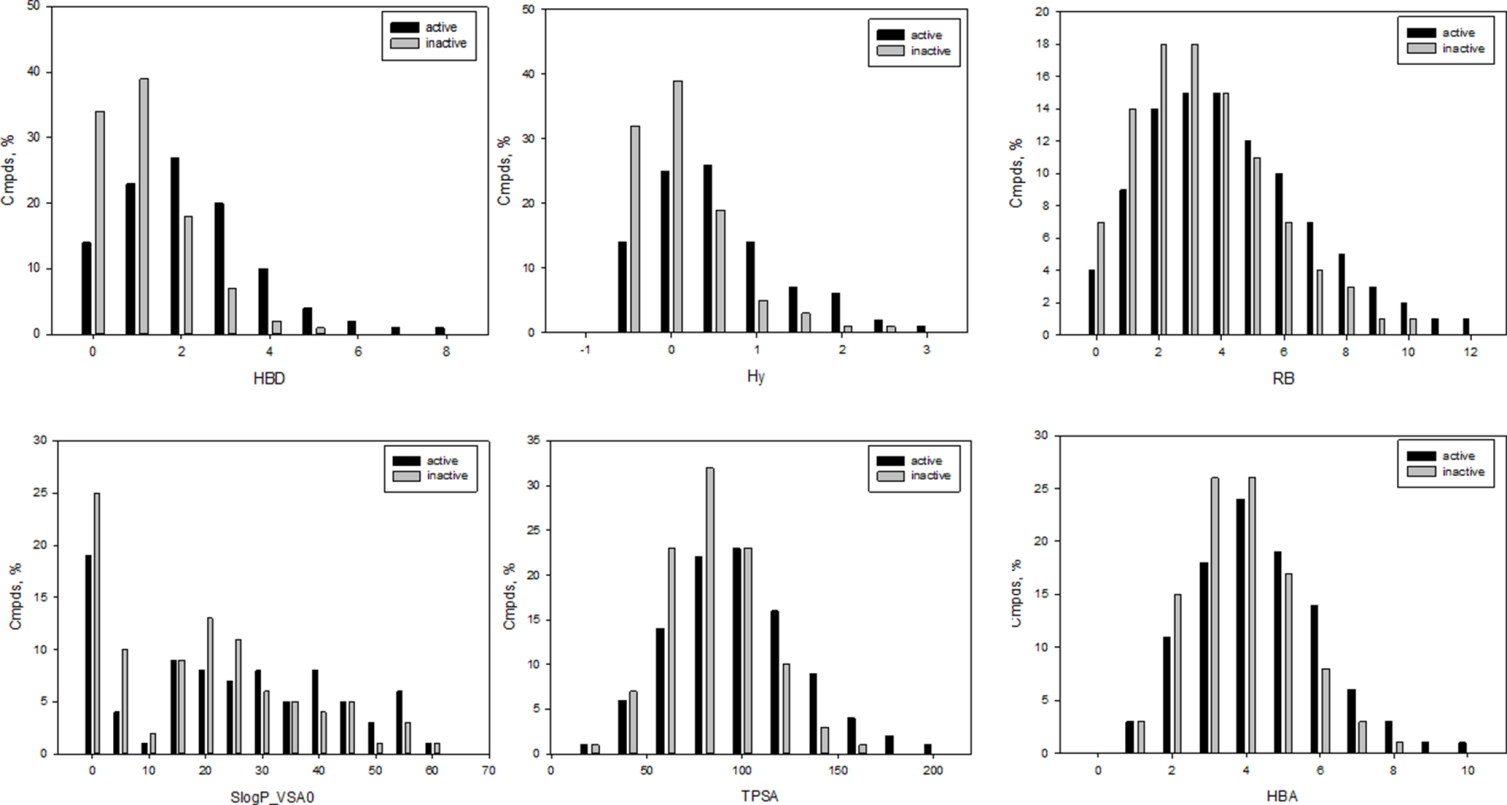
Representative examples of molecular descriptors included in the final set of input variables; HBD, number of potential H-bond donors, Hy, hydrophilic factor, RB, number of free-rotatable bonds; LogP_VSA, reflects hydrophobic and hydrophilic effects; TPSA, polar surface area; HBA, number of potential H-bond acceptors.

It should be especially noted that several of the selected molecular descriptors were described previously as important for statistically significant separation between antibacterial and nonantibacterial compounds (Araya-Cloutier et al., 2018). Distributions for the representative descriptors used for learning herein are depicted in **Figure 1**. As shown in **Figure 1**, Hy (F2 = 0.75) and HBD (F2 = 0.72) were among the best scored variables with *t*-coefficient higher than 40. Of the total, 25 molecular descriptors were classified as core inputs on the basis of *t*-stat analysis (*t* > 30). Several descriptors were less significant providing lower *t*-values, for instance, S(═N–) (*t* = 8.7, F4 = 0.48), GVWAI-80 (*t* = 24, F12 = 0.62), and M1 (*t* = 29, F1 = 0.83); however, in contrast to Hy and HBD, they were disposed in distinct areas of the common PCA plot (*Supporting Information*) and, therefore, contributed well to the exposition of the input chemical space, as well as the classification. Indeed, the exclusion of inputs with low-rate *t*-value led to the reduction of classification accuracy. Moreover, we performed non-parametric Mann–Whitney *U* test to prove the correctness of the description selection. The results of *U* test correlated with *t*-stat values (**Supplementary Table 6**). Based on the performed PCA analysis, 18 molecular descriptors were found to reflect 90% of the entire variability.

### *In Silico* Modeling

Because the dataset was highly imbalanced (eight times more inactive molecules vs. active), the models constructed using the remaining algorithms classified the majority of the samples as inactive, achieving a relatively high overall accuracy. Manipulations with the class weights parameters did not lead to any improvement in the classification ability. As a result, we constantly observed overfitting passages that are highly undesirable in machine learning. In order to balance the numbers of the compounds of both classes, the inactive molecules were clustered (Tanimoto similarity > 0.7), and the singletons were added to the nearest clusters. Each cluster was equally split into four different subsets (**Supplementary Table 3**), which resulted in four independent training sets (15,961 inactive and 7,724 active molecules in each “echelon”). The remaining 2,000 inactive and 1,000 active compounds were merged to form the internal test set. The antibacterial activity of the molecules was predicted using all these models and assigned for each sample based on the consensus score value. RF classifier demonstrated a relatively low classification accuracy. The best results were obtained using 100 classification trees (other parameters were kept as default). The average accuracy with the internal test set was 79.5% (90.2% for inactive and 68.8% for active compounds). GB provided almost the same results (average accuracy was 79.9%). More advanced algorithms (SVM and FFN) performed slightly better than decision tree-based classifiers. It should be noted that data scaling is strongly required to achieve better performance for these techniques. Thus, each descriptor vector was standardized using the scaling tool of scikit-learn library. The best classification results with SVM were obtained using the following parameters: penalty parameter of the error term (*C* = 10.0, kernel = rbf because other kernels, such as sigmoid, polynomial, and linear, demonstrated worse results). The average accuracy of the constructed models was 82.7% (91.5% for inactive and 73.9% for active compounds). FFN was implemented using Keras library for Python 3.6. Three-layer FFN showed the best average classification accuracy 81.3% (90.5% for inactive and 73.1% for active compounds). Other parameters of the neural network were the following: number of training epochs = 1,000; regularization, dropout (0.3 for each layer); activation function, sigmoid; and initial learning rate = 0.01.

**Figure 2:**
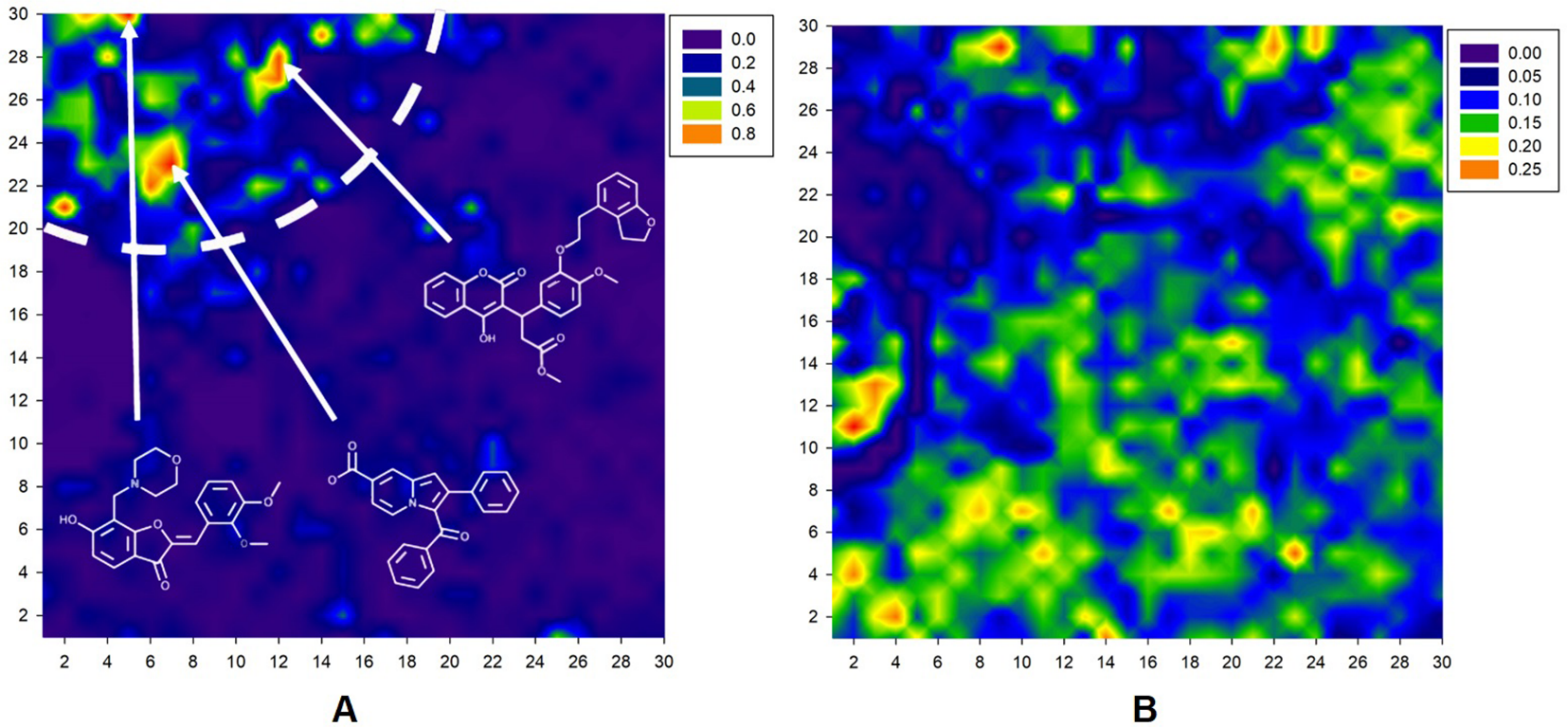
A 30 × 30 2D Kohonen SOM for discrimination between antibacterial **(A)** and nonantibacterial **(B)** compounds within the same map. Color gradient corresponds to the percentage of molecules. Basic contours of the map were smoothed for a convenient visual inspection.

The resulting Kohonen map is presented in **Figure 2**. As shown in **Figure 2A**, antibacterial compounds are located predominantly within a tight area of the whole map distinct from the nodes abundantly populated by nonantibacterial molecules (**Figure 2B**). The arrows in **Figure 2A** denote the location of different antibacterial drug classes in the map. At the final iteration, learning vector quantization error (LVQE) was relatively low, reaching a maximum value of 0.012 (**Supplementary Figure 2**). More than 90% of samples provided LVQE of less than 0.002. Therefore, we can conclude that the constructed model has very good generalization ability and learning outcome. The stability of the model was verified using three independent randomizations. Moreover, the addition of a fuzzy input with stochastic variables did not strongly affect the quality of classification. Upon examination, there were few “dead” neurons within the constructed map. The average classification accuracy was 77.5% and 69.8% without and with a random threshold, respectively.

The best predictive power with the internal test set was obtained using kNN. The most significant parameters that affected the classification accuracy were the number of neighbors (the best value = 3) and the weight function used in prediction (the best one was “distance” that weighted points by the inverse of their distance, so closer neighbors of a query point had a greater influence than neighbors that were further away). Euclidean metric was used for distance calculation. The prediction accuracy was 83.2% (88.7% for inactive and 77.7% for active compounds).

**Figure 3:**
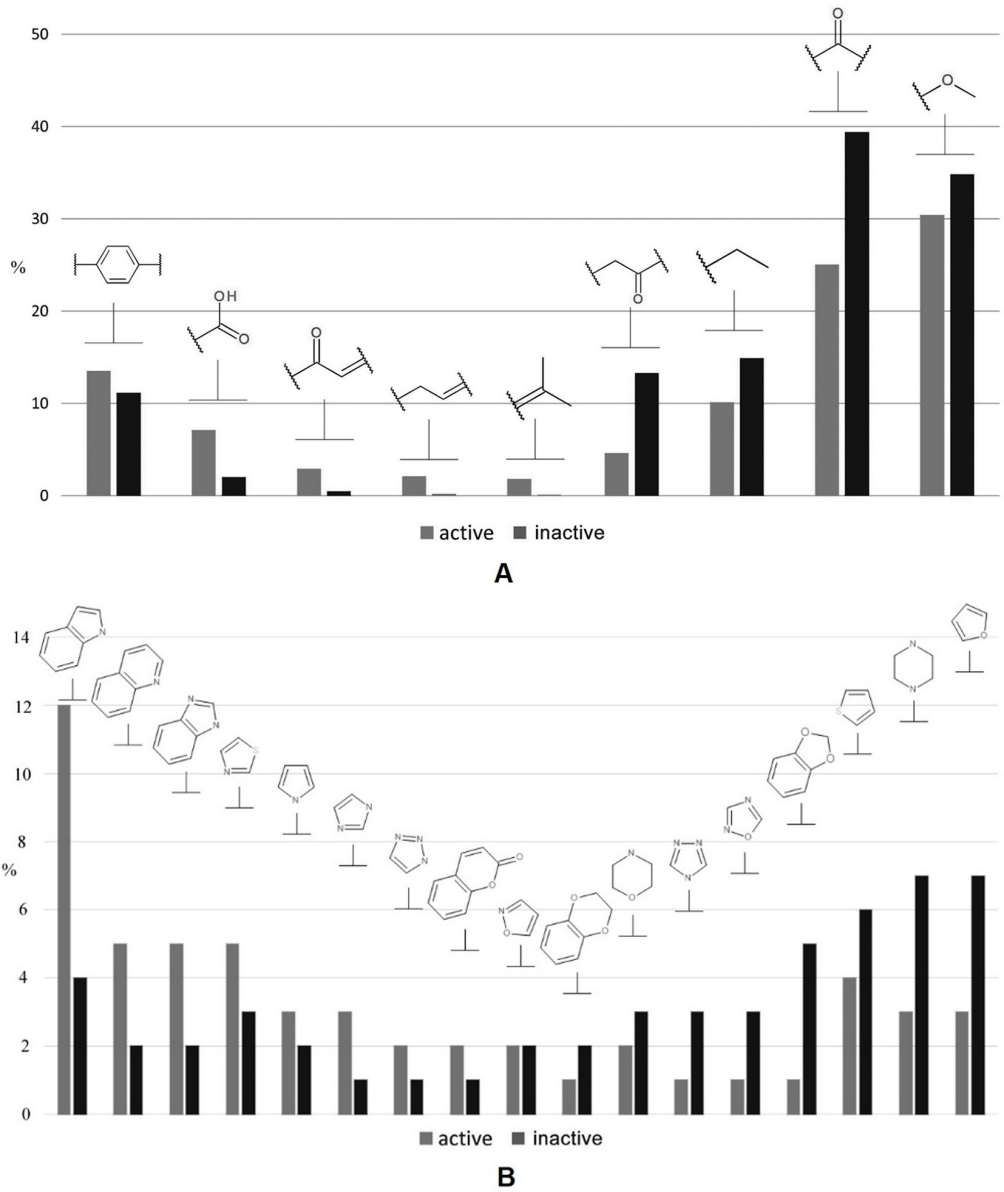
A brief statistical analysis on basic nonheterocyclic **(A)** and heterocyclic **(B)** fragments presented in antibacterial and nonantibacterial compounds.

In addition, we performed a comprehensive statistical analysis of various nonheterocyclic (**Figure 3A**) as well as heterocyclic (**Figure 3B**) fragments presented in both classes. Among the first category, the methoxy (30.5% and 35% for active and inactive compounds, respectively) and carbonyl groups (39% and 25%) are the most represented. Nonantibacterial compounds contain 1.56 times greater number of carbonyl fragments in contrast to antibacterials, while the methoxy group does not provide a statistically significant separation between two classes studied. The accuracy of propanoyl moiety among inactive compounds is 3 times higher than in active samples. Carboxylic, α,β-unsaturated carbonyl, and allyl are the most characteristic moieties for antibacterial compounds: respectively 3.75, 6, and 9 times higher rate than the inactive class. With respect to heterocycles, indole is the most represented (12%) heterocyclic fragment among antibacterials. The rate of indole, imidazole, quinoline, and benzimidazole fragments is greatly biased towards antibacterial compounds, while furan and piperazine (∼7%) are 2.3 times more abundant in nonantibacterials. In addition, 1,3-benzodioxole fragment is privileged for inactive molecules, while isoxazole is equally found in both classes. It should be especially noted that several molecular descriptors included in the final set for performing learning procedure are closely related with the statistical observations below. For instance, the common polarity encoded by S(–OH), S(–O–), S(═N–), S(> N–), HB2, a_acc, O-057/061, PEOE_VSA_FPOS, and TPSA corresponds to methoxy, carbonyl, propanoyl, carboxylic, and α,β-unsaturated carbonyl groups and heterocycles, while Hy and SlogP_VSA0 contribute to lipophilicity, particularly taking into account linear and branched alkyl moieties as well as aromatic fragments. Topology of a structure relates, for example, with M1, SPI, EEig07x, Q′, VEA2, and GATS1m.

### Experimental Validation

To investigate the prediction power of the constructed models, we used an external test set of 5K small-molecule compounds with similarity in structure of less than 0.5 to the whole training set. These molecules were randomly selected and purchased from ChemDiv and IBS collections. Antibacterial activity of the compounds was predicted using the developed models and then evaluated following the biological protocols described above. We did not use a consensus score value per each sample and retained compounds, which were predicted as inactive to estimate the prediction “resolution” of the models towards both classes used. This allowed us to get a valuable feedback on a possible overfitting or bias during the learning procedures. The first round of HTS has resulted in 371 active compounds (hit rate = 7.4%) followed by rescreen procedure performed at lower concentration. Rescreen confirmed moderate-to-high antibacterial activity for 65% of molecules. It should be especially noted that among all the active molecules from the initial HTS (molecules included in the training set) and from the external test set, only a few compounds showed a considerable inhibition activity against *E. coli* ^wt^. Several compounds demonstrated a robust SOS response or inhibition of translation machinery. A few compounds showed both signals but with a relatively low intensity. A brief summary of the performed biological evaluation is presented in **Table 3**. Due to confidentiality reasons, we cannot disclose the structures of the lead compounds. As shown in **Table 3**, among the listed molecules, the highest antibacterial potency was revealed for FQ analogue **7** (MIC = 0.8 µg/ml), 6*H*-thiazolo[4,5-*d*]pyrimidinone **9** (MIC < 0.2 µg/ml), (6-oxo-1*H*-pyrimidin-2-yl)pyrazole **10** (MIC < 0.2 µg/ml), substituted thiadiazoles **11** (MIC = 0.8 µg/ml), hydroxy-pyrazole **12** (MIC = 0.8 µg/ml), and bithiophene **13** (MIC = 1.8 µg/ml). Compounds **1** and **2** strongly inhibited translation at 16 µg/ml and provided good SI. Furthermore, compound **2** showed a comparative antibacterial potency against several mutant strains (*these results will be published shortly*). Two compounds **7** and **8** induced a considerable SOS response, MIC = 0.8 and 20.8 µg/ml, respectively; however, compound **8** has lower SI. Among molecules acting *via* other mechanisms, compound **11** can be attributed to a wide class of sulfanilamide-based inhibitors (PABA analogues) of dihydropteroate synthase. Cytotoxicity against a panel of eukaryotic cells is summarized in the Supporting Information (**Supplementary Table 4**). The IP position of the molecules was assessed using SciFinder and Integrity Databases.

**Table 3:** Representative examples of active compounds that were correctly predicted as antibacterials. The detailed biological results are presented in **Supplementary Figure 3**.

ID	Structure	Activity	ID (from database)	MIC (µg/ml, ΔtolC)	Mechanism of action	In vitro translation	^14^C-test	SI^*^	IP^**^
**LVX**	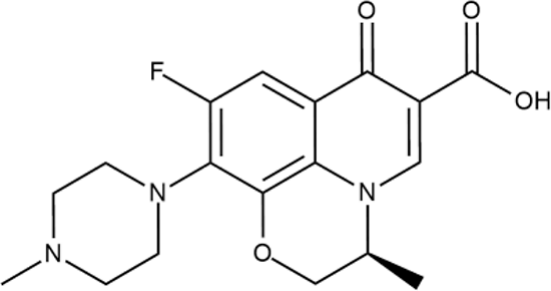	++++	-	0.016 ± 0.009	SOS	−	−	H	-
**ERY**	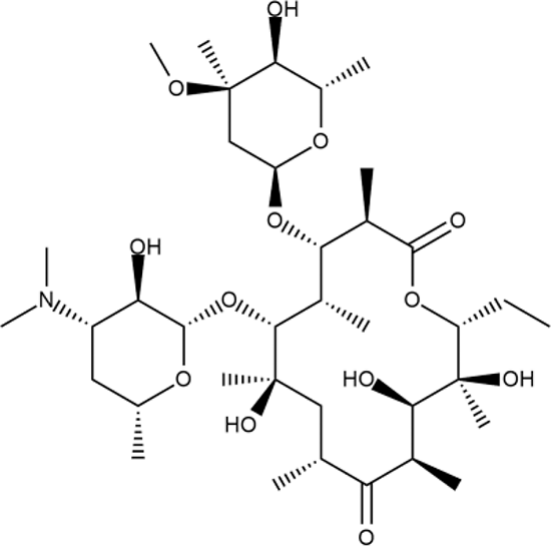	++++	-	2.5 ± 0.5	T	+	+	M	-
**1**	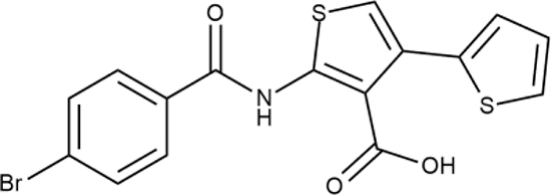	+++	STOCK1S-88700	1.8 ± 0.8	T	+	+	M	M
**2**	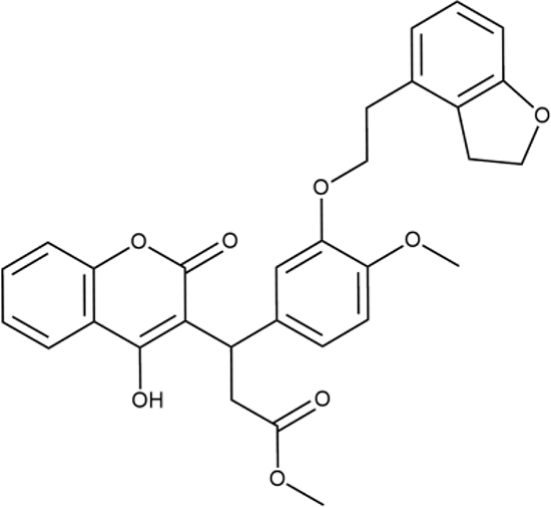	+++	STOCK1N-86948	2 ± 0.4	T	+	±	M	M
**3**	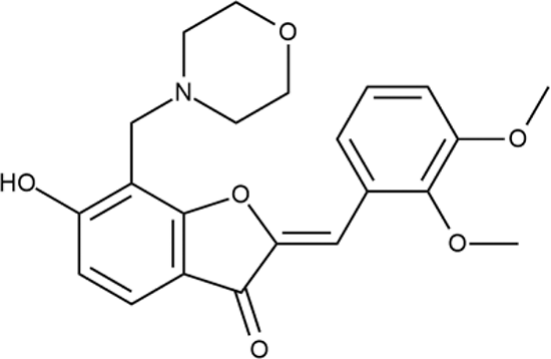	++++	STOCK1N-55723	3.9 ± 1.4	S + T	+	−	L	H
**4**	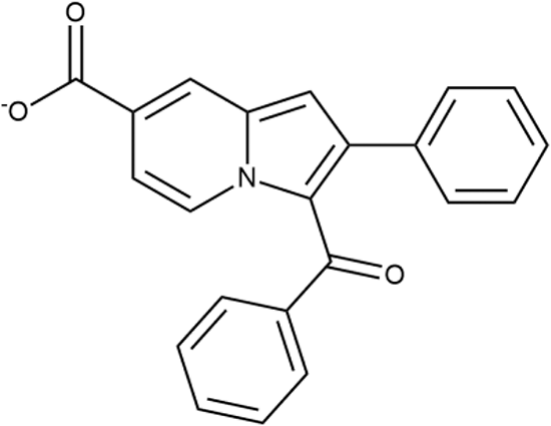	+++	D090-0093	6.25 ± 1.3	T	+	+	H	H
**5**	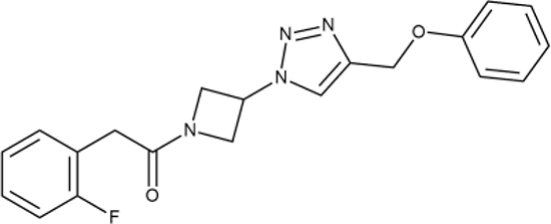	++	P991-0387	12.5 ± 1.9	T	±	−	H	H
**6**	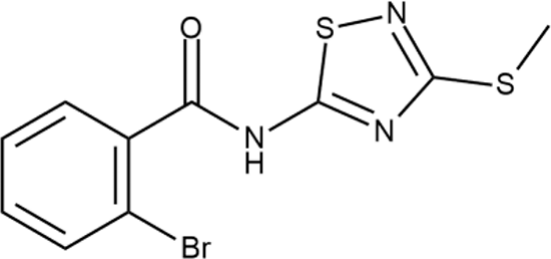	+	F333-0013	42 ± 5	T	+	+	H	M
**7**	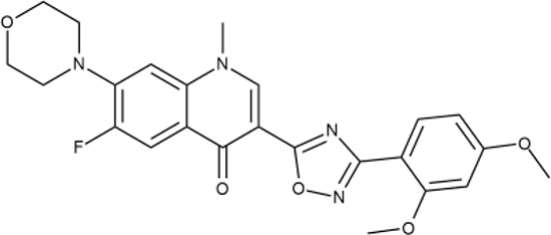	+++	F418-0205	0.8	SOS	−	−	H	M
**8**	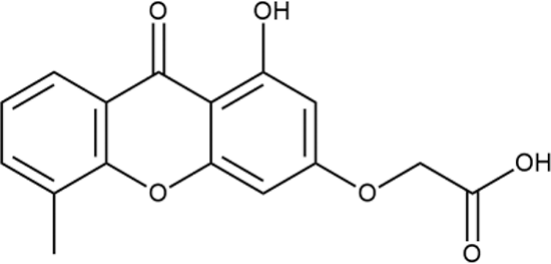	+++	STOCK1N-64226	20.8	SOS	−	−	L	H
**9**	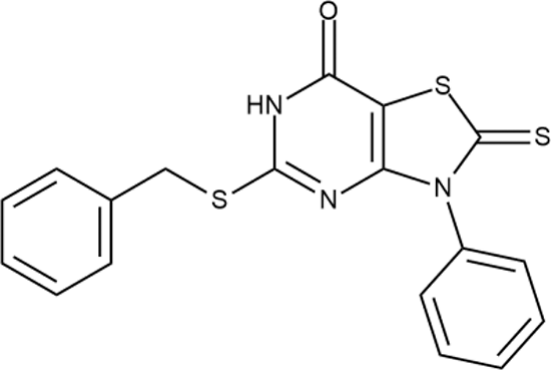	+++	F092-0369	<0.2	O	−	−	H	M
**10**	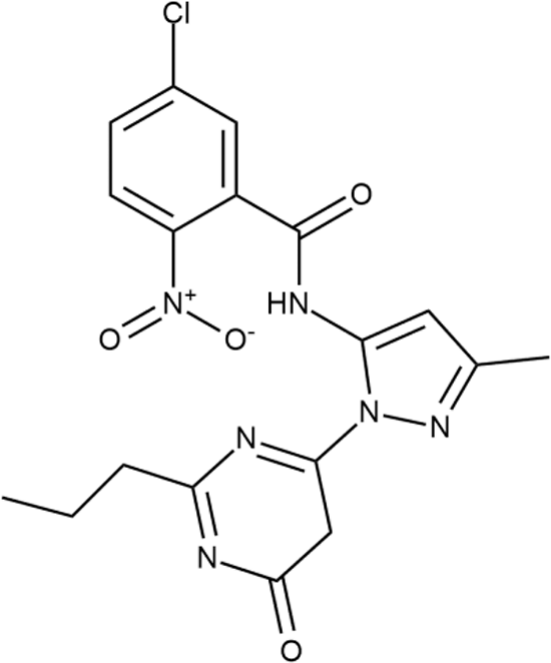	+++	F269-0279	<0.2	O	−	−	H	H
**11**	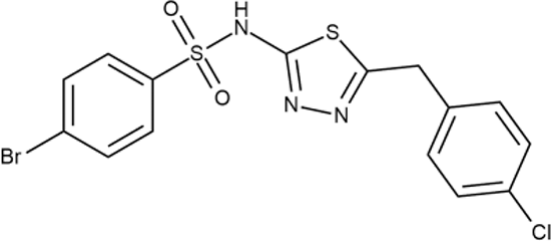	+++	Y030-6952	0.8	O	−	−	H	H
**12**	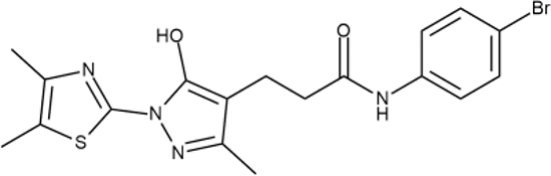	+++	D475-2799	0.8	O	−	−	M	H
**13**	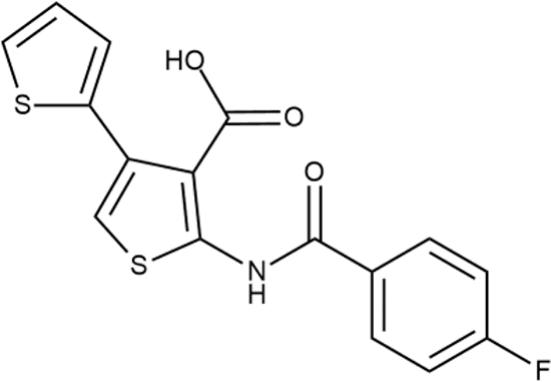	+++	STOCK2S-91453	1.8 ± 0.8	T	+	+	M	M

*SI, selectivity index = CC *_50_* (µg/ml or %)/MIC (µg/ml); H, high, SI > 100; M, moderate, 20 < SI < 100; L, low, SI < 20; T, translation inhibition; SOS, SOS response, O, other mechanism of action; **IP, intellectual property; L (low), match antibacterial Markush structure; M (moderate), match non-antibacterial Markush structure (but not listed among examples); H (high), clear IP status.

In order to make the study more informative, two hit compounds, **11** (4-bromo-*N*-{5-[(4-chlorophenyl)methyl]-1,3,4-thiadiazol-2-yl}benzene-1-sulfonamide) and **13** (5′-(4-fluorobenzamido)-[2,3′-bithiophene]-4′-carboxylic acid), were studied on antimicrobial activity against selected archival strains: *E. coli* ATCC 25922, *Klebsiella pneumoniae* 181210171-2, *Pseudomonas aeruginosa* ATCC 27853, *Staphylococcus aureus* ATCC USA 206, and *Candida albicans* 181210169-1 (**Table 4**). These substances exhibited modest activity against gram-negative bacteria *K. pneumoniae*. A similar pattern was observed on *C. albicans* multi-resistant strain. Compound **13** only slightly inhibited the growth of *E. coli*, while compound **11** did not demonstrate activity against this strain. No activity against *P. aeruginosa* was detected. The outstanding antimicrobial activity of compounds **11** and **13** was revealed in the tests on gram-positive cocci of *S. aureus*. The growth inhibition zone during the tests exceeded 20 mm.

**Table 4:** Antibacterial activity of compounds **11** (4-bromo-*N*-{5-[(4-chlorophenyl)methyl]-1,3,4-thiadiazol-2-yl}benzene-1-sulfonamide) and **13** (5′-(4-fluorobenzamido)-[2,3′-bithiophene]-4′-carboxylic acid) against selected archival strains.

Species	Strain ID	Source	Activity	
			Compound 11	Compound 13
*Escherichia coli*	ATCC 25922	ATCC*	−	±
*Klebsiella pneumoniae*	181210171-2	Clinic of the Bashkir State Medical University	±	+
*Pseudomonas aeruginosa*	ATCC 27853	ATCC	−	−
*Staphylococcus aureus*	ATCC USA 206	Clinic of the Bashkir State Medical University	++++	++++
*Candida albicans*	181210169-1	ATCC	+	±

*ATCC, American Type Culture Collection.

## Discussion

In contrast to numerous focused QSAR studies with recruiting small- or medium-sized reference databases of small-molecule compounds having a common scaffold or high similarity in structure, generalized *in silico* approaches for solving nontrivial classification problems cannot be adequately applied without a representative and comprehensive training dataset harmonically populated with a sufficient number of appropriate samples. These samples should almost ideally cover a whole input space providing maximum diversity. Any pattern within this space should contain bits of information important for learning procedure to achieve theoretically valid and interpreted results. This issue becomes one of the most crucial limitations, especially in the area of computer-aided modeling and the prediction of antibacterial activity. Considering a long-term period of a permanent stagnation in the field of development of novel antibacterials, a revolutionary breakthrough can be achieved using more powerful *in silico* approaches with an improved mining ability and prediction quality. These models are likely to achieve success in searching for principally new antibacterial chemotypes and to possibly overcome an overwhelming bacterial resistance.

In our work, the best results of *in silico*modeling were obtained using more advanced machine learning methods: Kohonen SOM, FFN, and SVM (**Table 5**). As it was expected, the predictive power of kNN was insufficient for this task. RF and GB performed slightly better. However, they failed to predict active molecules correctly in consequence of their tendency to overfit. Thus, Kohonen SOM, FFN, and SVM can be used for the *in silico* assessment of antibacterial activity. However, as it was discussed earlier, FFN and SVM did not perform well on the highly imbalanced dataset, and it was decided to split it in a different manner. Despite the achieved predictive power, the manipulations with input data may result in loss of information and require additional time-consuming data preparation steps. Thereby, Kohonen SOM is likely to be a more preferable and effective tool due to the following reasons: a) the resulting maps are very convenient for visual inspection of patterns occupied by compounds from different classes and for the distribution of molecular descriptor values within the map; b) in contrast to other machine learning techniques described above, overfitting was not observed for SOM using the training set of 73,000 samples, thereby providing more appropriate and reliable generalization; and c) in addition to a range of implemented settings, there are some advanced modifications of the algorithm (e.g., neural gas, convex combination, Grossberg-layer hybrid SOM, and Duane-Desieno method), which can be effectively used for improving the learning procedure and discrimination ability.

**Table 5:** Overall performance o*f in silico* modeling.

Model	Classification accuracy (%)*									Prediction accuracy active/inactive (%)^**^
Set	Training			Cross-validation			Internal test			
Subset	Active	Inactive	Average	Active	Inactive	Average	Active	Inactive	Average	
**SOM**	75	80	77.5	–	–	–	–	–	–	73/78
**FFN**	83.2	93.4	88.3	74.2	90.5	82.4	73.1	90.5	81.3	72/77
**RF**	100	100	100	70.7	92.7	81.7	68.8	90.2	79.5	69/80
**GB**	79.2	95.7	87.5	68.5	91.1	79.3	68	87	77.5	68/81
**SVM**	84.5	97.6	91.0	73.5	91.7	82.6	73.9	91.5	82.7	73/78
**kNN**	100	100	100	77.9	87.9	82.9	77.7	88.7	83.2	63/76

*Values for the best randomization; ** external test set.

Experimental* in vitro* validation of developed models during first round of HTS and following rescreen procedure demonstrated relatively high hit rate considering the random compound selection for the external test set. Most of the most active molecules (**Table 3**) have moderate-to-high selectivity index and IP status. Two compounds (**11** and **13**) demonstrated satisfactory activities against several archival strains of microorganisms.

In summary, for the first time, we used a very large database of our proprietary HTS results to construct a highly discriminative and robust *in silico* model able to score molecules by their antibacterial potency against *E. coli*. The main focus was placed on compounds with low similarity in structure to the reported antibacterials, as well as maximum diversity. Forty of the most reliable molecular descriptors were rationally selected from a whole pool of more than 1,700 calculated features. The final set of descriptors reflects several key aspects in privileged structures presented in antibacterial or nonantibacterial compounds and significant patterns hidden in the input chemical space. Cumulative *in silico* modeling with recruiting several machine learning techniques showed that, using this dataset, two polar categories of compounds could be successfully separated providing good classification index. These models were then used to predict the antibacterial and nonantibacterial potency of novel compounds, which were not included in the parent database. Molecules from this external pool bore a relatively low structural similarity towards the training samples. Subsequent biological evaluation confirmed an attractive predictive power of the developed models. In particular, Kohonen-based SOM has not been used previously for solving the title task and demonstrated very promising results. Although we cannot disclose the structures of the best hits because of confidentiality reasons, the presented active molecules showed good antibacterial activity and can be reasonably regarded as convenient starting points for further optimization and morphing. Some compounds effectively inhibited translation in prokaryotes and showed no or weak cytotoxicity against a small panel of eukaryotic cell lines, thereby providing a benefit SI. With the use of the specific professional databases, the IP position of the molecules was preliminary assessed. The developed model can be effectively applied especially in academic organizations or small- to moderate-sized pharmaceutical companies to perform the rational selection of compounds for the primary HTS campaigns, thereby reducing the total costs of the entire R&D process.

## Data Availability

The datasets generated for this study are included in the manuscript and/or the **Supplementary Files**.

## Author Contributions

GF and BZ prepared the database. VA, AVA, MV, AAA, DB, and EL performed the computational experiments. VT performed substructure and data analysis. RY, IO, PS, DS, AC, AB, and AS performed the biological experiments. MP performed the IP analysis. YI, AZ, VK, and OD conceived and supervised the study. AM prepared the manuscript.

## Funding

The authors would like to kindly acknowledge the Ministry of Education and Science of the Russian Federation, government grant 20.9907.2017/VU (expert opinion, discussion, manuscript preparation, and substructure analysis) and Russian Science Foundation No. 17-74-30012, IBG RAS Ufa (biological evaluation, compound selection, and purchasing as well as *in silico* modeling).

## Conflict of Interest Statement

Authors YI, AZ, RY, VA, AA, VT, and MV were employed by Insilico Medicine, Inc. Author VK was employed by InterBioScreen ltd. The remaining authors declare that the research was conducted in the absence of any commercial or financial relationships that could be construed as a potential conflict of interest.
