# Supplementary material for: Identification of Novel Antibacterials Using Machine Learning Techniques
Source: Front Pharmacol. 2019 Aug 27;10:913. doi: 10.3389/fphar.2019.00913 (PMC6719509; doi:10.3389/fphar.2019.00913)
Supplement: Supplementary file 1 [file DataSheet_1.pdf]

Structures and activity of antibacterial molecules published in JMC during the past two years

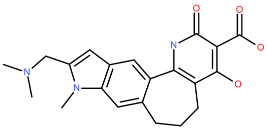

I

MIC_{90}=0.5 µg/mL (*E.coli*)
MIC_{90}=16 µg/mL (*Acinetobacter baumannii*)

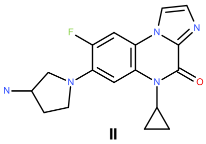

II

MIC=0.1-8 µg/mL (*Gram-Positive and Gram-Negative Strains*)

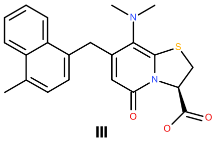

III

85% growth inhibition at 1 µM (L. monocytogenes)

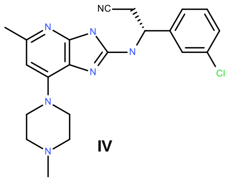

IV

IC_{50}=0.056 mM (PPAT, *E.Coli*)
MIC=2 µg/mL (*E.coli, ΔtolC*)
MIC=64 µg/mL (*WT E.Coli*)

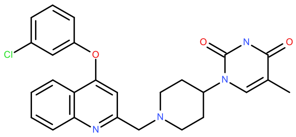

V

IC_{50}=0.95 µM (*Mt*TMPK)
MIC~16.35 µM (H37Rv, *Mycobacterium tuberculosis*)

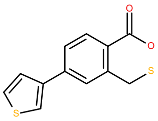

VI

MIC=8 µg/mL (*K. pneumoniae*, ATCC5055)

MIC=16 µg/mL (*E. coli*, MG1655)

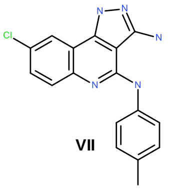

VII

IC_{50}=14.4 nM (*E. coli βG*)

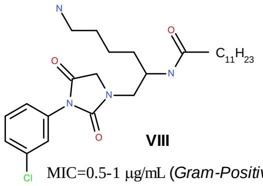

VIII

MIC=0.5-1 µg/mL (*Gram-Positive and Gram-Negative Strains*)

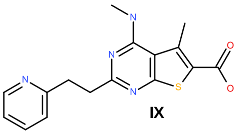

IX

IC_{50}=0.46 µM (*C. jejuni* PglD)

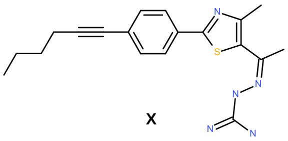

X

MIC=0.5 µg/mL (VRE Enterococcus faecium ATCC700221)

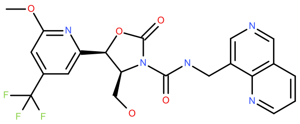

XI

IC_{50}=880 nM (*TarO*)

$MITC_{95}$=900 nM (*Staphylococcal aureus*)

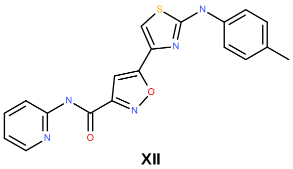

XII

MIC_{90}=0.06 µg/mL (*M. tuberculosis*)

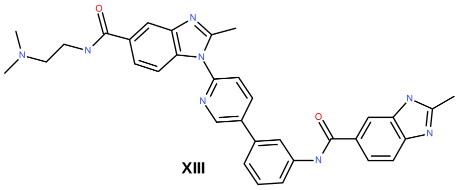

XIII

MIC=1-2 µg/mL (MDR Gram-positive MRSA and VRE bacteria)

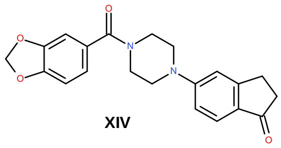

XIV

MIC=1.7 µM (*M. tuberculosis*)

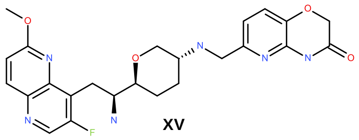

XV

IC_{50}=0.12 µM (DNA Gyrase, *P. aeruginosa*)

IC_{50}=0.03 µM (Topo IV, *P. aeruginosa*)

MIC=1 µg/mL (*P. aeruginosa*)

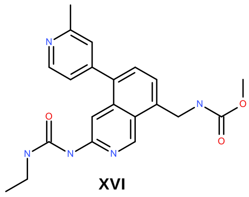

XVI

IC_{50}=0.03 µM (*E. coli* Gyrase)

IC_{50}=0.008 µM (*S. aureus* Gyrase)

MIC=0.06 µg/mL (*S. aureus*)

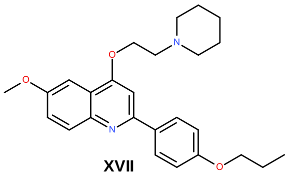

XVII

IC_{50}=4.2 µM (EtBr efflux)

MIC=238 µM (*S. aureus*)
