# Supplementary material for: Identification of Novel Antibacterials Using Machine Learning Techniques
Source: Front Pharmacol. 2019 Aug 27;10:913. doi: 10.3389/fphar.2019.00913 (PMC6719509; doi:10.3389/fphar.2019.00913)

Supplementary Figure 1. Characteristic HTS signals for the representative examples of the reported antibacterial compounds: *red aureole* - translation inhibitors and *green aureole* - SOS-response inducers)

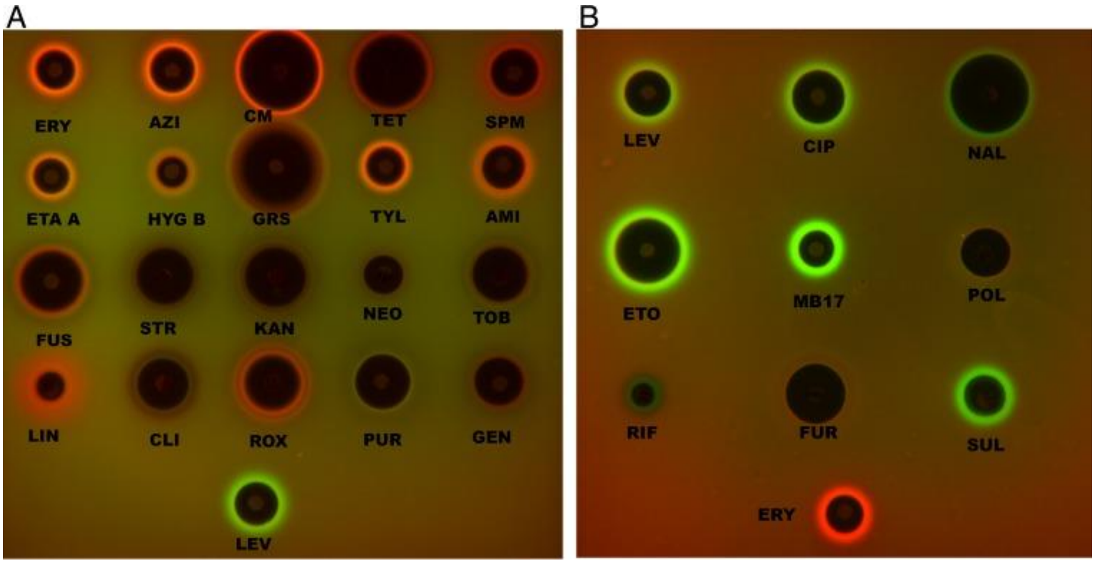

Supplement: Supplementary file 2 [file DataSheet_2.pdf]
