# Supplementary figures and images for: Identification of Novel Antibacterials Using Machine Learning Techniques
Source: Front Pharmacol. 2019 Aug 27;10:913. doi: 10.3389/fphar.2019.00913 (PMC6719509; doi:10.3389/fphar.2019.00913)

**Supplementary Figure 2.** Learning vector quantization error statistics for SOM training

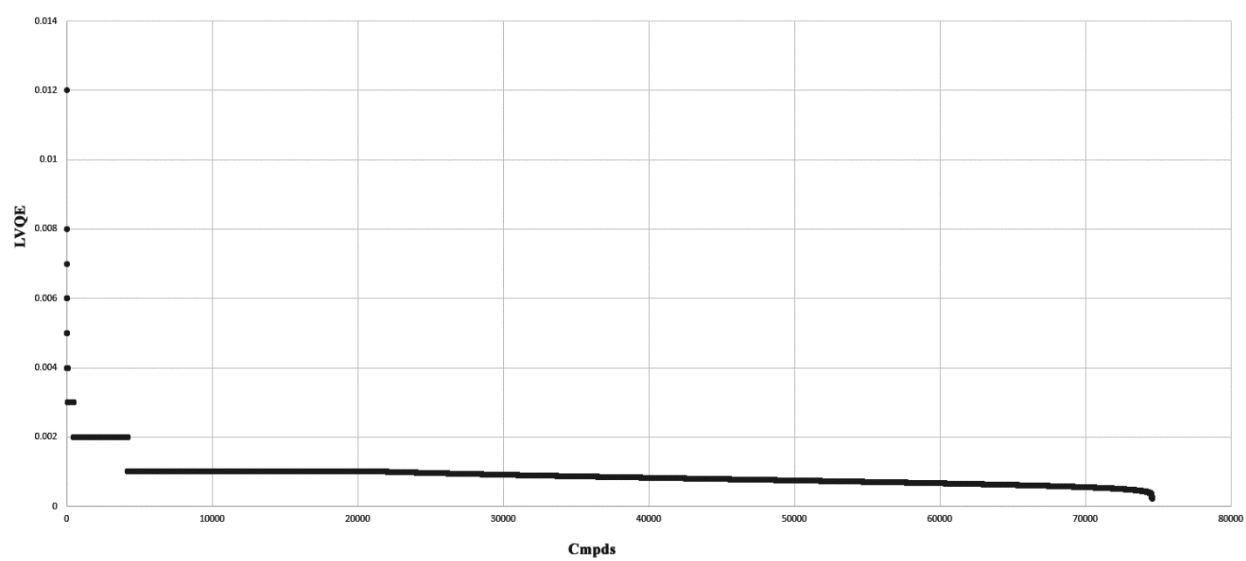

Supplement: Supplementary file 3 [file DataSheet_3.pdf]
