# Supplementary material for: Identification of Novel Antibacterials Using Machine Learning Techniques
Source: Front Pharmacol. 2019 Aug 27;10:913. doi: 10.3389/fphar.2019.00913 (PMC6719509; doi:10.3389/fphar.2019.00913)

Supplementary Figure 3. Biological details for the representative compounds

Compound 1.

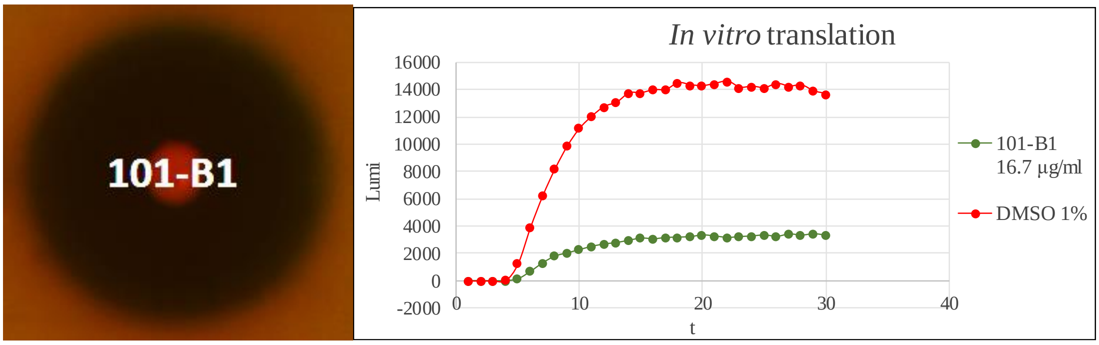

Compound 2.

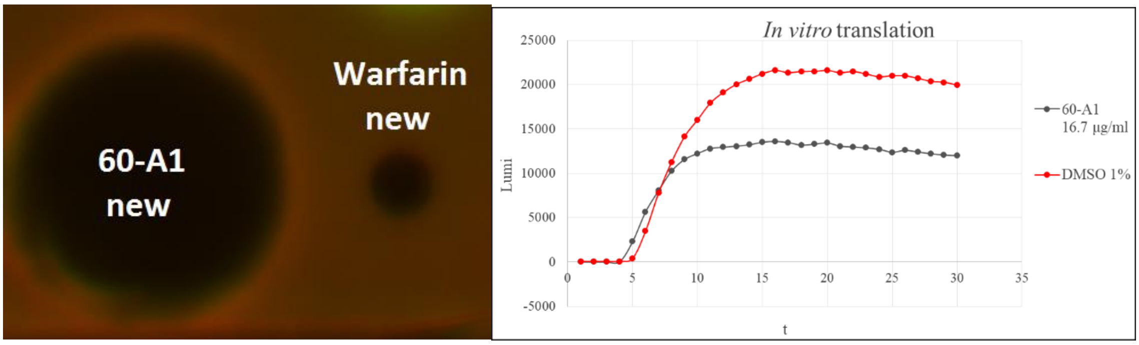

Compound 3.

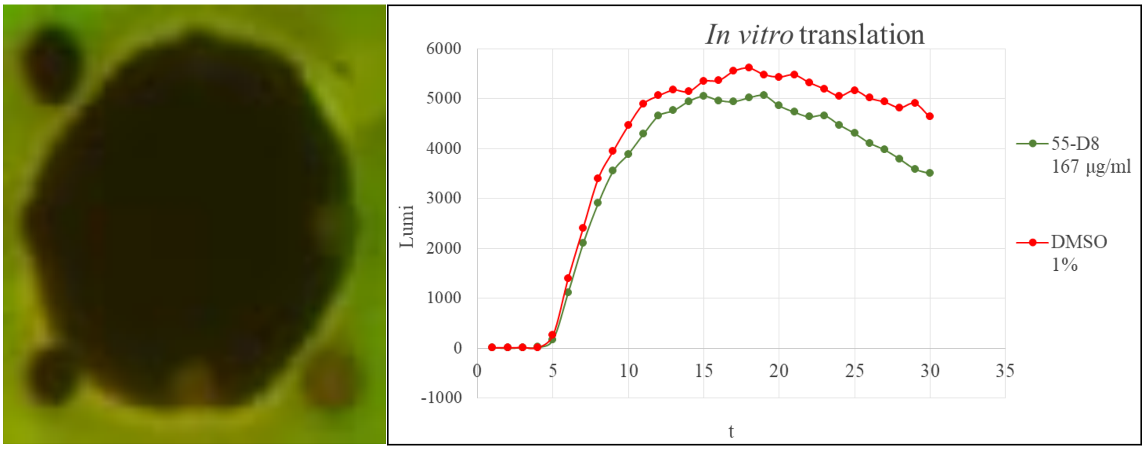

Compound 4.

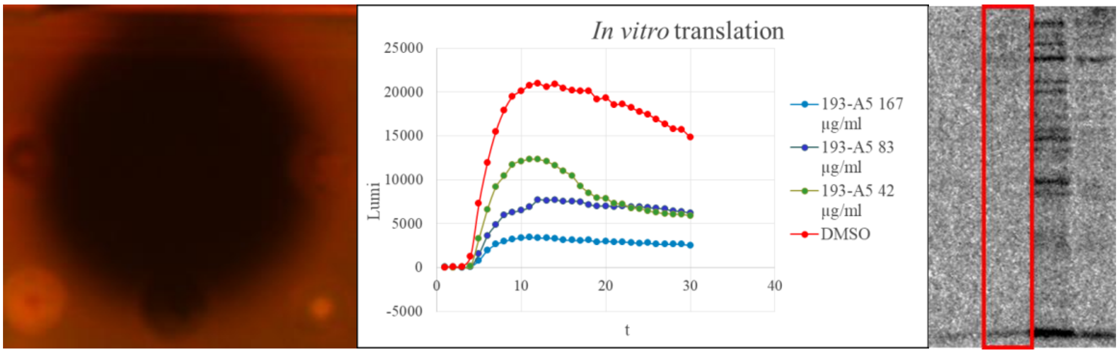

Compound 5.

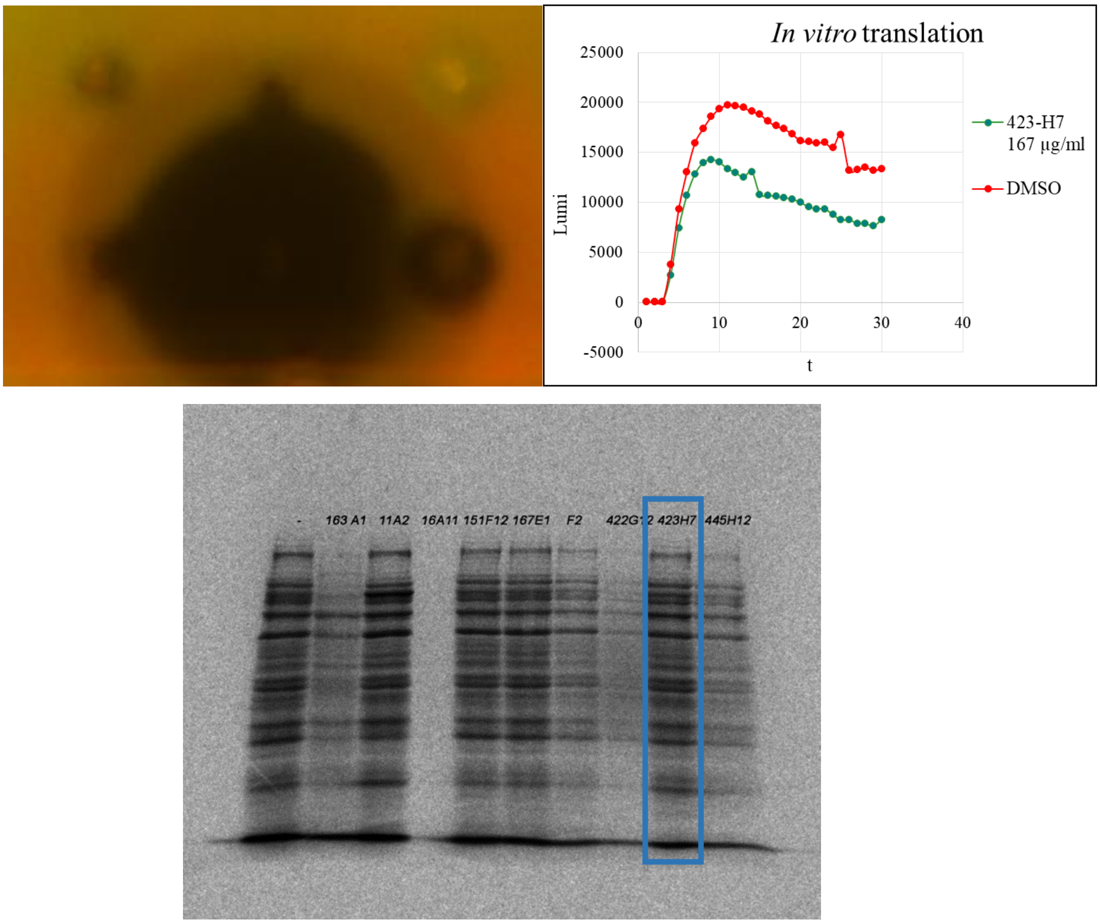

Compound 6.

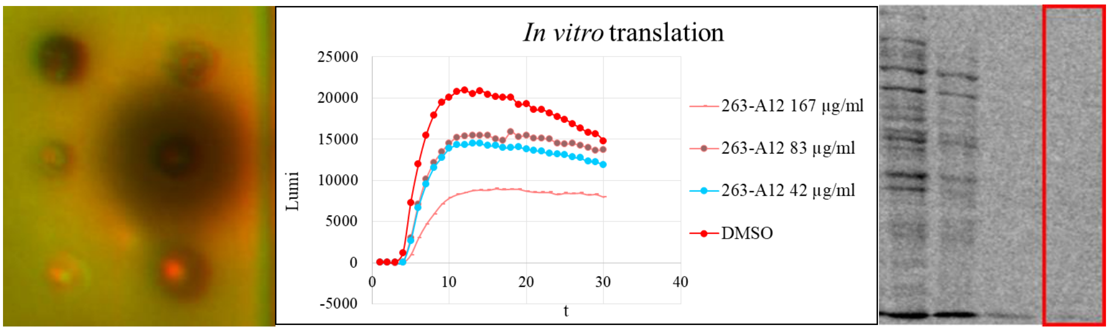

Compound 8.

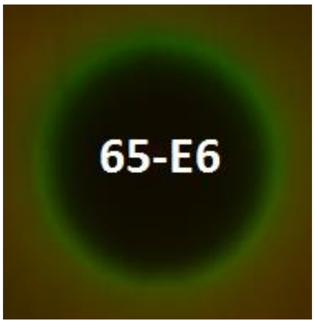

Supplement: Supplementary file 4 [file DataSheet_4.pdf]
