# Supplementary material for: Identification of Novel Antibacterials Using Machine Learning Techniques
Source: Front Pharmacol. 2019 Aug 27;10:913. doi: 10.3389/fphar.2019.00913 (PMC6719509; doi:10.3389/fphar.2019.00913)
Supplement: Supplementary file 6 [file Table_1.docx]

## **Supplementary Table 1**. Student *t*-values and PCA loadings for the selected descriptors

| **Descriptor** | ***t*-value (*module*)** | **Min** | **Max** | **Mean** | **SD** | **F1** | **F2** | **F3** | **F4** | **F5** | **F6** | **F7** | **F8** | **F9** | **F10** |
| --- | --- | --- | --- | --- | --- | --- | --- | --- | --- | --- | --- | --- | --- | --- | --- |
| **a_don** | 61.3 | 0.0 | 14.0 | 1.2 | 1.2 | 0.4 | 0.7 | 0.0 | 0.2 | 0.0 | -0.2 | 0.0 | -0.1 | -0.2 | 0.0 |
| **Hy** | 45.9 | -1.0 | 14.4 | -0.1 | 0.8 | 0.3 | 0.7 | 0.1 | 0.2 | 0.1 | -0.2 | 0.0 | 0.0 | -0.3 | 0.0 |
| **S(-OH)** | 44.2 | 0.0 | 99.1 | 2.6 | 6.2 | 0.4 | 0.6 | -0.1 | 0.0 | -0.4 | 0.1 | 0.3 | 0.1 | 0.3 | 0.2 |
| **nROH** | 44.0 | 0.0 | 10.0 | 0.1 | 0.4 | 0.3 | 0.6 | 0.0 | 0.0 | -0.3 | 0.0 | 0.2 | -0.2 | 0.2 | 0.0 |
| **O-061** | 43.0 | 0.0 | 5.0 | 0.1 | 0.4 | 0.1 | 0.2 | -0.3 | -0.2 | 0.2 | -0.2 | 0.1 | 0.7 | 0.0 | 0.2 |
| **ast_violation** | 41.2 | 0.0 | 4.0 | 2.1 | 0.9 | 0.7 | -0.3 | -0.1 | 0.2 | 0.1 | 0.1 | 0.1 | -0.1 | 0.1 | 0.1 |
| **SlogP_VSA0** | 39.6 | 0.0 | 271.9 | 20.3 | 21.5 | 0.4 | 0.4 | 0.2 | 0.3 | -0.2 | 0.1 | 0.0 | 0.0 | -0.4 | 0.0 |
| **GCUT_PEOE_2** | 38.6 | -0.1 | 0.3 | 0.0 | 0.1 | 0.1 | 0.3 | -0.5 | 0.4 | 0.1 | -0.1 | -0.4 | -0.2 | 0.1 | -0.2 |
| **SMR_VSA2** | 38.0 | 0.0 | 205.0 | 17.2 | 19.1 | 0.4 | 0.5 | -0.2 | -0.1 | 0.0 | -0.4 | 0.0 | 0.4 | -0.2 | -0.1 |
| **O-057** | 36.1 | 0.0 | 8.0 | 0.2 | 0.5 | 0.3 | 0.5 | 0.0 | 0.1 | -0.4 | 0.1 | 0.2 | -0.2 | 0.3 | 0.2 |
| **SPI** | 36.1 | 0.0 | 45.2 | 13.1 | 4.7 | 0.8 | -0.1 | 0.1 | -0.4 | 0.0 | 0.1 | 0.1 | 0.0 | -0.1 | 0.0 |
| **SS** | 35.7 | 14.0 | 149.3 | 64.1 | 15.5 | 0.9 | -0.2 | 0.0 | -0.2 | 0.1 | 0.0 | -0.1 | 0.0 | 0.1 | -0.1 |
| **HB2** | 35.7 | 0.0 | 11.5 | 1.9 | 0.9 | 0.5 | 0.4 | 0.3 | 0.1 | 0.4 | -0.3 | 0.0 | 0.0 | 0.1 | 0.1 |
| **EEig07x** | 35.6 | -0.8 | 4.1 | 2.9 | 0.5 | 0.8 | -0.4 | -0.2 | 0.1 | 0.0 | 0.0 | -0.1 | 0.0 | 0.1 | 0.1 |
| **GGI1** | 35.3 | 0.5 | 19.0 | 5.6 | 1.7 | 0.8 | -0.2 | 0.1 | -0.4 | 0.1 | 0.0 | 0.0 | 0.0 | 0.0 | 0.1 |
| **GATS1p** | 35.2 | 0.1 | 2.0 | 0.9 | 0.2 | 0.0 | -0.2 | 0.8 | 0.0 | 0.1 | -0.1 | -0.3 | -0.2 | 0.0 | 0.2 |
| **TPSA** | 35.1 | 0.0 | 367.9 | 76.2 | 28.1 | 0.7 | 0.4 | 0.3 | 0.2 | 0.3 | 0.0 | 0.0 | 0.2 | 0.1 | 0.0 |
| **BELe1** | 34.0 | 1.2 | 2.2 | 1.9 | 0.1 | 0.4 | -0.2 | -0.3 | 0.1 | -0.2 | 0.1 | -0.3 | 0.0 | 0.0 | 0.6 |
| **IC4** | 33.6 | 1.9 | 6.2 | 4.9 | 0.4 | 0.7 | -0.3 | -0.2 | 0.2 | -0.1 | -0.1 | -0.1 | -0.1 | -0.1 | -0.1 |
| **RB** | 33.6 | 0.0 | 25.0 | 3.5 | 2.4 | 0.6 | 0.1 | 0.2 | 0.1 | -0.2 | 0.1 | 0.3 | 0.0 | -0.2 | -0.3 |
| **GATS1v** | 33.5 | 0.1 | 1.9 | 0.9 | 0.2 | -0.1 | -0.3 | 0.8 | 0.3 | -0.1 | 0.0 | -0.1 | -0.1 | 0.0 | 0.0 |
| **TIE** | 33.3 | 2.5 | 709.6 | 54.6 | 25.0 | 0.9 | 0.0 | 0.1 | -0.4 | 0.0 | 0.0 | 0.0 | 0.0 | 0.0 | -0.1 |
| **Q'** | 33.0 | 0.0 | 0.7 | 0.1 | 0.0 | -0.8 | 0.4 | 0.0 | -0.1 | 0.0 | 0.1 | 0.0 | 0.0 | 0.1 | 0.1 |
| **a_acc** | 31.4 | 0.0 | 14.0 | 3.8 | 1.5 | 0.6 | 0.1 | 0.5 | 0.3 | 0.1 | 0.1 | 0.1 | 0.0 | 0.3 | 0.1 |
| **GCUT_SMR_1** | 31.1 | -0.3 | 0.1 | -0.2 | 0.0 | -0.2 | -0.3 | 0.3 | -0.5 | 0.1 | 0.0 | 0.5 | 0.1 | -0.1 | 0.2 |
| **M1** | 29.1 | 26.0 | 300.0 | 143.2 | 34.2 | 0.8 | -0.5 | -0.1 | 0.0 | 0.0 | -0.1 | 0.0 | 0.0 | 0.1 | 0.0 |
| **S(>N-)** | 28.8 | -1.4 | 14.1 | 1.8 | 1.8 | 0.0 | -0.5 | 0.2 | 0.0 | -0.2 | -0.6 | 0.1 | 0.0 | 0.3 | -0.1 |
| **VEA2** | 28.4 | 0.1 | 0.4 | 0.2 | 0.0 | -0.8 | 0.4 | 0.1 | -0.1 | 0.0 | 0.1 | 0.0 | 0.0 | 0.0 | 0.1 |
| **GATS1m** | 27.8 | 0.2 | 2.1 | 0.9 | 0.3 | 0.0 | -0.1 | 0.7 | 0.0 | 0.3 | 0.0 | -0.1 | -0.2 | -0.1 | 0.2 |
| **GVWAI-80** | 24.0 | 0.0 | 1.0 | 0.8 | 0.4 | -0.4 | 0.2 | 0.1 | 0.1 | 0.0 | 0.0 | -0.2 | 0.2 | 0.1 | 0.0 |
| **logS** | 22.8 | -15.9 | 1.7 | -5.0 | 1.9 | -0.5 | 0.4 | 0.6 | 0.0 | -0.2 | -0.2 | -0.1 | 0.0 | 0.1 | -0.1 |
| **SaaO** | 22.2 | 0.0 | 22.8 | 1.0 | 2.3 | 0.0 | -0.1 | 0.0 | 0.2 | 0.1 | 0.4 | 0.0 | 0.4 | 0.2 | -0.4 |
| **S(>CH-)** | 19.1 | -15.9 | 7.7 | -0.1 | 0.6 | -0.3 | -0.3 | -0.1 | 0.1 | 0.0 | -0.3 | 0.0 | 0.2 | -0.1 | 0.0 |
| **PEOE_VSA_FPOS** | 18.7 | 0.1 | 1.0 | 0.7 | 0.1 | -0.1 | -0.3 | 0.3 | 0.2 | -0.4 | -0.1 | 0.0 | 0.3 | 0.1 | 0.1 |
| **S(>C<)** | 12.7 | -14.2 | 2.2 | -0.3 | 1.0 | -0.2 | -0.1 | 0.1 | 0.5 | -0.1 | -0.1 | 0.1 | 0.1 | -0.2 | 0.2 |
| **S(-O-)** | 9.6 | 0.0 | 45.6 | 5.6 | 6.3 | 0.3 | -0.2 | 0.4 | 0.2 | -0.2 | 0.6 | 0.0 | 0.3 | -0.1 | -0.1 |
| **S(-S-)** | 9.6 | -0.4 | 10.7 | 0.5 | 0.9 | -0.1 | -0.1 | -0.2 | 0.1 | 0.4 | 0.0 | 0.6 | -0.3 | -0.2 | -0.1 |
| **S(=N-)** | 8.7 | 0.0 | 34.0 | 5.0 | 5.0 | -0.1 | -0.2 | 0.0 | 0.5 | 0.5 | -0.1 | 0.3 | 0.0 | 0.4 | 0.1 |
| **S(-C=)** | 7.8 | -17.7 | 16.5 | 3.0 | 2.8 | -0.1 | -0.5 | -0.3 | 0.4 | -0.1 | 0.0 | 0.3 | 0.0 | -0.2 | 0.3 |
| **S(-CH2-)** | 7.3 | -3.8 | 28.1 | 2.1 | 2.8 | -0.1 | -0.3 | 0.1 | 0.1 | -0.4 | -0.6 | 0.1 | 0.0 | 0.0 | -0.3 |
