# Supplementary material for: Identification of Novel Antibacterials Using Machine Learning Techniques
Source: Front Pharmacol. 2019 Aug 27;10:913. doi: 10.3389/fphar.2019.00913 (PMC6719509; doi:10.3389/fphar.2019.00913)
Supplement: Supplementary file 7 [file Table_2.docx]

## **Supplementary Table 2**. Molecular descriptors used for *in silico* study

| **Descriptor** | **Description** |
| --- | --- |
| HBD | Number of potential H-bond donors |
| Hy | Hydrophilicity index |
| S(-OH) | Partial electrotopological index of -OH |
| nROH | Number of hydroxyl groups |
| O-061 | O--(nitro, *N*-oxides) / atom-centred fragments |
| ast_violation | Astex fragment-like violation count |
| SlogP_VSA0 | Contribution of subdivided surface area in LogP |
| GCUT_PEOE_2 | PEOE change GCUT (2/3) |
| SMR_VSA2 | Contribution of subdivided surface area in molar refractivity |
| O-057 | The presence of phenol / enol / carboxyl OH groups |
| SPI | Superpendentic index |
| SS | Common electrotopological index |
| HB2 | Acceptor feature |
| EEig07x | Eigenvalue 07 from edge adj. matrix weighted by edge degrees |
| GGI1 | Topological charge index of order 1 |
| GATS1p | Geary autocorrelation of lag 1 weighted by polarizability |
| TPSA | Polar surface area |
| BELe1 | Lowest eigenvalue n. 1 of Burden matrix / weighted by atomic Sanderson electronegativities |
| IC4 | Information content index (neighborhood symmetry of 4-order) |
| RB | Number of free-rotatable bonds |
| GATS1v | Geary autocorrelation of lag 1 weighted by van der Waals volume |
| TIE | E-state topological parameter |
| Q' | Binormalized quadratic index |
| HBA | Number of potential H-bond acceptors |
| GCUT_SMR_1 | Molar refractivity GCUT (1/3) |
| M1 | Zagreb index 1 |
| S(>N-) | Partial electrotopological index of tertiary amine fragment |
| VEA2 | Average eigenvector coefficient sum from adjacency matrix |
| GATS1m | Geary autocorrelation of lag 1 weighted by mass |
| GVWAI-80 | Ghose-Viswanadhan-Wendoloski alert index at 80% (drug-like index) |
| logS | Log solubility in water |
| SaaO | Sum of aaO E-states |
| S(>CH-) | Partial electrotopological index of >CH- |
| PEOE_VSA_FPOS | Total polar positive VDW surface area |
| S(>C<) | Partial electrotopological index of >C< |
| S(-O-) | Partial electrotopological index of -O- |
| S(-S-) | Partial electrotopological index of -S- |
| S(=N-) | Partial electrotopological index of =N- |
| S(-C=) | Partial electrotopological index of -C= |
| S(-CH_2_-) | Partial electrotopological index of -CH_2_- |
