# Supplementary material for: Identification of Novel Antibacterials Using Machine Learning Techniques
Source: Front Pharmacol. 2019 Aug 27;10:913. doi: 10.3389/fphar.2019.00913 (PMC6719509; doi:10.3389/fphar.2019.00913)
Supplement: Supplementary file 8 [file Table_3.docx]

## **Supplementary Table 3**. Overall statistics for subsets using in training

| **Training set** | **Cross-validation set accuracy** | | | **Internal testing set accuracy** | | |
| --- | --- | --- | --- | --- | --- | --- |
|  | **Inactive, %** | **Active, %** | **Average, %** | **Inactive, %** | **Active, %** | **Average, %** |
| Random forest classifier | | | | | | |
| 1 | 92.2 | 68.1 | 80.2 | 91.2 | 71.8 | 81.5 |
| 2 | 93.8 | 72.4 | 83.1 | 91.9 | 72.2 | 82.1 |
| 3 | 92.2 | 70.8 | 81.5 | 92.0 | 71.4 | 81.7 |
| 4 | 92.9 | 71.5 | 82.2 | 91.5 | 73.0 | 82.3 |
| Gradient boosting classifier | | | | | | |
| 1 | 90.2 | 67.2 | 78.7 | 89.9 | 70.5 | 80.2 |
| 2 | 91.4 | 66.3 | 78.9 | 89.5 | 70.1 | 79.8 |
| 3 | 91.2 | 69.8 | 80.5 | 89.9 | 71.0 | 80.4 |
| 4 | 91.7 | 66.5 | 79.1 | 89.4 | 68.9 | 79.1 |
| Support vector classifier | | | | | | |
| 1 | 92.5 | 71.5 | 82.0 | 91.5 | 72.6 | 82.0 |
| 2 | 91.2 | 73.0 | 82.1 | 91.3 | 73.4 | 82.4 |
| 3 | 91.2 | 74.0 | 82.6 | 92.1 | 73.4 | 82.8 |
| 4 | 91.9 | 75.3 | 83.6 | 91.3 | 75.9 | 83.6 |
| K-nearest neighbors classifier | | | | | | |
| 1 | 89.2 | 78.9 | 84.0 | 88.7 | 77.2 | 83.0 |
| 2 | 86.7 | 77.9 | 82.3 | 88.1 | 78.8 | 83.4 |
| 3 | 87.7 | 77.6 | 82.7 | 88.6 | 77.2 | 82.9 |
| 4 | 87.8 | 77.1 | 82.4 | 89.8 | 77.6 | 83.7 |
