# Supplementary material for: Identification of Novel Antibacterials Using Machine Learning Techniques
Source: Front Pharmacol. 2019 Aug 27;10:913. doi: 10.3389/fphar.2019.00913 (PMC6719509; doi:10.3389/fphar.2019.00913)
Supplement: Supplementary file 9 [file Table_4.docx]

## **Supplementary Table 4**. Cytotoxicity of the selected compounds against eukaryotic cells

| **ID** | **Cell viability (10 μM)** | | | **CC_50_ (μg/ml)** | | | |
| --- | --- | --- | --- | --- | --- | --- | --- |
|  | **HEK293** | **A549** | **MCF7** | **HEK293T** | **A549** | **VA13** | **MCF7** |
| **1** | 87.53±10. 25 | 86.53±11.02 | 80.84±4.89 | 4.59±0.27 | 13.36±0.7 | 21.5±2.87 | n.d. |
| **2** | 95.69±13.88 | 105.90±4.55 | 95.96±1.47 | 31±2.3 | 29.1±2.6 | 34.6±1.6 | n.d. |
| **3** | 65.87±11.94 | 88.26±22.59 | 95.05±11.32 | 226.7±100 | 111±30 | >85 | n.d. |
| **4** | n.d. | n.d. | n.d. | >21 | >21 | >21 | >21 |
| **5** | n.d. | n.d. | n.d. | >21 | >21 | >21 | >21 |
| **6** | n.d. | n.d. | n.d. | >21 | >21 | >21 | >21 |
| **7** | 84.32±11.97 | 105.85±16.05 | 99.7±5.85 | n.d. | n.d. | n.d. | n.d. |
| **8** | 96.71±6.09 | 103.90±14.50 | 117.51±1.33 | n.d. | n.d. | n.d. | n.d. |
| **9** | 91.52±2.89 | 94.53±6.35 | 96.32±8.48 | n.d. | n.d. | n.d. | n.d. |
| **10** | 40.54±2.93 | 51.59±6.15 | 88.5±2.05 | n.d. | n.d. | n.d. | n.d. |
| **11** | 83.99±11.23 | 94.51±4.22 | 114.58±22.12 | n.d. | n.d. | n.d. | n.d. |
| **12** | 54.62±6.20 | 91.99±9.74 | 81.62±6.50 | n.d. | n.d. | n.d. | n.d. |
| **13** | 91.34 ± 2.74 | 93.88 ± 8.12 | 83.23 ± 6.88 | 6.27±0.64 | 11.42±0.5 | 19.48±1.09 | n.d. |
| **LVX** | >90 | n.d. | n.d. | 80±12 | n.d. | n.d. | n.d. |
| **ERY** | >90 | n.d. | n.d. | >200 | n.d. | n.d. | n.d. |

Lev and Ery were also tested against MRC-5 cell line and showed comparative results to HEK293; n.d. – not determined
