# Supplementary material for: Identification of Novel Antibacterials Using Machine Learning Techniques
Source: Front Pharmacol. 2019 Aug 27;10:913. doi: 10.3389/fphar.2019.00913 (PMC6719509; doi:10.3389/fphar.2019.00913)
Supplement: Supplementary file 10 [file Table_5.docx]

## **Supplementary Table 5**. SMILES of active compounds

| **ID** | **SMILES** |
| --- | --- |
|  |  |
| **1** | OC(=O)c1c(NC(=O)c2ccc(Br)cc2)scc1c3cccs3 |
| **2** | COC(=O)CC(C1=C(O)c2ccccc2OC1=O)c3ccc(OC)c(OCCc4cccc5OCCc45)c3 |
| **3** | COc1ccc2C(=O)\C(=C\c3cccc(OC)c3OC)\Oc2c1CN4CCOCC4 |
| **4** | OC(=O)c1ccn2c(C(=O)c3ccccc3)c(cc2c1)c4ccccc4 |
| **5** | Fc1ccccc1CC(=O)N2CC(C2)n3cc(COc4ccccc4)nn3 |
| **6** | CSc1nsc(NC(=O)c2ccccc2Br)n1 |
| **7** | COc1ccc(c(OC)c1)c2noc(n2)C3=CN(C)c4cc(N5CCOCC5)c(F)cc4C3=O |
| **8** | Cc1cccc2C(=O)c3c(O)cc(OCC(=O)O)cc3Oc12 |
| **9** | O=C1NC(=NC2=C1SC(=S)N2c3ccccc3)SCc4ccccc4 |
| **10** | CCCC1=NC(=O)CC(=N1)n2nc(C)cc2NC(=O)c3cc(Cl)ccc3[N+](=O)[O-] |
| **11** | Clc1ccc(Cc2nnc(NS(=O)(=O)c3ccc(Br)cc3)s2)cc1 |
| **12** | Cc1nc(sc1C)n2nc(C)c(CCC(=O)Nc3ccc(Br)cc3)c2O |
| **13** | O=C(O)C1=C(NC(C2=CC=C(F)C=C2)=O)SC=C1C3=CC=CS3 |
| **LVX** | C[C@H]1COc2c(N3CCN(C)CC3)c(F)cc4C(=O)C(=CN1c24)C(=O)O |
| **ERY** | CC[C@@H]1[C@@]([C@@H]([C@H](C(=O)[C@@H](C[C@@]  ([C@@H]([C@H]([C@@H]([C@H](C(=O)O1)C)O[C@H]2C[C@@]  ([C@H]([C@@H](O2)C)O)(C)OC)C)O[C@H]3[C@@H]([C@H]  (C[C@H](O3)C)N(C)C)O)(C)O)C)C)O)(C)O |
