# Supplementary material for: Identification of Novel Antibacterials Using Machine Learning Techniques
Source: Front Pharmacol. 2019 Aug 27;10:913. doi: 10.3389/fphar.2019.00913 (PMC6719509; doi:10.3389/fphar.2019.00913)

## **Supplementary Table 7**. PCA analysis details

| **Variable** | **Observations** | **Obs. with missing data** | **Obs. without missing data** | **Min.** | **Max.** | **Mean** | **SD** |
| --- | --- | --- | --- | --- | --- | --- | --- |
| Hy | 74567 | 0 | 74567 | -0,959 | 14,400 | -0,083 | 0,774 |
| S(-OH) | 74567 | 0 | 74567 | 0,000 | 99,100 | 2,554 | 6,159 |
| SS | 74567 | 0 | 74567 | 14,000 | 149,300 | 64,063 | 15,495 |
| HB2 | 74567 | 0 | 74567 | 0,000 | 11,500 | 1,900 | 0,884 |
| M1 | 74567 | 0 | 74567 | 26,000 | 300,000 | 143,248 | 34,209 |
| Q' | 74567 | 0 | 74567 | 0,000 | 0,667 | 0,076 | 0,031 |
| HBD | 74567 | 0 | 74567 | 0,000 | 14,000 | 1,217 | 1,216 |
| ast_violation | 74567 | 0 | 74567 | 0,000 | 4,000 | 2,069 | 0,905 |
| SlogP_VSA0 | 74567 | 0 | 74567 | 0,000 | 271,863 | 20,276 | 21,471 |
| GCUT_PEOE_2 | 74567 | 0 | 74567 | -0,136 | 0,255 | 0,044 | 0,051 |
| SMR_VSA2 | 74567 | 0 | 74567 | 0,000 | 204,979 | 17,174 | 19,081 |
| HBA | 74567 | 0 | 74567 | 0,000 | 14,000 | 3,841 | 1,510 |
| GCUT_SMR_1 | 74567 | 0 | 74567 | -0,305 | 0,077 | -0,197 | 0,027 |
| logS | 74567 | 0 | 74567 | -15,911 | 1,655 | -5,015 | 1,918 |
| RB | 74567 | 0 | 74567 | 0,000 | 25,000 | 3,540 | 2,398 |
| O-061 | 74567 | 0 | 74567 | 0,000 | 5,000 | 0,083 | 0,418 |
| nROH | 74567 | 0 | 74567 | 0,000 | 10,000 | 0,141 | 0,426 |
| O-057 | 74567 | 0 | 74567 | 0,000 | 8,000 | 0,151 | 0,476 |
| SPI | 74567 | 0 | 74567 | 0,000 | 45,236 | 13,109 | 4,699 |
| EEig07x | 74567 | 0 | 74567 | -0,848 | 4,117 | 2,905 | 0,481 |
| GGI1 | 74567 | 0 | 74567 | 0,500 | 19,000 | 5,570 | 1,663 |
| GATS1v | 74567 | 0 | 74567 | 0,149 | 1,875 | 0,862 | 0,156 |
| GATS1p | 74567 | 0 | 74567 | 0,144 | 2,040 | 0,906 | 0,236 |
| GATS1m | 74567 | 0 | 74567 | 0,175 | 2,113 | 0,852 | 0,250 |
| SaaO | 74567 | 0 | 74567 | 0,000 | 22,790 | 0,994 | 2,340 |
| BELe1 | 74567 | 0 | 74567 | 1,243 | 2,218 | 1,893 | 0,074 |
| IC4 | 74567 | 0 | 74567 | 1,918 | 6,154 | 4,944 | 0,384 |
| TIE | 74567 | 0 | 74567 | 2,475 | 709,639 | 54,630 | 25,032 |
| VEA2 | 74567 | 0 | 74567 | 0,078 | 0,400 | 0,163 | 0,030 |
| GVWAI-80 | 74567 | 0 | 74567 | 0,000 | 1,000 | 0,788 | 0,409 |
| TPSA | 74567 | 0 | 74567 | 0,000 | 367,870 | 76,216 | 28,105 |
| PEOE_VSA_FPOS | 74567 | 0 | 74567 | 0,094 | 1,000 | 0,750 | 0,113 |
| S(-CH2-) | 74567 | 0 | 74567 | -3,813 | 28,100 | 2,073 | 2,775 |
| S(-C=) | 74567 | 0 | 74567 | -17,700 | 16,500 | 3,020 | 2,778 |
| S(=N-) | 74567 | 0 | 74567 | 0,000 | 34,000 | 5,028 | 5,018 |
| S(-S-) | 74567 | 0 | 74567 | -0,406 | 10,700 | 0,507 | 0,859 |
| S(-O-) | 74567 | 0 | 74567 | 0,000 | 45,600 | 5,644 | 6,316 |
| S(>C<) | 74567 | 0 | 74567 | -14,200 | 2,234 | -0,280 | 1,029 |
| S(>CH-) | 74567 | 0 | 74567 | -15,900 | 7,708 | -0,087 | 0,638 |
| S(>N-) | 74567 | 0 | 74567 | -1,396 | 14,100 | 1,836 | 1,815 |

**Eigenvalues:**

|  | **Eigenvalue** | **Variability (%)** | **Cumulative %** |
| --- | --- | --- | --- |
| F1 | 9,649 | 24,123 | 24,123 |
| F2 | 5,431 | 13,578 | 37,702 |
| F3 | 3,648 | 9,119 | 46,821 |
| F4 | 2,203 | 5,507 | 52,328 |
| F5 | 1,974 | 4,935 | 57,262 |
| F6 | 1,863 | 4,658 | 61,920 |
| F7 | 1,527 | 3,817 | 65,738 |
| F8 | 1,440 | 3,601 | 69,339 |
| F9 | 1,228 | 3,071 | 72,410 |
| F10 | 1,174 | 2,935 | 75,345 |
| F11 | 1,099 | 2,748 | 78,093 |
| F12 | 0,948 | 2,369 | 80,461 |
| F13 | 0,925 | 2,313 | 82,774 |
| F14 | 0,775 | 1,937 | 84,711 |
| F15 | 0,721 | 1,802 | 86,513 |
| F16 | 0,602 | 1,506 | 88,019 |
| F17 | 0,589 | 1,473 | 89,492 |
| F18 | 0,514 | 1,284 | 90,776 |
| F19 | 0,483 | 1,206 | 91,982 |
| F20 | 0,381 | 0,952 | 92,935 |
| F21 | 0,322 | 0,805 | 93,739 |
| F22 | 0,299 | 0,746 | 94,486 |
| F23 | 0,293 | 0,732 | 95,218 |
| F24 | 0,271 | 0,676 | 95,894 |
| F25 | 0,234 | 0,585 | 96,479 |
| F26 | 0,223 | 0,558 | 97,037 |
| F27 | 0,185 | 0,462 | 97,499 |
| F28 | 0,159 | 0,398 | 97,897 |
| F29 | 0,149 | 0,371 | 98,268 |
| F30 | 0,114 | 0,285 | 98,553 |
| F31 | 0,097 | 0,243 | 98,796 |
| F32 | 0,089 | 0,221 | 99,017 |
| F33 | 0,082 | 0,204 | 99,221 |
| F34 | 0,073 | 0,183 | 99,404 |
| F35 | 0,067 | 0,166 | 99,571 |
| F36 | 0,056 | 0,139 | 99,710 |
| F37 | 0,040 | 0,100 | 99,810 |
| F38 | 0,032 | 0,080 | 99,890 |
| F39 | 0,030 | 0,076 | 99,966 |
| F40 | 0,014 | 0,034 | 100,000 |


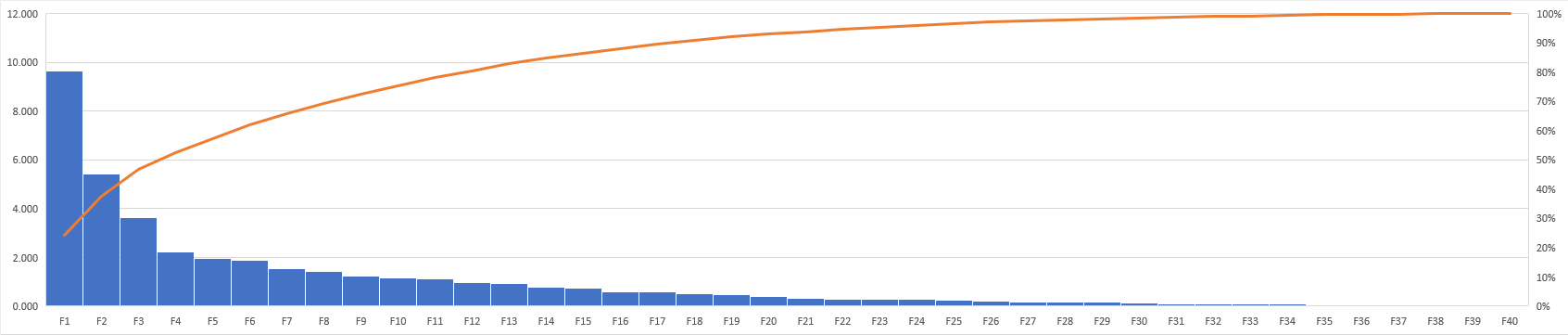


**Eigenvectors:**

|  | **Hy** | **S(-OH)** | **SS** | **HB2** | **M1** | **Q'** | **a_don** | **ast_violation** | **SlogP_VSA0** |
| --- | --- | --- | --- | --- | --- | --- | --- | --- | --- |
| F1 | 0,105 | 0,124 | 0,299 | 0,172 | 0,266 | -0,243 | 0,125 | 0,227 | 0,141 |
| F2 | 0,321 | 0,267 | -0,069 | 0,177 | -0,196 | 0,157 | 0,307 | -0,108 | 0,187 |
| F3 | 0,031 | -0,058 | 0,001 | 0,143 | -0,058 | 0,018 | 0,010 | -0,028 | 0,112 |
| F4 | 0,116 | 0,003 | -0,118 | 0,044 | 0,024 | -0,056 | 0,159 | 0,156 | 0,176 |
| F5 | 0,082 | -0,261 | 0,067 | 0,303 | -0,025 | 0,016 | -0,017 | 0,052 | -0,176 |
| F6 | -0,137 | 0,041 | 0,019 | -0,240 | -0,062 | 0,055 | -0,121 | 0,060 | 0,093 |
| F7 | -0,004 | 0,228 | -0,102 | -0,019 | -0,015 | 0,040 | -0,003 | 0,103 | -0,003 |
| F8 | 0,006 | 0,043 | -0,020 | 0,041 | -0,031 | -0,039 | -0,112 | -0,056 | 0,003 |
| F9 | -0,275 | 0,241 | 0,082 | 0,051 | 0,063 | 0,087 | -0,207 | 0,050 | -0,318 |
| F10 | 0,027 | 0,188 | -0,060 | 0,076 | 0,021 | 0,093 | 0,009 | 0,046 | 0,029 |
| F11 | -0,225 | 0,186 | 0,005 | -0,059 | -0,035 | -0,124 | -0,158 | -0,007 | -0,246 |
| F12 | -0,086 | -0,109 | -0,038 | 0,064 | -0,118 | -0,238 | -0,052 | 0,110 | -0,020 |
| F13 | 0,007 | 0,038 | -0,014 | -0,055 | -0,060 | -0,059 | 0,123 | 0,199 | -0,050 |
| F14 | 0,137 | -0,079 | -0,064 | 0,071 | -0,036 | -0,067 | 0,237 | 0,007 | -0,225 |
| F15 | -0,073 | -0,085 | 0,059 | 0,000 | -0,016 | 0,013 | -0,082 | 0,019 | -0,040 |
| F16 | -0,190 | -0,130 | -0,030 | 0,158 | -0,091 | -0,003 | 0,000 | -0,010 | -0,322 |
| F17 | 0,057 | -0,039 | 0,062 | -0,031 | 0,067 | 0,089 | 0,144 | -0,057 | -0,109 |
| F18 | 0,013 | -0,037 | -0,013 | 0,192 | 0,130 | 0,211 | -0,116 | -0,101 | 0,139 |
| F19 | -0,054 | -0,073 | -0,005 | -0,156 | 0,055 | 0,076 | 0,127 | 0,501 | -0,149 |
| F20 | 0,024 | 0,058 | 0,006 | 0,015 | 0,026 | 0,027 | -0,097 | -0,401 | 0,151 |
| F21 | -0,131 | -0,227 | 0,064 | -0,221 | 0,107 | 0,278 | 0,125 | -0,201 | -0,104 |
| F22 | 0,032 | 0,078 | -0,007 | 0,181 | 0,070 | 0,320 | 0,046 | 0,142 | -0,196 |
| F23 | 0,059 | -0,078 | 0,049 | 0,059 | -0,042 | 0,107 | -0,087 | 0,299 | 0,083 |
| F24 | -0,003 | -0,007 | -0,012 | 0,124 | -0,016 | -0,219 | 0,199 | -0,398 | -0,371 |
| F25 | -0,169 | 0,102 | 0,061 | -0,052 | 0,078 | -0,042 | -0,155 | -0,205 | 0,347 |
| F26 | -0,074 | -0,020 | 0,007 | 0,045 | 0,107 | 0,455 | -0,062 | -0,092 | -0,044 |
| F27 | -0,025 | -0,074 | 0,062 | -0,066 | 0,121 | -0,022 | 0,127 | -0,168 | -0,156 |
| F28 | -0,206 | 0,033 | -0,163 | 0,175 | -0,156 | -0,158 | -0,105 | 0,043 | 0,081 |
| F29 | -0,211 | 0,041 | 0,022 | 0,617 | 0,153 | 0,170 | -0,169 | 0,051 | 0,078 |
| F30 | 0,062 | 0,037 | -0,071 | -0,188 | -0,004 | 0,406 | 0,019 | 0,068 | -0,014 |
| F31 | -0,126 | 0,086 | 0,020 | -0,165 | -0,016 | 0,076 | 0,099 | -0,079 | 0,078 |
| F32 | -0,165 | -0,009 | -0,052 | -0,091 | -0,069 | -0,043 | -0,127 | 0,032 | 0,078 |
| F33 | 0,022 | -0,046 | -0,047 | 0,170 | -0,137 | -0,139 | -0,030 | -0,022 | 0,008 |
| F34 | 0,173 | 0,038 | 0,313 | -0,034 | 0,342 | -0,134 | 0,077 | -0,028 | -0,062 |
| F35 | 0,242 | -0,203 | 0,106 | -0,099 | -0,033 | 0,019 | -0,327 | 0,016 | -0,080 |
| F36 | 0,048 | 0,082 | 0,014 | 0,130 | -0,050 | 0,148 | 0,295 | -0,051 | -0,038 |
| F37 | 0,441 | -0,100 | -0,297 | 0,019 | -0,335 | 0,054 | -0,325 | -0,034 | -0,130 |
| F38 | 0,095 | 0,656 | -0,146 | -0,067 | -0,013 | -0,039 | -0,074 | 0,010 | -0,232 |
| F39 | -0,391 | -0,100 | -0,343 | 0,024 | -0,220 | 0,010 | 0,373 | 0,025 | 0,127 |
| F40 | -0,081 | 0,098 | 0,681 | 0,024 | -0,648 | 0,113 | 0,056 | -0,001 | 0,018 |

|  | **GCUT_PEOE_2** | | | **SMR_VSA2** | | **a_acc** | | **GCUT_SMR_1** | | | **logS** | **RB** | **O-061** | | **nROH** | | **O-057** | |
| --- | --- | --- | --- | --- | --- | --- | --- | --- | --- | --- | --- | --- | --- | --- | --- | --- | --- | --- |
| F1 | 0,035 | | | 0,120 | | 0,189 | | -0,080 | | | -0,156 | 0,183 | 0,042 | | 0,093 | | 0,093 | |
| F2 | 0,146 | | | 0,217 | | 0,057 | | -0,117 | | | 0,182 | 0,032 | 0,068 | | 0,240 | | 0,226 | |
| F3 | -0,274 | | | -0,081 | | 0,262 | | 0,182 | | | 0,291 | 0,111 | -0,131 | | 0,004 | | -0,023 | |
| F4 | 0,248 | | | -0,069 | | 0,209 | | -0,305 | | | -0,029 | 0,080 | -0,142 | | 0,009 | | 0,063 | |
| F5 | 0,102 | | | 0,026 | | 0,101 | | 0,043 | | | -0,132 | -0,140 | 0,126 | | -0,244 | | -0,281 | |
| F6 | -0,078 | | | -0,276 | | 0,054 | | 0,001 | | | -0,175 | 0,081 | -0,131 | | 0,011 | | 0,100 | |
| F7 | -0,327 | | | -0,010 | | 0,112 | | 0,402 | | | -0,074 | 0,233 | 0,071 | | 0,188 | | 0,182 | |
| F8 | -0,172 | | | 0,327 | | -0,038 | | 0,059 | | | 0,005 | -0,011 | 0,598 | | -0,144 | | -0,136 | |
| F9 | 0,091 | | | -0,147 | | 0,235 | | -0,102 | | | 0,077 | -0,207 | 0,016 | | 0,225 | | 0,230 | |
| F10 | -0,140 | | | -0,061 | | 0,058 | | 0,185 | | | -0,068 | -0,305 | 0,194 | | 0,010 | | 0,149 | |
| F11 | 0,166 | | | 0,080 | | -0,094 | | -0,103 | | | 0,003 | 0,183 | 0,231 | | 0,153 | | 0,148 | |
| F12 | -0,130 | | | -0,051 | | 0,090 | | 0,043 | | | 0,185 | 0,135 | -0,066 | | -0,019 | | -0,132 | |
| F13 | -0,062 | | | 0,013 | | 0,023 | | 0,043 | | | -0,039 | 0,007 | -0,141 | | -0,058 | | 0,229 | |
| F14 | -0,103 | | | 0,000 | | -0,212 | | 0,309 | | | -0,039 | 0,018 | -0,296 | | 0,161 | | -0,025 | |
| F15 | 0,007 | | | -0,167 | | 0,166 | | -0,018 | | | 0,003 | 0,039 | -0,105 | | -0,322 | | 0,224 | |
| F16 | -0,049 | | | 0,149 | | 0,108 | | -0,109 | | | -0,014 | 0,293 | -0,109 | | 0,378 | | -0,299 | |
| F17 | 0,052 | | | 0,022 | | -0,066 | | -0,108 | | | -0,095 | -0,330 | -0,129 | | -0,002 | | 0,074 | |
| F18 | -0,163 | | | -0,194 | | 0,154 | | -0,058 | | | 0,014 | -0,265 | -0,114 | | 0,101 | | -0,220 | |
| F19 | -0,039 | | | 0,247 | | 0,133 | | -0,055 | | | -0,031 | -0,355 | 0,006 | | 0,285 | | -0,131 | |
| F20 | 0,015 | | | -0,091 | | 0,020 | | -0,098 | | | 0,069 | -0,014 | -0,043 | | 0,406 | | -0,258 | |
| F21 | -0,170 | | | 0,523 | | 0,245 | | 0,024 | | | 0,177 | 0,024 | -0,196 | | -0,164 | | 0,188 | |
| F22 | 0,120 | | | -0,314 | | -0,004 | | 0,217 | | | 0,179 | 0,039 | 0,138 | | -0,057 | | -0,016 | |
| F23 | 0,305 | | | 0,107 | | 0,020 | | 0,349 | | | -0,139 | 0,057 | -0,025 | | 0,075 | | -0,136 | |
| F24 | 0,005 | | | -0,050 | | 0,042 | | 0,132 | | | -0,123 | -0,160 | 0,057 | | 0,025 | | 0,179 | |
| F25 | 0,229 | | | 0,229 | | 0,025 | | 0,425 | | | -0,227 | -0,166 | -0,140 | | 0,097 | | -0,032 | |
| F26 | 0,163 | | | 0,080 | | -0,103 | | -0,158 | | | -0,263 | 0,256 | 0,029 | | -0,055 | | 0,054 | |
| F27 | 0,432 | | | -0,078 | | 0,250 | | 0,269 | | | -0,034 | 0,164 | -0,031 | | -0,034 | | -0,011 | |
| F28 | 0,314 | | | 0,134 | | 0,078 | | 0,065 | | | 0,507 | -0,046 | -0,038 | | -0,127 | | 0,041 | |
| F29 | -0,144 | | | 0,159 | | -0,155 | | -0,048 | | | -0,037 | 0,012 | -0,198 | | -0,046 | | 0,191 | |
| F30 | 0,027 | | | -0,056 | | -0,078 | | 0,079 | | | 0,167 | 0,222 | 0,068 | | 0,013 | | -0,123 | |
| F31 | -0,141 | | | -0,050 | | 0,380 | | -0,085 | | | -0,086 | 0,040 | 0,112 | | -0,050 | | -0,119 | |
| F32 | 0,076 | | | 0,044 | | 0,133 | | 0,002 | | | 0,027 | -0,165 | -0,030 | | 0,050 | | -0,024 | |
| F33 | -0,116 | | | 0,034 | | 0,142 | | -0,072 | | | -0,357 | 0,192 | -0,035 | | -0,037 | | 0,042 | |
| F34 | -0,029 | | | -0,045 | | -0,070 | | 0,018 | | | 0,258 | 0,001 | 0,034 | | 0,019 | | -0,041 | |
| F35 | 0,008 | | | 0,065 | | -0,309 | | -0,010 | | | 0,094 | -0,004 | -0,108 | | 0,159 | | 0,216 | |
| F36 | 0,045 | | | -0,047 | | 0,080 | | -0,001 | | | -0,051 | -0,037 | 0,078 | | -0,067 | | -0,079 | |
| F37 | 0,054 | | | 0,052 | | 0,279 | | -0,013 | | | -0,117 | -0,086 | -0,004 | | 0,035 | | 0,127 | |
| F38 | -0,016 | | | 0,177 | | -0,075 | | -0,045 | | | -0,042 | -0,009 | -0,370 | | -0,321 | | -0,343 | |
| F39 | 0,020 | | | -0,070 | | -0,260 | | -0,013 | | | -0,071 | -0,071 | 0,034 | | 0,029 | | 0,027 | |
| F40 | -0,013 | | | -0,013 | | -0,045 | | 0,006 | | | -0,003 | -0,054 | -0,052 | | -0,028 | | -0,046 | |
|  | | **SPI** | **EEig07x** | | **GGI1** | | **GATS1v** | | **GATS1p** | **GATS1m** | | **SaaO** | | **BELe1** | | **IC4** | |  |
| F1 | | 0,265 | 0,247 | | 0,254 | | -0,031 | | 0,004 | 0,001 | | -0,007 | | 0,118 | | 0,232 | |  |
| F2 | | -0,029 | -0,187 | | -0,069 | | -0,143 | | -0,093 | -0,046 | | -0,047 | | -0,104 | | -0,140 | |  |
| F3 | | 0,064 | -0,112 | | 0,054 | | 0,395 | | 0,404 | 0,354 | | 0,020 | | -0,154 | | -0,098 | |  |
| F4 | | -0,266 | 0,065 | | -0,248 | | 0,181 | | -0,029 | -0,010 | | 0,145 | | 0,043 | | 0,140 | |  |
| F5 | | -0,024 | 0,019 | | 0,051 | | -0,060 | | 0,086 | 0,198 | | 0,100 | | -0,161 | | -0,060 | |  |
| F6 | | 0,048 | -0,010 | | 0,029 | | -0,021 | | -0,048 | 0,001 | | 0,296 | | 0,056 | | -0,095 | |  |
| F7 | | 0,066 | -0,086 | | 0,013 | | -0,055 | | -0,237 | -0,052 | | -0,002 | | -0,236 | | -0,077 | |  |
| F8 | | 0,036 | 0,011 | | -0,016 | | -0,059 | | -0,126 | -0,136 | | 0,309 | | -0,039 | | -0,057 | |  |
| F9 | | -0,047 | 0,051 | | -0,015 | | -0,032 | | -0,042 | -0,121 | | 0,195 | | -0,021 | | -0,053 | |  |
| F10 | | 0,036 | 0,102 | | 0,072 | | 0,004 | | 0,178 | 0,139 | | -0,377 | | 0,540 | | -0,112 | |  |
| F11 | | -0,073 | 0,027 | | -0,128 | | -0,077 | | 0,229 | 0,410 | | 0,114 | | 0,019 | | 0,105 | |  |
| F12 | | 0,047 | 0,111 | | -0,133 | | -0,076 | | -0,231 | -0,202 | | -0,350 | | -0,033 | | 0,080 | |  |
| F13 | | -0,007 | 0,050 | | 0,160 | | -0,135 | | 0,026 | 0,132 | | 0,101 | | 0,120 | | -0,048 | |  |
| F14 | | 0,110 | 0,055 | | 0,086 | | -0,025 | | 0,080 | -0,141 | | 0,486 | | 0,068 | | 0,077 | |  |
| F15 | | 0,158 | -0,035 | | 0,000 | | -0,116 | | -0,074 | -0,246 | | -0,172 | | -0,134 | | -0,099 | |  |
| F16 | | -0,001 | -0,140 | | -0,006 | | -0,120 | | -0,030 | 0,007 | | -0,195 | | 0,167 | | -0,026 | |  |
| F17 | | 0,106 | 0,087 | | 0,093 | | -0,036 | | -0,115 | 0,112 | | -0,005 | | -0,217 | | -0,057 | |  |
| F18 | | -0,072 | 0,015 | | 0,076 | | -0,326 | | -0,130 | 0,024 | | 0,153 | | 0,227 | | -0,026 | |  |
| F19 | | -0,033 | 0,010 | | 0,138 | | 0,098 | | 0,090 | -0,088 | | -0,129 | | -0,301 | | -0,100 | |  |
| F20 | | 0,157 | 0,141 | | 0,125 | | 0,066 | | -0,042 | -0,031 | | 0,011 | | -0,018 | | 0,066 | |  |
| F21 | | -0,054 | 0,047 | | -0,077 | | -0,089 | | -0,099 | 0,092 | | 0,047 | | 0,212 | | 0,261 | |  |
| F22 | | -0,074 | -0,103 | | 0,008 | | -0,089 | | -0,005 | -0,012 | | -0,079 | | -0,125 | | 0,633 | |  |
| F23 | | -0,054 | -0,118 | | -0,246 | | 0,194 | | -0,033 | -0,181 | | 0,089 | | 0,383 | | -0,097 | |  |
| F24 | | -0,119 | 0,162 | | -0,058 | | 0,414 | | -0,034 | -0,254 | | -0,093 | | 0,053 | | 0,017 | |  |
| F25 | | -0,171 | 0,050 | | 0,021 | | -0,096 | | 0,125 | -0,003 | | -0,114 | | -0,255 | | 0,230 | |  |
| F26 | | 0,200 | -0,155 | | 0,337 | | 0,295 | | 0,090 | -0,187 | | -0,079 | | -0,001 | | -0,023 | |  |
| F27 | | 0,021 | -0,043 | | -0,002 | | -0,264 | | -0,056 | 0,175 | | -0,067 | | -0,063 | | -0,409 | |  |
| F28 | | 0,115 | 0,127 | | 0,406 | | -0,021 | | 0,099 | -0,217 | | 0,142 | | 0,081 | | -0,049 | |  |
| F29 | | -0,133 | 0,071 | | -0,251 | | 0,030 | | 0,000 | -0,029 | | 0,042 | | -0,119 | | -0,197 | |  |
| F30 | | -0,161 | 0,722 | | -0,029 | | 0,065 | | 0,004 | -0,012 | | -0,034 | | -0,063 | | -0,182 | |  |
| F31 | | -0,129 | -0,153 | | -0,071 | | -0,086 | | 0,447 | -0,392 | | 0,099 | | -0,007 | | -0,014 | |  |
| F32 | | 0,423 | 0,136 | | -0,418 | | 0,173 | | -0,048 | 0,040 | | 0,054 | | -0,053 | | 0,087 | |  |
| F33 | | -0,137 | 0,340 | | 0,065 | | -0,192 | | 0,190 | -0,046 | | -0,033 | | -0,082 | | 0,053 | |  |
| F34 | | -0,274 | -0,002 | | -0,113 | | -0,081 | | 0,195 | -0,182 | | 0,014 | | -0,013 | | -0,100 | |  |
| F35 | | 0,174 | -0,001 | | -0,135 | | -0,242 | | 0,389 | -0,191 | | -0,127 | | -0,010 | | -0,004 | |  |
| F36 | | 0,505 | 0,121 | | -0,329 | | -0,178 | | 0,206 | -0,026 | | 0,045 | | -0,025 | | 0,015 | |  |
| F37 | | -0,095 | 0,036 | | 0,054 | | -0,029 | | 0,020 | 0,010 | | 0,023 | | -0,022 | | 0,100 | |  |
| F38 | | 0,018 | -0,035 | | -0,021 | | -0,017 | | 0,062 | -0,009 | | -0,076 | | 0,016 | | -0,003 | |  |
| F39 | | -0,098 | 0,005 | | 0,014 | | -0,170 | | 0,164 | -0,040 | | -0,062 | | 0,012 | | 0,035 | |  |
| F40 | | -0,097 | 0,032 | | 0,041 | | 0,034 | | -0,061 | 0,016 | | 0,000 | | 0,017 | | 0,021 | |  |

|  | **TIE** | **VEA2** | **GVWAI-80** | **TPSA** | **PEOE_VSA_FPOS** | **S(-CH2-)** | **S(-C=)** | **S(=N-)** |
| --- | --- | --- | --- | --- | --- | --- | --- | --- |
| F1 | 0,277 | -0,261 | -0,131 | 0,217 | -0,017 | -0,023 | -0,039 | -0,023 |
| F2 | 0,018 | 0,192 | 0,080 | 0,184 | -0,145 | -0,135 | -0,226 | -0,066 |
| F3 | 0,065 | 0,051 | 0,075 | 0,155 | 0,181 | 0,055 | -0,174 | 0,009 |
| F4 | -0,256 | -0,055 | 0,068 | 0,111 | 0,167 | 0,073 | 0,285 | 0,320 |
| F5 | -0,028 | 0,020 | 0,011 | 0,220 | -0,277 | -0,275 | -0,056 | 0,379 |
| F6 | -0,019 | 0,089 | 0,036 | -0,027 | -0,060 | -0,436 | 0,000 | -0,075 |
| F7 | -0,038 | 0,024 | -0,169 | 0,008 | -0,026 | 0,110 | 0,226 | 0,228 |
| F8 | -0,023 | 0,003 | 0,154 | 0,186 | 0,275 | 0,011 | 0,037 | 0,016 |
| F9 | 0,000 | 0,044 | 0,060 | 0,071 | 0,088 | -0,044 | -0,212 | 0,372 |
| F10 | -0,103 | 0,061 | 0,044 | 0,005 | 0,066 | -0,234 | 0,247 | 0,101 |
| F11 | 0,053 | -0,052 | 0,148 | -0,042 | -0,143 | 0,005 | -0,107 | -0,241 |
| F12 | 0,015 | -0,107 | 0,632 | -0,015 | 0,059 | -0,144 | -0,072 | 0,017 |
| F13 | -0,015 | -0,023 | 0,404 | -0,027 | 0,028 | 0,186 | -0,178 | 0,055 |
| F14 | 0,012 | -0,026 | 0,120 | 0,014 | -0,029 | -0,092 | 0,112 | -0,008 |
| F15 | 0,101 | 0,053 | -0,197 | 0,130 | -0,106 | -0,115 | -0,254 | -0,049 |
| F16 | 0,046 | 0,044 | -0,269 | -0,069 | 0,271 | 0,001 | -0,055 | 0,128 |
| F17 | 0,050 | 0,019 | 0,004 | 0,058 | 0,687 | -0,163 | -0,054 | -0,189 |
| F18 | 0,094 | 0,049 | 0,092 | 0,122 | -0,113 | 0,485 | -0,092 | -0,118 |
| F19 | -0,109 | 0,023 | 0,058 | -0,059 | -0,256 | 0,018 | 0,093 | -0,195 |
| F20 | -0,101 | -0,036 | 0,108 | 0,090 | -0,149 | -0,355 | 0,083 | -0,056 |
| F21 | -0,030 | 0,043 | -0,006 | 0,038 | -0,112 | -0,142 | 0,037 | 0,031 |
| F22 | -0,065 | 0,080 | 0,003 | 0,104 | 0,067 | -0,061 | 0,106 | -0,212 |
| F23 | 0,032 | 0,103 | -0,020 | 0,072 | 0,088 | -0,093 | -0,303 | -0,177 |
| F24 | -0,043 | -0,060 | -0,023 | 0,109 | -0,133 | 0,180 | -0,105 | -0,105 |
| F25 | -0,004 | -0,045 | 0,064 | -0,129 | 0,077 | 0,040 | -0,174 | 0,204 |
| F26 | 0,140 | 0,149 | 0,344 | -0,057 | -0,009 | 0,138 | 0,111 | 0,138 |
| F27 | -0,010 | -0,106 | 0,129 | 0,147 | 0,020 | 0,074 | 0,345 | -0,156 |
| F28 | -0,119 | 0,084 | -0,136 | -0,154 | 0,040 | 0,027 | 0,130 | -0,048 |
| F29 | 0,025 | -0,241 | 0,019 | -0,092 | -0,033 | -0,129 | 0,195 | -0,213 |
| F30 | 0,066 | -0,116 | -0,084 | 0,066 | 0,014 | 0,040 | -0,142 | 0,036 |
| F31 | -0,023 | -0,280 | 0,020 | 0,082 | 0,085 | -0,045 | 0,054 | -0,198 |
| F32 | 0,306 | 0,313 | 0,013 | 0,200 | -0,045 | 0,143 | 0,268 | -0,060 |
| F33 | -0,201 | 0,599 | 0,000 | 0,004 | 0,050 | 0,020 | 0,024 | -0,182 |
| F34 | 0,442 | 0,341 | 0,072 | -0,247 | 0,018 | -0,102 | 0,142 | 0,135 |
| F35 | -0,200 | -0,089 | -0,006 | 0,358 | 0,043 | 0,110 | 0,062 | 0,123 |
| F36 | -0,139 | -0,080 | 0,005 | -0,491 | -0,010 | 0,065 | -0,134 | 0,130 |
| F37 | 0,426 | -0,210 | -0,004 | -0,253 | 0,022 | -0,056 | 0,112 | -0,026 |
| F38 | 0,048 | 0,005 | -0,002 | 0,187 | -0,026 | -0,013 | 0,022 | 0,015 |
| F39 | 0,411 | -0,042 | -0,035 | 0,237 | -0,009 | -0,051 | 0,032 | 0,145 |
| F40 | 0,037 | -0,050 | -0,001 | -0,002 | -0,004 | 0,090 | 0,187 | 0,018 |

|  | **S(-S-)** | **S(-O-)** | **S(>C<)** | **S(>CH-)** | **S(>N-)** |
| --- | --- | --- | --- | --- | --- |
| F1 | -0,018 | 0,093 | -0,068 | -0,111 | 0,007 |
| F2 | -0,055 | -0,087 | -0,023 | -0,143 | -0,210 |
| F3 | -0,110 | 0,185 | 0,041 | -0,046 | 0,096 |
| F4 | 0,039 | 0,152 | 0,323 | 0,081 | -0,032 |
| F5 | 0,293 | -0,145 | -0,046 | -0,024 | -0,167 |
| F6 | 0,018 | 0,452 | -0,094 | -0,196 | -0,414 |
| F7 | 0,462 | -0,003 | 0,080 | 0,029 | 0,089 |
| F8 | -0,240 | 0,264 | 0,123 | 0,140 | -0,037 |
| F9 | -0,202 | -0,089 | -0,192 | -0,106 | 0,294 |
| F10 | -0,093 | -0,100 | 0,190 | -0,024 | -0,089 |
| F11 | 0,258 | -0,037 | 0,399 | -0,064 | 0,001 |
| F12 | 0,085 | -0,016 | 0,050 | -0,268 | 0,023 |
| F13 | 0,075 | -0,058 | -0,231 | 0,635 | -0,235 |
| F14 | -0,251 | -0,288 | 0,302 | -0,126 | 0,005 |
| F15 | -0,150 | -0,009 | 0,566 | 0,293 | 0,016 |
| F16 | -0,110 | 0,032 | 0,046 | 0,119 | -0,355 |
| F17 | 0,344 | -0,059 | 0,092 | -0,057 | 0,000 |
| F18 | 0,149 | 0,146 | 0,201 | -0,145 | -0,056 |
| F19 | -0,088 | 0,207 | 0,117 | 0,031 | 0,015 |
| F20 | 0,108 | 0,059 | 0,041 | 0,452 | 0,250 |
| F21 | 0,080 | 0,092 | -0,029 | -0,038 | 0,133 |
| F22 | -0,074 | 0,084 | -0,105 | 0,108 | -0,094 |
| F23 | 0,221 | 0,060 | -0,027 | 0,084 | 0,253 |
| F24 | 0,135 | 0,241 | -0,050 | -0,007 | -0,202 |
| F25 | -0,133 | 0,011 | 0,115 | -0,018 | -0,209 |
| F26 | 0,058 | -0,007 | 0,095 | -0,089 | -0,020 |
| F27 | -0,181 | 0,109 | -0,088 | -0,015 | 0,107 |
| F28 | 0,217 | 0,039 | 0,002 | -0,049 | -0,100 |
| F29 | -0,046 | 0,010 | -0,084 | 0,097 | -0,044 |
| F30 | -0,028 | -0,091 | 0,022 | -0,018 | -0,133 |
| F31 | 0,158 | -0,336 | -0,081 | 0,000 | -0,101 |
| F32 | -0,025 | -0,235 | -0,090 | 0,074 | -0,235 |
| F33 | -0,048 | -0,013 | -0,098 | -0,039 | 0,211 |
| F34 | 0,121 | 0,117 | 0,054 | 0,107 | -0,032 |
| F35 | 0,096 | 0,187 | -0,067 | -0,035 | -0,036 |
| F36 | 0,041 | 0,241 | 0,004 | 0,000 | 0,084 |
| F37 | -0,035 | 0,083 | -0,022 | 0,031 | 0,068 |
| F38 | 0,015 | 0,144 | -0,018 | 0,007 | 0,053 |
| F39 | 0,038 | 0,223 | -0,044 | 0,029 | 0,221 |
| F40 | -0,001 | 0,033 | 0,063 | 0,016 | 0,052 |

**Factor loadings:**

| **desc** | **SS** | | **TIE** | | **M1** | | **SPI** | **GGI1** | | **EEig07x** | | **IC4** | | **ast_violation** | | **TPSA** | | **a_acc** | |  |
| --- | --- | --- | --- | --- | --- | --- | --- | --- | --- | --- | --- | --- | --- | --- | --- | --- | --- | --- | --- | --- |
| F1 | 0,929 | | 0,861 | | 0,827 | | 0,824 | 0,788 | | 0,767 | | 0,720 | | 0,705 | | 0,675 | | 0,587 | |  |
| F2 | -0,160 | | 0,043 | | -0,456 | | -0,067 | -0,161 | | -0,435 | | -0,327 | | -0,252 | | 0,430 | | 0,132 | |  |
| F3 | 0,003 | | 0,123 | | -0,110 | | 0,122 | 0,103 | | -0,214 | | -0,186 | | -0,054 | | 0,296 | | 0,501 | |  |
| F4 | -0,176 | | -0,380 | | 0,035 | | -0,395 | -0,368 | | 0,096 | | 0,208 | | 0,231 | | 0,164 | | 0,310 | |  |
| F5 | 0,094 | | -0,040 | | -0,035 | | -0,034 | 0,071 | | 0,027 | | -0,085 | | 0,073 | | 0,310 | | 0,142 | |  |
| F6 | 0,026 | | -0,026 | | -0,085 | | 0,066 | 0,039 | | -0,013 | | -0,129 | | 0,082 | | -0,037 | | 0,073 | |  |
| F7 | -0,126 | | -0,047 | | -0,019 | | 0,081 | 0,016 | | -0,106 | | -0,096 | | 0,128 | | 0,010 | | 0,139 | |  |
| F8 | -0,024 | | -0,028 | | -0,037 | | 0,043 | -0,020 | | 0,014 | | -0,068 | | -0,067 | | 0,224 | | -0,046 | |  |
| F9 | 0,091 | | 0,000 | | 0,070 | | -0,052 | -0,017 | | 0,056 | | -0,058 | | 0,055 | | 0,079 | | 0,261 | |  |
| F10 | -0,065 | | -0,111 | | 0,023 | | 0,039 | 0,078 | | 0,110 | | -0,122 | | 0,050 | | 0,006 | | 0,063 | |  |
| F11 | 0,005 | | 0,055 | | -0,037 | | -0,076 | -0,134 | | 0,029 | | 0,110 | | -0,008 | | -0,044 | | -0,098 | |  |
| F12 | -0,037 | | 0,015 | | -0,115 | | 0,045 | -0,130 | | 0,108 | | 0,078 | | 0,107 | | -0,015 | | 0,087 | |  |
| F13 | -0,014 | | -0,014 | | -0,058 | | -0,007 | 0,154 | | 0,048 | | -0,046 | | 0,192 | | -0,026 | | 0,022 | |  |
| F14 | -0,056 | | 0,011 | | -0,032 | | 0,097 | 0,075 | | 0,049 | | 0,067 | | 0,006 | | 0,012 | | -0,187 | |  |
| F15 | 0,050 | | 0,086 | | -0,014 | | 0,134 | 0,000 | | -0,030 | | -0,084 | | 0,016 | | 0,110 | | 0,141 | |  |
| F16 | -0,023 | | 0,036 | | -0,071 | | -0,001 | -0,004 | | -0,108 | | -0,020 | | -0,008 | | -0,053 | | 0,084 | |  |
| F17 | 0,047 | | 0,039 | | 0,052 | | 0,082 | 0,071 | | 0,067 | | -0,044 | | -0,044 | | 0,044 | | -0,051 | |  |
| F18 | -0,010 | | 0,067 | | 0,093 | | -0,051 | 0,055 | | 0,011 | | -0,019 | | -0,073 | | 0,088 | | 0,111 | |  |
| F19 | -0,004 | | -0,076 | | 0,038 | | -0,023 | 0,096 | | 0,007 | | -0,070 | | 0,348 | | -0,041 | | 0,092 | |  |
| F20 | 0,004 | | -0,062 | | 0,016 | | 0,097 | 0,077 | | 0,087 | | 0,041 | | -0,247 | | 0,056 | | 0,012 | |  |
| F21 | 0,036 | | -0,017 | | 0,060 | | -0,030 | -0,044 | | 0,027 | | 0,148 | | -0,114 | | 0,022 | | 0,139 | |  |
| F22 | -0,004 | | -0,035 | | 0,038 | | -0,040 | 0,005 | | -0,056 | | 0,346 | | 0,077 | | 0,057 | | -0,002 | |  |
| F23 | 0,026 | | 0,017 | | -0,022 | | -0,029 | -0,133 | | -0,064 | | -0,052 | | 0,162 | | 0,039 | | 0,011 | |  |
| F24 | -0,006 | | -0,023 | | -0,008 | | -0,062 | -0,030 | | 0,084 | | 0,009 | | -0,207 | | 0,057 | | 0,022 | |  |
| F25 | 0,030 | | -0,002 | | 0,038 | | -0,083 | 0,010 | | 0,024 | | 0,111 | | -0,099 | | -0,062 | | 0,012 | |  |
| F26 | 0,003 | | 0,066 | | 0,050 | | 0,094 | 0,159 | | -0,073 | | -0,011 | | -0,044 | | -0,027 | | -0,049 | |  |
| F27 | 0,027 | | -0,004 | | 0,052 | | 0,009 | -0,001 | | -0,019 | | -0,176 | | -0,072 | | 0,063 | | 0,108 | |  |
| F28 | -0,065 | | -0,047 | | -0,062 | | 0,046 | 0,162 | | 0,051 | | -0,019 | | 0,017 | | -0,062 | | 0,031 | |  |
| F29 | 0,009 | | 0,009 | | 0,059 | | -0,051 | -0,097 | | 0,027 | | -0,076 | | 0,020 | | -0,035 | | -0,060 | |  |
| F30 | -0,024 | | 0,022 | | -0,001 | | -0,054 | -0,010 | | 0,244 | | -0,061 | | 0,023 | | 0,022 | | -0,026 | |  |
| F31 | 0,006 | | -0,007 | | -0,005 | | -0,040 | -0,022 | | -0,048 | | -0,004 | | -0,025 | | 0,026 | | 0,119 | |  |
| F32 | -0,016 | | 0,091 | | -0,020 | | 0,126 | -0,125 | | 0,040 | | 0,026 | | 0,009 | | 0,060 | | 0,040 | |  |
| F33 | -0,013 | | -0,057 | | -0,039 | | -0,039 | 0,019 | | 0,097 | | 0,015 | | -0,006 | | 0,001 | | 0,041 | |  |
| F34 | 0,085 | | 0,119 | | 0,093 | | -0,074 | -0,031 | | -0,001 | | -0,027 | | -0,008 | | -0,067 | | -0,019 | |  |
| F35 | 0,027 | | -0,052 | | -0,009 | | 0,045 | -0,035 | | 0,000 | | -0,001 | | 0,004 | | 0,092 | | -0,080 | |  |
| F36 | 0,003 | | -0,033 | | -0,012 | | 0,119 | -0,077 | | 0,029 | | 0,004 | | -0,012 | | -0,116 | | 0,019 | |  |
| F37 | -0,059 | | 0,085 | | -0,067 | | -0,019 | 0,011 | | 0,007 | | 0,020 | | -0,007 | | -0,051 | | 0,056 | |  |
| F38 | -0,026 | | 0,009 | | -0,002 | | 0,003 | -0,004 | | -0,006 | | -0,001 | | 0,002 | | 0,033 | | -0,013 | |  |
| F39 | -0,060 | | 0,072 | | -0,038 | | -0,017 | 0,002 | | 0,001 | | 0,006 | | 0,004 | | 0,041 | | -0,045 | |  |
| F40 | 0,079 | | 0,004 | | -0,075 | | -0,011 | 0,005 | | 0,004 | | 0,002 | | 0,000 | | 0,000 | | -0,005 | |  |
| **desc** | | **RB** | | **HB2** | | **SlogP_VSA0** | | | **a_don** | | **S(-OH)** | | **SMR_VSA2** | | **BELe1** | | **Hy** | | **S(-O-)** | |
| F1 | | 0,569 | | 0,533 | | 0,438 | | | 0,390 | | 0,385 | | 0,373 | | 0,366 | | 0,327 | | 0,290 | |
| F2 | | 0,075 | | 0,412 | | 0,436 | | | 0,716 | | 0,623 | | 0,506 | | -0,243 | | 0,748 | | -0,204 | |
| F3 | | 0,212 | | 0,274 | | 0,213 | | | 0,019 | | -0,110 | | -0,155 | | -0,295 | | 0,059 | | 0,353 | |
| F4 | | 0,119 | | 0,065 | | 0,261 | | | 0,236 | | 0,004 | | -0,103 | | 0,063 | | 0,172 | | 0,226 | |
| F5 | | -0,197 | | 0,426 | | -0,248 | | | -0,024 | | -0,366 | | 0,037 | | -0,226 | | 0,115 | | -0,204 | |
| F6 | | 0,110 | | -0,327 | | 0,127 | | | -0,165 | | 0,056 | | -0,377 | | 0,076 | | -0,186 | | 0,617 | |
| F7 | | 0,288 | | -0,024 | | -0,004 | | | -0,004 | | 0,282 | | -0,013 | | -0,292 | | -0,005 | | -0,004 | |
| F8 | | -0,013 | | 0,049 | | 0,004 | | | -0,135 | | 0,051 | | 0,392 | | -0,046 | | 0,007 | | 0,317 | |
| F9 | | -0,229 | | 0,057 | | -0,352 | | | -0,229 | | 0,267 | | -0,163 | | -0,024 | | -0,305 | | -0,098 | |
| F10 | | -0,330 | | 0,082 | | 0,031 | | | 0,010 | | 0,204 | | -0,066 | | 0,585 | | 0,030 | | -0,109 | |
| F11 | | 0,191 | | -0,062 | | -0,258 | | | -0,166 | | 0,195 | | 0,084 | | 0,020 | | -0,236 | | -0,039 | |
| F12 | | 0,132 | | 0,062 | | -0,019 | | | -0,051 | | -0,106 | | -0,050 | | -0,032 | | -0,084 | | -0,016 | |
| F13 | | 0,006 | | -0,053 | | -0,048 | | | 0,118 | | 0,036 | | 0,013 | | 0,116 | | 0,007 | | -0,056 | |
| F14 | | 0,016 | | 0,062 | | -0,198 | | | 0,209 | | -0,070 | | 0,000 | | 0,060 | | 0,120 | | -0,254 | |
| F15 | | 0,033 | | 0,000 | | -0,034 | | | -0,070 | | -0,072 | | -0,142 | | -0,114 | | -0,062 | | -0,007 | |
| F16 | | 0,228 | | 0,123 | | -0,250 | | | 0,000 | | -0,101 | | 0,115 | | 0,130 | | -0,147 | | 0,025 | |
| F17 | | -0,254 | | -0,024 | | -0,084 | | | 0,110 | | -0,030 | | 0,017 | | -0,167 | | 0,043 | | -0,045 | |
| F18 | | -0,190 | | 0,138 | | 0,099 | | | -0,083 | | -0,027 | | -0,139 | | 0,163 | | 0,009 | | 0,104 | |
| F19 | | -0,246 | | -0,108 | | -0,104 | | | 0,088 | | -0,050 | | 0,172 | | -0,209 | | -0,038 | | 0,144 | |
| F20 | | -0,008 | | 0,009 | | 0,093 | | | -0,060 | | 0,036 | | -0,056 | | -0,011 | | 0,015 | | 0,036 | |
| F21 | | 0,013 | | -0,125 | | -0,059 | | | 0,071 | | -0,129 | | 0,297 | | 0,120 | | -0,074 | | 0,052 | |
| F22 | | 0,021 | | 0,099 | | -0,107 | | | 0,025 | | 0,043 | | -0,171 | | -0,069 | | 0,017 | | 0,046 | |
| F23 | | 0,031 | | 0,032 | | 0,045 | | | -0,047 | | -0,042 | | 0,058 | | 0,207 | | 0,032 | | 0,032 | |
| F24 | | -0,083 | | 0,065 | | -0,193 | | | 0,103 | | -0,003 | | -0,026 | | 0,028 | | -0,001 | | 0,125 | |
| F25 | | -0,080 | | -0,025 | | 0,168 | | | -0,075 | | 0,050 | | 0,111 | | -0,123 | | -0,082 | | 0,005 | |
| F26 | | 0,121 | | 0,021 | | -0,021 | | | -0,029 | | -0,010 | | 0,038 | | 0,000 | | -0,035 | | -0,003 | |
| F27 | | 0,071 | | -0,028 | | -0,067 | | | 0,055 | | -0,032 | | -0,034 | | -0,027 | | -0,011 | | 0,047 | |
| F28 | | -0,018 | | 0,070 | | 0,032 | | | -0,042 | | 0,013 | | 0,053 | | 0,032 | | -0,082 | | 0,016 | |
| F29 | | 0,004 | | 0,238 | | 0,030 | | | -0,065 | | 0,016 | | 0,061 | | -0,046 | | -0,081 | | 0,004 | |
| F30 | | 0,075 | | -0,064 | | -0,005 | | | 0,007 | | 0,012 | | -0,019 | | -0,021 | | 0,021 | | -0,031 | |
| F31 | | 0,013 | | -0,052 | | 0,024 | | | 0,031 | | 0,027 | | -0,016 | | -0,002 | | -0,039 | | -0,105 | |
| F32 | | -0,049 | | -0,027 | | 0,023 | | | -0,038 | | -0,003 | | 0,013 | | -0,016 | | -0,049 | | -0,070 | |
| F33 | | 0,055 | | 0,049 | | 0,002 | | | -0,008 | | -0,013 | | 0,010 | | -0,024 | | 0,006 | | -0,004 | |
| F34 | | 0,000 | | -0,009 | | -0,017 | | | 0,021 | | 0,010 | | -0,012 | | -0,004 | | 0,047 | | 0,032 | |
| F35 | | -0,001 | | -0,025 | | -0,021 | | | -0,084 | | -0,052 | | 0,017 | | -0,003 | | 0,063 | | 0,048 | |
| F36 | | -0,009 | | 0,031 | | -0,009 | | | 0,070 | | 0,019 | | -0,011 | | -0,006 | | 0,011 | | 0,057 | |
| F37 | | -0,017 | | 0,004 | | -0,026 | | | -0,065 | | -0,020 | | 0,010 | | -0,004 | | 0,088 | | 0,017 | |
| F38 | | -0,002 | | -0,012 | | -0,041 | | | -0,013 | | 0,117 | | 0,032 | | 0,003 | | 0,017 | | 0,026 | |
| F39 | | -0,012 | | 0,004 | | 0,022 | | | 0,065 | | -0,017 | | -0,012 | | 0,002 | | -0,068 | | 0,039 | |
| F40 | | -0,006 | | 0,003 | | 0,002 | | | 0,007 | | 0,011 | | -0,001 | | 0,002 | | -0,009 | | 0,004 | |

| **desc** | **O-057** | **nROH** | **O-061** | **GCUT_PEOE_2** | **S(>N-)** | **GATS1p** | **GATS1m** | **SaaO** | **PEOE_VSA_FPOS** |
| --- | --- | --- | --- | --- | --- | --- | --- | --- | --- |
| F1 | 0,290 | 0,289 | 0,130 | 0,108 | 0,021 | 0,012 | 0,002 | -0,021 | -0,051 |
| F2 | 0,528 | 0,558 | 0,159 | 0,341 | -0,490 | -0,217 | -0,106 | -0,109 | -0,339 |
| F3 | -0,044 | 0,007 | -0,250 | -0,524 | 0,184 | 0,771 | 0,677 | 0,037 | 0,345 |
| F4 | 0,094 | 0,014 | -0,211 | 0,368 | -0,048 | -0,043 | -0,014 | 0,215 | 0,248 |
| F5 | -0,394 | -0,342 | 0,177 | 0,143 | -0,235 | 0,121 | 0,278 | 0,141 | -0,389 |
| F6 | 0,137 | 0,015 | -0,178 | -0,106 | -0,565 | -0,066 | 0,001 | 0,404 | -0,082 |
| F7 | 0,225 | 0,232 | 0,088 | -0,404 | 0,110 | -0,293 | -0,065 | -0,003 | -0,032 |
| F8 | -0,163 | -0,173 | 0,718 | -0,206 | -0,045 | -0,151 | -0,163 | 0,371 | 0,330 |
| F9 | 0,255 | 0,249 | 0,018 | 0,101 | 0,326 | -0,046 | -0,134 | 0,216 | 0,097 |
| F10 | 0,162 | 0,011 | 0,211 | -0,152 | -0,097 | 0,193 | 0,150 | -0,409 | 0,071 |
| F11 | 0,155 | 0,160 | 0,243 | 0,174 | 0,002 | 0,240 | 0,430 | 0,119 | -0,150 |
| F12 | -0,128 | -0,018 | -0,064 | -0,126 | 0,022 | -0,225 | -0,197 | -0,340 | 0,057 |
| F13 | 0,221 | -0,055 | -0,135 | -0,060 | -0,226 | 0,025 | 0,127 | 0,098 | 0,027 |
| F14 | -0,022 | 0,142 | -0,261 | -0,091 | 0,005 | 0,070 | -0,124 | 0,428 | -0,026 |
| F15 | 0,190 | -0,274 | -0,089 | 0,006 | 0,014 | -0,062 | -0,209 | -0,146 | -0,090 |
| F16 | -0,232 | 0,294 | -0,084 | -0,038 | -0,276 | -0,023 | 0,006 | -0,151 | 0,210 |
| F17 | 0,057 | -0,001 | -0,099 | 0,040 | 0,000 | -0,088 | 0,086 | -0,004 | 0,527 |
| F18 | -0,157 | 0,072 | -0,082 | -0,117 | -0,040 | -0,093 | 0,017 | 0,110 | -0,081 |
| F19 | -0,091 | 0,198 | 0,004 | -0,027 | 0,010 | 0,062 | -0,061 | -0,089 | -0,178 |
| F20 | -0,159 | 0,251 | -0,026 | 0,009 | 0,154 | -0,026 | -0,019 | 0,007 | -0,092 |
| F21 | 0,107 | -0,093 | -0,111 | -0,097 | 0,075 | -0,056 | 0,052 | 0,027 | -0,063 |
| F22 | -0,009 | -0,031 | 0,076 | 0,065 | -0,051 | -0,003 | -0,007 | -0,043 | 0,037 |
| F23 | -0,074 | 0,041 | -0,014 | 0,165 | 0,137 | -0,018 | -0,098 | 0,048 | 0,047 |
| F24 | 0,093 | 0,013 | 0,030 | 0,003 | -0,105 | -0,018 | -0,132 | -0,048 | -0,069 |
| F25 | -0,015 | 0,047 | -0,068 | 0,111 | -0,101 | 0,060 | -0,001 | -0,055 | 0,037 |
| F26 | 0,026 | -0,026 | 0,014 | 0,077 | -0,010 | 0,043 | -0,088 | -0,037 | -0,004 |
| F27 | -0,005 | -0,015 | -0,013 | 0,185 | 0,046 | -0,024 | 0,075 | -0,029 | 0,009 |
| F28 | 0,016 | -0,050 | -0,015 | 0,125 | -0,040 | 0,039 | -0,087 | 0,057 | 0,016 |
| F29 | 0,073 | -0,018 | -0,076 | -0,056 | -0,017 | 0,000 | -0,011 | 0,016 | -0,013 |
| F30 | -0,042 | 0,004 | 0,023 | 0,009 | -0,045 | 0,001 | -0,004 | -0,012 | 0,005 |
| F31 | -0,037 | -0,015 | 0,035 | -0,044 | -0,032 | 0,139 | -0,122 | 0,031 | 0,026 |
| F32 | -0,007 | 0,015 | -0,009 | 0,023 | -0,070 | -0,014 | 0,012 | 0,016 | -0,013 |
| F33 | 0,012 | -0,011 | -0,010 | -0,033 | 0,060 | 0,054 | -0,013 | -0,009 | 0,014 |
| F34 | -0,011 | 0,005 | 0,009 | -0,008 | -0,009 | 0,053 | -0,049 | 0,004 | 0,005 |
| F35 | 0,056 | 0,041 | -0,028 | 0,002 | -0,009 | 0,100 | -0,049 | -0,033 | 0,011 |
| F36 | -0,019 | -0,016 | 0,018 | 0,011 | 0,020 | 0,049 | -0,006 | 0,011 | -0,002 |
| F37 | 0,025 | 0,007 | -0,001 | 0,011 | 0,014 | 0,004 | 0,002 | 0,005 | 0,004 |
| F38 | -0,061 | -0,057 | -0,066 | -0,003 | 0,009 | 0,011 | -0,002 | -0,014 | -0,005 |
| F39 | 0,005 | 0,005 | 0,006 | 0,004 | 0,039 | 0,029 | -0,007 | -0,011 | -0,002 |
| F40 | -0,005 | -0,003 | -0,006 | -0,001 | 0,006 | -0,007 | 0,002 | 0,000 | 0,000 |

| **desc** | **S(-S-)** | **S(=N-)** | **S(-CH2-)** | **GATS1v** | **S(-C=)** | **S(>C<)** | **GCUT_SMR_1** | **S(>CH-)** |
| --- | --- | --- | --- | --- | --- | --- | --- | --- |
| F1 | -0,055 | -0,072 | -0,073 | -0,095 | -0,123 | -0,212 | -0,249 | -0,346 |
| F2 | -0,128 | -0,155 | -0,315 | -0,333 | -0,527 | -0,054 | -0,273 | -0,332 |
| F3 | -0,211 | 0,018 | 0,105 | 0,754 | -0,332 | 0,078 | 0,348 | -0,088 |
| F4 | 0,058 | 0,475 | 0,109 | 0,268 | 0,423 | 0,479 | -0,453 | 0,120 |
| F5 | 0,412 | 0,532 | -0,387 | -0,084 | -0,079 | -0,065 | 0,061 | -0,034 |
| F6 | 0,025 | -0,102 | -0,595 | -0,029 | 0,000 | -0,128 | 0,002 | -0,267 |
| F7 | 0,571 | 0,282 | 0,136 | -0,068 | 0,279 | 0,099 | 0,497 | 0,036 |
| F8 | -0,288 | 0,019 | 0,013 | -0,070 | 0,044 | 0,148 | 0,071 | 0,169 |
| F9 | -0,224 | 0,413 | -0,049 | -0,035 | -0,235 | -0,212 | -0,113 | -0,118 |
| F10 | -0,101 | 0,110 | -0,254 | 0,004 | 0,267 | 0,205 | 0,201 | -0,026 |
| F11 | 0,271 | -0,252 | 0,005 | -0,081 | -0,113 | 0,418 | -0,108 | -0,067 |
| F12 | 0,083 | 0,017 | -0,140 | -0,074 | -0,070 | 0,049 | 0,042 | -0,261 |
| F13 | 0,072 | 0,053 | 0,179 | -0,130 | -0,172 | -0,222 | 0,042 | 0,611 |
| F14 | -0,221 | -0,007 | -0,081 | -0,022 | 0,098 | 0,266 | 0,272 | -0,111 |
| F15 | -0,127 | -0,041 | -0,098 | -0,099 | -0,216 | 0,481 | -0,015 | 0,249 |
| F16 | -0,085 | 0,100 | 0,001 | -0,093 | -0,043 | 0,036 | -0,085 | 0,092 |
| F17 | 0,264 | -0,145 | -0,125 | -0,028 | -0,041 | 0,071 | -0,083 | -0,044 |
| F18 | 0,107 | -0,085 | 0,347 | -0,234 | -0,066 | 0,144 | -0,041 | -0,104 |
| F19 | -0,061 | -0,136 | 0,013 | 0,068 | 0,064 | 0,081 | -0,038 | 0,022 |
| F20 | 0,067 | -0,035 | -0,219 | 0,041 | 0,051 | 0,025 | -0,061 | 0,279 |
| F21 | 0,045 | 0,018 | -0,080 | -0,051 | 0,021 | -0,016 | 0,013 | -0,022 |
| F22 | -0,040 | -0,116 | -0,033 | -0,049 | 0,058 | -0,057 | 0,119 | 0,059 |
| F23 | 0,120 | -0,096 | -0,050 | 0,105 | -0,164 | -0,014 | 0,189 | 0,046 |
| F24 | 0,070 | -0,055 | 0,093 | 0,215 | -0,054 | -0,026 | 0,069 | -0,004 |
| F25 | -0,064 | 0,099 | 0,020 | -0,046 | -0,084 | 0,055 | 0,205 | -0,009 |
| F26 | 0,028 | 0,065 | 0,065 | 0,139 | 0,053 | 0,045 | -0,075 | -0,042 |
| F27 | -0,078 | -0,067 | 0,032 | -0,113 | 0,148 | -0,038 | 0,115 | -0,006 |
| F28 | 0,086 | -0,019 | 0,011 | -0,008 | 0,052 | 0,001 | 0,026 | -0,020 |
| F29 | -0,018 | -0,082 | -0,050 | 0,011 | 0,075 | -0,032 | -0,019 | 0,037 |
| F30 | -0,010 | 0,012 | 0,014 | 0,022 | -0,048 | 0,008 | 0,027 | -0,006 |
| F31 | 0,049 | -0,062 | -0,014 | -0,027 | 0,017 | -0,025 | -0,026 | 0,000 |
| F32 | -0,008 | -0,018 | 0,043 | 0,052 | 0,080 | -0,027 | 0,001 | 0,022 |
| F33 | -0,014 | -0,052 | 0,006 | -0,055 | 0,007 | -0,028 | -0,020 | -0,011 |
| F34 | 0,033 | 0,037 | -0,027 | -0,022 | 0,038 | 0,015 | 0,005 | 0,029 |
| F35 | 0,025 | 0,032 | 0,028 | -0,062 | 0,016 | -0,017 | -0,003 | -0,009 |
| F36 | 0,010 | 0,031 | 0,015 | -0,042 | -0,032 | 0,001 | 0,000 | 0,000 |
| F37 | -0,007 | -0,005 | -0,011 | -0,006 | 0,022 | -0,004 | -0,003 | 0,006 |
| F38 | 0,003 | 0,003 | -0,002 | -0,003 | 0,004 | -0,003 | -0,008 | 0,001 |
| F39 | 0,007 | 0,025 | -0,009 | -0,030 | 0,006 | -0,008 | -0,002 | 0,005 |
| F40 | 0,000 | 0,002 | 0,010 | 0,004 | 0,022 | 0,007 | 0,001 | 0,002 |

| **desc** | **GVWAI-80** | **logS** | **Q'** | **VEA2** |
| --- | --- | --- | --- | --- |
| F1 | -0,407 | -0,485 | -0,754 | -0,812 |
| F2 | 0,186 | 0,425 | 0,366 | 0,447 |
| F3 | 0,143 | 0,556 | 0,035 | 0,098 |
| F4 | 0,101 | -0,043 | -0,084 | -0,082 |
| F5 | 0,015 | -0,186 | 0,022 | 0,028 |
| F6 | 0,049 | -0,238 | 0,075 | 0,122 |
| F7 | -0,208 | -0,091 | 0,049 | 0,029 |
| F8 | 0,184 | 0,006 | -0,047 | 0,004 |
| F9 | 0,066 | 0,085 | 0,097 | 0,049 |
| F10 | 0,048 | -0,073 | 0,101 | 0,066 |
| F11 | 0,155 | 0,003 | -0,130 | -0,055 |
| F12 | 0,615 | 0,180 | -0,232 | -0,104 |
| F13 | 0,389 | -0,038 | -0,057 | -0,022 |
| F14 | 0,106 | -0,034 | -0,059 | -0,023 |
| F15 | -0,167 | 0,002 | 0,011 | 0,045 |
| F16 | -0,209 | -0,011 | -0,003 | 0,034 |
| F17 | 0,003 | -0,073 | 0,069 | 0,015 |
| F18 | 0,066 | 0,010 | 0,151 | 0,035 |
| F19 | 0,040 | -0,022 | 0,053 | 0,016 |
| F20 | 0,067 | 0,043 | 0,016 | -0,022 |
| F21 | -0,004 | 0,100 | 0,158 | 0,024 |
| F22 | 0,002 | 0,098 | 0,175 | 0,044 |
| F23 | -0,011 | -0,075 | 0,058 | 0,056 |
| F24 | -0,012 | -0,064 | -0,114 | -0,031 |
| F25 | 0,031 | -0,110 | -0,020 | -0,022 |
| F26 | 0,163 | -0,124 | 0,215 | 0,071 |
| F27 | 0,056 | -0,015 | -0,009 | -0,046 |
| F28 | -0,054 | 0,202 | -0,063 | 0,033 |
| F29 | 0,007 | -0,014 | 0,065 | -0,093 |
| F30 | -0,028 | 0,056 | 0,137 | -0,039 |
| F31 | 0,006 | -0,027 | 0,024 | -0,087 |
| F32 | 0,004 | 0,008 | -0,013 | 0,093 |
| F33 | 0,000 | -0,102 | -0,040 | 0,171 |
| F34 | 0,020 | 0,070 | -0,036 | 0,092 |
| F35 | -0,002 | 0,024 | 0,005 | -0,023 |
| F36 | 0,001 | -0,012 | 0,035 | -0,019 |
| F37 | -0,001 | -0,023 | 0,011 | -0,042 |
| F38 | 0,000 | -0,008 | -0,007 | 0,001 |
| F39 | -0,006 | -0,012 | 0,002 | -0,007 |
| F40 | 0,000 | 0,000 | 0,013 | -0,006 |

**Correlations between variables and factors:**

|  | | **Hy** | **S(-OH)** | | **SS** | **HB2** | | **M1** | | **Q'** | | **a_don** | | **ast_violation** | | **SlogP_VSA0** | | |  |
| --- | --- | --- | --- | --- | --- | --- | --- | --- | --- | --- | --- | --- | --- | --- | --- | --- | --- | --- | --- |
| F1 | | 0,327 | 0,385 | | 0,929 | 0,533 | | 0,827 | | -0,754 | | 0,390 | | 0,705 | | 0,438 | | |  |
| F2 | | 0,748 | 0,623 | | -0,160 | 0,412 | | -0,456 | | 0,366 | | 0,716 | | -0,252 | | 0,436 | | |  |
| F3 | | 0,059 | -0,110 | | 0,003 | 0,274 | | -0,110 | | 0,035 | | 0,019 | | -0,054 | | 0,213 | | |  |
| F4 | | 0,172 | 0,004 | | -0,176 | 0,065 | | 0,035 | | -0,084 | | 0,236 | | 0,231 | | 0,261 | | |  |
| F5 | | 0,115 | -0,366 | | 0,094 | 0,426 | | -0,035 | | 0,022 | | -0,024 | | 0,073 | | -0,248 | | |  |
| F6 | | -0,186 | 0,056 | | 0,026 | -0,327 | | -0,085 | | 0,075 | | -0,165 | | 0,082 | | 0,127 | | |  |
| F7 | | -0,005 | 0,282 | | -0,126 | -0,024 | | -0,019 | | 0,049 | | -0,004 | | 0,128 | | -0,004 | | |  |
| F8 | | 0,007 | 0,051 | | -0,024 | 0,049 | | -0,037 | | -0,047 | | -0,135 | | -0,067 | | 0,004 | | |  |
| F9 | | -0,305 | 0,267 | | 0,091 | 0,057 | | 0,070 | | 0,097 | | -0,229 | | 0,055 | | -0,352 | | |  |
| F10 | | 0,030 | 0,204 | | -0,065 | 0,082 | | 0,023 | | 0,101 | | 0,010 | | 0,050 | | 0,031 | | |  |
| F11 | | -0,236 | 0,195 | | 0,005 | -0,062 | | -0,037 | | -0,130 | | -0,166 | | -0,008 | | -0,258 | | |  |
| F12 | | -0,084 | -0,106 | | -0,037 | 0,062 | | -0,115 | | -0,232 | | -0,051 | | 0,107 | | -0,019 | | |  |
| F13 | | 0,007 | 0,036 | | -0,014 | -0,053 | | -0,058 | | -0,057 | | 0,118 | | 0,192 | | -0,048 | | |  |
| F14 | | 0,120 | -0,070 | | -0,056 | 0,062 | | -0,032 | | -0,059 | | 0,209 | | 0,006 | | -0,198 | | |  |
| F15 | | -0,062 | -0,072 | | 0,050 | 0,000 | | -0,014 | | 0,011 | | -0,070 | | 0,016 | | -0,034 | | |  |
| F16 | | -0,147 | -0,101 | | -0,023 | 0,123 | | -0,071 | | -0,003 | | 0,000 | | -0,008 | | -0,250 | | |  |
| F17 | | 0,043 | -0,030 | | 0,047 | -0,024 | | 0,052 | | 0,069 | | 0,110 | | -0,044 | | -0,084 | | |  |
| F18 | | 0,009 | -0,027 | | -0,010 | 0,138 | | 0,093 | | 0,151 | | -0,083 | | -0,073 | | 0,099 | | |  |
| F19 | | -0,038 | -0,050 | | -0,004 | -0,108 | | 0,038 | | 0,053 | | 0,088 | | 0,348 | | -0,104 | | |  |
| F20 | | 0,015 | 0,036 | | 0,004 | 0,009 | | 0,016 | | 0,016 | | -0,060 | | -0,247 | | 0,093 | | |  |
| F21 | | -0,074 | -0,129 | | 0,036 | -0,125 | | 0,060 | | 0,158 | | 0,071 | | -0,114 | | -0,059 | | |  |
| F22 | | 0,017 | 0,043 | | -0,004 | 0,099 | | 0,038 | | 0,175 | | 0,025 | | 0,077 | | -0,107 | | |  |
| F23 | | 0,032 | -0,042 | | 0,026 | 0,032 | | -0,022 | | 0,058 | | -0,047 | | 0,162 | | 0,045 | | |  |
| F24 | | -0,001 | -0,003 | | -0,006 | 0,065 | | -0,008 | | -0,114 | | 0,103 | | -0,207 | | -0,193 | | |  |
| F25 | | -0,082 | 0,050 | | 0,030 | -0,025 | | 0,038 | | -0,020 | | -0,075 | | -0,099 | | 0,168 | | |  |
| F26 | | -0,035 | -0,010 | | 0,003 | 0,021 | | 0,050 | | 0,215 | | -0,029 | | -0,044 | | -0,021 | | |  |
| F27 | | -0,011 | -0,032 | | 0,027 | -0,028 | | 0,052 | | -0,009 | | 0,055 | | -0,072 | | -0,067 | | |  |
| F28 | | -0,082 | 0,013 | | -0,065 | 0,070 | | -0,062 | | -0,063 | | -0,042 | | 0,017 | | 0,032 | | |  |
| F29 | | -0,081 | 0,016 | | 0,009 | 0,238 | | 0,059 | | 0,065 | | -0,065 | | 0,020 | | 0,030 | | |  |
| F30 | | 0,021 | 0,012 | | -0,024 | -0,064 | | -0,001 | | 0,137 | | 0,007 | | 0,023 | | -0,005 | | |  |
| F31 | | -0,039 | 0,027 | | 0,006 | -0,052 | | -0,005 | | 0,024 | | 0,031 | | -0,025 | | 0,024 | | |  |
| F32 | | -0,049 | -0,003 | | -0,016 | -0,027 | | -0,020 | | -0,013 | | -0,038 | | 0,009 | | 0,023 | | |  |
| F33 | | 0,006 | -0,013 | | -0,013 | 0,049 | | -0,039 | | -0,040 | | -0,008 | | -0,006 | | 0,002 | | |  |
| F34 | | 0,047 | 0,010 | | 0,085 | -0,009 | | 0,093 | | -0,036 | | 0,021 | | -0,008 | | -0,017 | | |  |
| F35 | | 0,063 | -0,052 | | 0,027 | -0,025 | | -0,009 | | 0,005 | | -0,084 | | 0,004 | | -0,021 | | |  |
| F36 | | 0,011 | 0,019 | | 0,003 | 0,031 | | -0,012 | | 0,035 | | 0,070 | | -0,012 | | -0,009 | | |  |
| F37 | | 0,088 | -0,020 | | -0,059 | 0,004 | | -0,067 | | 0,011 | | -0,065 | | -0,007 | | -0,026 | | |  |
| F38 | | 0,017 | 0,117 | | -0,026 | -0,012 | | -0,002 | | -0,007 | | -0,013 | | 0,002 | | -0,041 | | |  |
| F39 | | -0,068 | -0,017 | | -0,060 | 0,004 | | -0,038 | | 0,002 | | 0,065 | | 0,004 | | 0,022 | | |  |
| F40 | | -0,009 | 0,011 | | 0,079 | 0,003 | | -0,075 | | 0,013 | | 0,007 | | 0,000 | | 0,002 | | |  |
|  | **GCUT_PEOE_2** | | | **SMR_VSA2** | | | **a_acc** | | **GCUT_SMR_1** | | **logS** | | **RB** | | **O-061** | | **nROH** | **O-057** | |
| F1 | 0,108 | | | 0,373 | | | 0,587 | | -0,249 | | -0,485 | | 0,569 | | 0,130 | | 0,289 | 0,290 | |
| F2 | 0,341 | | | 0,506 | | | 0,132 | | -0,273 | | 0,425 | | 0,075 | | 0,159 | | 0,558 | 0,528 | |
| F3 | -0,524 | | | -0,155 | | | 0,501 | | 0,348 | | 0,556 | | 0,212 | | -0,250 | | 0,007 | -0,044 | |
| F4 | 0,368 | | | -0,103 | | | 0,310 | | -0,453 | | -0,043 | | 0,119 | | -0,211 | | 0,014 | 0,094 | |
| F5 | 0,143 | | | 0,037 | | | 0,142 | | 0,061 | | -0,186 | | -0,197 | | 0,177 | | -0,342 | -0,394 | |
| F6 | -0,106 | | | -0,377 | | | 0,073 | | 0,002 | | -0,238 | | 0,110 | | -0,178 | | 0,015 | 0,137 | |
| F7 | -0,404 | | | -0,013 | | | 0,139 | | 0,497 | | -0,091 | | 0,288 | | 0,088 | | 0,232 | 0,225 | |
| F8 | -0,206 | | | 0,392 | | | -0,046 | | 0,071 | | 0,006 | | -0,013 | | 0,718 | | -0,173 | -0,163 | |
| F9 | 0,101 | | | -0,163 | | | 0,261 | | -0,113 | | 0,085 | | -0,229 | | 0,018 | | 0,249 | 0,255 | |
| F10 | -0,152 | | | -0,066 | | | 0,063 | | 0,201 | | -0,073 | | -0,330 | | 0,211 | | 0,011 | 0,162 | |
| F11 | 0,174 | | | 0,084 | | | -0,098 | | -0,108 | | 0,003 | | 0,191 | | 0,243 | | 0,160 | 0,155 | |
| F12 | -0,126 | | | -0,050 | | | 0,087 | | 0,042 | | 0,180 | | 0,132 | | -0,064 | | -0,018 | -0,128 | |
| F13 | -0,060 | | | 0,013 | | | 0,022 | | 0,042 | | -0,038 | | 0,006 | | -0,135 | | -0,055 | 0,221 | |
| F14 | -0,091 | | | 0,000 | | | -0,187 | | 0,272 | | -0,034 | | 0,016 | | -0,261 | | 0,142 | -0,022 | |
| F15 | 0,006 | | | -0,142 | | | 0,141 | | -0,015 | | 0,002 | | 0,033 | | -0,089 | | -0,274 | 0,190 | |
| F16 | -0,038 | | | 0,115 | | | 0,084 | | -0,085 | | -0,011 | | 0,228 | | -0,084 | | 0,294 | -0,232 | |
| F17 | 0,040 | | | 0,017 | | | -0,051 | | -0,083 | | -0,073 | | -0,254 | | -0,099 | | -0,001 | 0,057 | |
| F18 | -0,117 | | | -0,139 | | | 0,111 | | -0,041 | | 0,010 | | -0,190 | | -0,082 | | 0,072 | -0,157 | |
| F19 | -0,027 | | | 0,172 | | | 0,092 | | -0,038 | | -0,022 | | -0,246 | | 0,004 | | 0,198 | -0,091 | |
| F20 | 0,009 | | | -0,056 | | | 0,012 | | -0,061 | | 0,043 | | -0,008 | | -0,026 | | 0,251 | -0,159 | |
| F21 | -0,097 | | | 0,297 | | | 0,139 | | 0,013 | | 0,100 | | 0,013 | | -0,111 | | -0,093 | 0,107 | |
| F22 | 0,065 | | | -0,171 | | | -0,002 | | 0,119 | | 0,098 | | 0,021 | | 0,076 | | -0,031 | -0,009 | |
| F23 | 0,165 | | | 0,058 | | | 0,011 | | 0,189 | | -0,075 | | 0,031 | | -0,014 | | 0,041 | -0,074 | |
| F24 | 0,003 | | | -0,026 | | | 0,022 | | 0,069 | | -0,064 | | -0,083 | | 0,030 | | 0,013 | 0,093 | |
| F25 | 0,111 | | | 0,111 | | | 0,012 | | 0,205 | | -0,110 | | -0,080 | | -0,068 | | 0,047 | -0,015 | |
| F26 | 0,077 | | | 0,038 | | | -0,049 | | -0,075 | | -0,124 | | 0,121 | | 0,014 | | -0,026 | 0,026 | |
| F27 | 0,185 | | | -0,034 | | | 0,108 | | 0,115 | | -0,015 | | 0,071 | | -0,013 | | -0,015 | -0,005 | |
| F28 | 0,125 | | | 0,053 | | | 0,031 | | 0,026 | | 0,202 | | -0,018 | | -0,015 | | -0,050 | 0,016 | |
| F29 | -0,056 | | | 0,061 | | | -0,060 | | -0,019 | | -0,014 | | 0,004 | | -0,076 | | -0,018 | 0,073 | |
| F30 | 0,009 | | | -0,019 | | | -0,026 | | 0,027 | | 0,056 | | 0,075 | | 0,023 | | 0,004 | -0,042 | |
| F31 | -0,044 | | | -0,016 | | | 0,119 | | -0,026 | | -0,027 | | 0,013 | | 0,035 | | -0,015 | -0,037 | |
| F32 | 0,023 | | | 0,013 | | | 0,040 | | 0,001 | | 0,008 | | -0,049 | | -0,009 | | 0,015 | -0,007 | |
| F33 | -0,033 | | | 0,010 | | | 0,041 | | -0,020 | | -0,102 | | 0,055 | | -0,010 | | -0,011 | 0,012 | |
| F34 | -0,008 | | | -0,012 | | | -0,019 | | 0,005 | | 0,070 | | 0,000 | | 0,009 | | 0,005 | -0,011 | |
| F35 | 0,002 | | | 0,017 | | | -0,080 | | -0,003 | | 0,024 | | -0,001 | | -0,028 | | 0,041 | 0,056 | |
| F36 | 0,011 | | | -0,011 | | | 0,019 | | 0,000 | | -0,012 | | -0,009 | | 0,018 | | -0,016 | -0,019 | |
| F37 | 0,011 | | | 0,010 | | | 0,056 | | -0,003 | | -0,023 | | -0,017 | | -0,001 | | 0,007 | 0,025 | |
| F38 | -0,003 | | | 0,032 | | | -0,013 | | -0,008 | | -0,008 | | -0,002 | | -0,066 | | -0,057 | -0,061 | |
| F39 | 0,004 | | | -0,012 | | | -0,045 | | -0,002 | | -0,012 | | -0,012 | | 0,006 | | 0,005 | 0,005 | |
| F40 | -0,001 | | | -0,001 | | | -0,005 | | 0,001 | | 0,000 | | -0,006 | | -0,006 | | -0,003 | -0,005 | |

|  | **SPI** | **EEig07x** | **GGI1** | **GATS1v** | **GATS1p** | **GATS1m** | **SaaO** | **BELe1** | **IC4** |
| --- | --- | --- | --- | --- | --- | --- | --- | --- | --- |
| F1 | 0,824 | 0,767 | 0,788 | -0,095 | 0,012 | 0,002 | -0,021 | 0,366 | 0,720 |
| F2 | -0,067 | -0,435 | -0,161 | -0,333 | -0,217 | -0,106 | -0,109 | -0,243 | -0,327 |
| F3 | 0,122 | -0,214 | 0,103 | 0,754 | 0,771 | 0,677 | 0,037 | -0,295 | -0,186 |
| F4 | -0,395 | 0,096 | -0,368 | 0,268 | -0,043 | -0,014 | 0,215 | 0,063 | 0,208 |
| F5 | -0,034 | 0,027 | 0,071 | -0,084 | 0,121 | 0,278 | 0,141 | -0,226 | -0,085 |
| F6 | 0,066 | -0,013 | 0,039 | -0,029 | -0,066 | 0,001 | 0,404 | 0,076 | -0,129 |
| F7 | 0,081 | -0,106 | 0,016 | -0,068 | -0,293 | -0,065 | -0,003 | -0,292 | -0,096 |
| F8 | 0,043 | 0,014 | -0,020 | -0,070 | -0,151 | -0,163 | 0,371 | -0,046 | -0,068 |
| F9 | -0,052 | 0,056 | -0,017 | -0,035 | -0,046 | -0,134 | 0,216 | -0,024 | -0,058 |
| F10 | 0,039 | 0,110 | 0,078 | 0,004 | 0,193 | 0,150 | -0,409 | 0,585 | -0,122 |
| F11 | -0,076 | 0,029 | -0,134 | -0,081 | 0,240 | 0,430 | 0,119 | 0,020 | 0,110 |
| F12 | 0,045 | 0,108 | -0,130 | -0,074 | -0,225 | -0,197 | -0,340 | -0,032 | 0,078 |
| F13 | -0,007 | 0,048 | 0,154 | -0,130 | 0,025 | 0,127 | 0,098 | 0,116 | -0,046 |
| F14 | 0,097 | 0,049 | 0,075 | -0,022 | 0,070 | -0,124 | 0,428 | 0,060 | 0,067 |
| F15 | 0,134 | -0,030 | 0,000 | -0,099 | -0,062 | -0,209 | -0,146 | -0,114 | -0,084 |
| F16 | -0,001 | -0,108 | -0,004 | -0,093 | -0,023 | 0,006 | -0,151 | 0,130 | -0,020 |
| F17 | 0,082 | 0,067 | 0,071 | -0,028 | -0,088 | 0,086 | -0,004 | -0,167 | -0,044 |
| F18 | -0,051 | 0,011 | 0,055 | -0,234 | -0,093 | 0,017 | 0,110 | 0,163 | -0,019 |
| F19 | -0,023 | 0,007 | 0,096 | 0,068 | 0,062 | -0,061 | -0,089 | -0,209 | -0,070 |
| F20 | 0,097 | 0,087 | 0,077 | 0,041 | -0,026 | -0,019 | 0,007 | -0,011 | 0,041 |
| F21 | -0,030 | 0,027 | -0,044 | -0,051 | -0,056 | 0,052 | 0,027 | 0,120 | 0,148 |
| F22 | -0,040 | -0,056 | 0,005 | -0,049 | -0,003 | -0,007 | -0,043 | -0,069 | 0,346 |
| F23 | -0,029 | -0,064 | -0,133 | 0,105 | -0,018 | -0,098 | 0,048 | 0,207 | -0,052 |
| F24 | -0,062 | 0,084 | -0,030 | 0,215 | -0,018 | -0,132 | -0,048 | 0,028 | 0,009 |
| F25 | -0,083 | 0,024 | 0,010 | -0,046 | 0,060 | -0,001 | -0,055 | -0,123 | 0,111 |
| F26 | 0,094 | -0,073 | 0,159 | 0,139 | 0,043 | -0,088 | -0,037 | 0,000 | -0,011 |
| F27 | 0,009 | -0,019 | -0,001 | -0,113 | -0,024 | 0,075 | -0,029 | -0,027 | -0,176 |
| F28 | 0,046 | 0,051 | 0,162 | -0,008 | 0,039 | -0,087 | 0,057 | 0,032 | -0,019 |
| F29 | -0,051 | 0,027 | -0,097 | 0,011 | 0,000 | -0,011 | 0,016 | -0,046 | -0,076 |
| F30 | -0,054 | 0,244 | -0,010 | 0,022 | 0,001 | -0,004 | -0,012 | -0,021 | -0,061 |
| F31 | -0,040 | -0,048 | -0,022 | -0,027 | 0,139 | -0,122 | 0,031 | -0,002 | -0,004 |
| F32 | 0,126 | 0,040 | -0,125 | 0,052 | -0,014 | 0,012 | 0,016 | -0,016 | 0,026 |
| F33 | -0,039 | 0,097 | 0,019 | -0,055 | 0,054 | -0,013 | -0,009 | -0,024 | 0,015 |
| F34 | -0,074 | -0,001 | -0,031 | -0,022 | 0,053 | -0,049 | 0,004 | -0,004 | -0,027 |
| F35 | 0,045 | 0,000 | -0,035 | -0,062 | 0,100 | -0,049 | -0,033 | -0,003 | -0,001 |
| F36 | 0,119 | 0,029 | -0,077 | -0,042 | 0,049 | -0,006 | 0,011 | -0,006 | 0,004 |
| F37 | -0,019 | 0,007 | 0,011 | -0,006 | 0,004 | 0,002 | 0,005 | -0,004 | 0,020 |
| F38 | 0,003 | -0,006 | -0,004 | -0,003 | 0,011 | -0,002 | -0,014 | 0,003 | -0,001 |
| F39 | -0,017 | 0,001 | 0,002 | -0,030 | 0,029 | -0,007 | -0,011 | 0,002 | 0,006 |
| F40 | -0,011 | 0,004 | 0,005 | 0,004 | -0,007 | 0,002 | 0,000 | 0,002 | 0,002 |

|  | **TIE** | **VEA2** | **GVWAI-80** | **TPSA** | **PEOE_VSA_FPOS** | **S(-CH2-)** | **S(-C=)** | **S(=N-)** |
| --- | --- | --- | --- | --- | --- | --- | --- | --- |
| F1 | 0,861 | -0,812 | -0,407 | 0,675 | -0,051 | -0,073 | -0,123 | -0,072 |
| F2 | 0,043 | 0,447 | 0,186 | 0,430 | -0,339 | -0,315 | -0,527 | -0,155 |
| F3 | 0,123 | 0,098 | 0,143 | 0,296 | 0,345 | 0,105 | -0,332 | 0,018 |
| F4 | -0,380 | -0,082 | 0,101 | 0,164 | 0,248 | 0,109 | 0,423 | 0,475 |
| F5 | -0,040 | 0,028 | 0,015 | 0,310 | -0,389 | -0,387 | -0,079 | 0,532 |
| F6 | -0,026 | 0,122 | 0,049 | -0,037 | -0,082 | -0,595 | 0,000 | -0,102 |
| F7 | -0,047 | 0,029 | -0,208 | 0,010 | -0,032 | 0,136 | 0,279 | 0,282 |
| F8 | -0,028 | 0,004 | 0,184 | 0,224 | 0,330 | 0,013 | 0,044 | 0,019 |
| F9 | 0,000 | 0,049 | 0,066 | 0,079 | 0,097 | -0,049 | -0,235 | 0,413 |
| F10 | -0,111 | 0,066 | 0,048 | 0,006 | 0,071 | -0,254 | 0,267 | 0,110 |
| F11 | 0,055 | -0,055 | 0,155 | -0,044 | -0,150 | 0,005 | -0,113 | -0,252 |
| F12 | 0,015 | -0,104 | 0,615 | -0,015 | 0,057 | -0,140 | -0,070 | 0,017 |
| F13 | -0,014 | -0,022 | 0,389 | -0,026 | 0,027 | 0,179 | -0,172 | 0,053 |
| F14 | 0,011 | -0,023 | 0,106 | 0,012 | -0,026 | -0,081 | 0,098 | -0,007 |
| F15 | 0,086 | 0,045 | -0,167 | 0,110 | -0,090 | -0,098 | -0,216 | -0,041 |
| F16 | 0,036 | 0,034 | -0,209 | -0,053 | 0,210 | 0,001 | -0,043 | 0,100 |
| F17 | 0,039 | 0,015 | 0,003 | 0,044 | 0,527 | -0,125 | -0,041 | -0,145 |
| F18 | 0,067 | 0,035 | 0,066 | 0,088 | -0,081 | 0,347 | -0,066 | -0,085 |
| F19 | -0,076 | 0,016 | 0,040 | -0,041 | -0,178 | 0,013 | 0,064 | -0,136 |
| F20 | -0,062 | -0,022 | 0,067 | 0,056 | -0,092 | -0,219 | 0,051 | -0,035 |
| F21 | -0,017 | 0,024 | -0,004 | 0,022 | -0,063 | -0,080 | 0,021 | 0,018 |
| F22 | -0,035 | 0,044 | 0,002 | 0,057 | 0,037 | -0,033 | 0,058 | -0,116 |
| F23 | 0,017 | 0,056 | -0,011 | 0,039 | 0,047 | -0,050 | -0,164 | -0,096 |
| F24 | -0,023 | -0,031 | -0,012 | 0,057 | -0,069 | 0,093 | -0,054 | -0,055 |
| F25 | -0,002 | -0,022 | 0,031 | -0,062 | 0,037 | 0,020 | -0,084 | 0,099 |
| F26 | 0,066 | 0,071 | 0,163 | -0,027 | -0,004 | 0,065 | 0,053 | 0,065 |
| F27 | -0,004 | -0,046 | 0,056 | 0,063 | 0,009 | 0,032 | 0,148 | -0,067 |
| F28 | -0,047 | 0,033 | -0,054 | -0,062 | 0,016 | 0,011 | 0,052 | -0,019 |
| F29 | 0,009 | -0,093 | 0,007 | -0,035 | -0,013 | -0,050 | 0,075 | -0,082 |
| F30 | 0,022 | -0,039 | -0,028 | 0,022 | 0,005 | 0,014 | -0,048 | 0,012 |
| F31 | -0,007 | -0,087 | 0,006 | 0,026 | 0,026 | -0,014 | 0,017 | -0,062 |
| F32 | 0,091 | 0,093 | 0,004 | 0,060 | -0,013 | 0,043 | 0,080 | -0,018 |
| F33 | -0,057 | 0,171 | 0,000 | 0,001 | 0,014 | 0,006 | 0,007 | -0,052 |
| F34 | 0,119 | 0,092 | 0,020 | -0,067 | 0,005 | -0,027 | 0,038 | 0,037 |
| F35 | -0,052 | -0,023 | -0,002 | 0,092 | 0,011 | 0,028 | 0,016 | 0,032 |
| F36 | -0,033 | -0,019 | 0,001 | -0,116 | -0,002 | 0,015 | -0,032 | 0,031 |
| F37 | 0,085 | -0,042 | -0,001 | -0,051 | 0,004 | -0,011 | 0,022 | -0,005 |
| F38 | 0,009 | 0,001 | 0,000 | 0,033 | -0,005 | -0,002 | 0,004 | 0,003 |
| F39 | 0,072 | -0,007 | -0,006 | 0,041 | -0,002 | -0,009 | 0,006 | 0,025 |
| F40 | 0,004 | -0,006 | 0,000 | 0,000 | 0,000 | 0,010 | 0,022 | 0,002 |

|  | **S(-S-)** | **S(-O-)** | **S(>C<)** | **S(>CH-)** | **S(>N-)** |
| --- | --- | --- | --- | --- | --- |
| F1 | -0,055 | 0,290 | -0,212 | -0,346 | 0,021 |
| F2 | -0,128 | -0,204 | -0,054 | -0,332 | -0,490 |
| F3 | -0,211 | 0,353 | 0,078 | -0,088 | 0,184 |
| F4 | 0,058 | 0,226 | 0,479 | 0,120 | -0,048 |
| F5 | 0,412 | -0,204 | -0,065 | -0,034 | -0,235 |
| F6 | 0,025 | 0,617 | -0,128 | -0,267 | -0,565 |
| F7 | 0,571 | -0,004 | 0,099 | 0,036 | 0,110 |
| F8 | -0,288 | 0,317 | 0,148 | 0,169 | -0,045 |
| F9 | -0,224 | -0,098 | -0,212 | -0,118 | 0,326 |
| F10 | -0,101 | -0,109 | 0,205 | -0,026 | -0,097 |
| F11 | 0,271 | -0,039 | 0,418 | -0,067 | 0,002 |
| F12 | 0,083 | -0,016 | 0,049 | -0,261 | 0,022 |
| F13 | 0,072 | -0,056 | -0,222 | 0,611 | -0,226 |
| F14 | -0,221 | -0,254 | 0,266 | -0,111 | 0,005 |
| F15 | -0,127 | -0,007 | 0,481 | 0,249 | 0,014 |
| F16 | -0,085 | 0,025 | 0,036 | 0,092 | -0,276 |
| F17 | 0,264 | -0,045 | 0,071 | -0,044 | 0,000 |
| F18 | 0,107 | 0,104 | 0,144 | -0,104 | -0,040 |
| F19 | -0,061 | 0,144 | 0,081 | 0,022 | 0,010 |
| F20 | 0,067 | 0,036 | 0,025 | 0,279 | 0,154 |
| F21 | 0,045 | 0,052 | -0,016 | -0,022 | 0,075 |
| F22 | -0,040 | 0,046 | -0,057 | 0,059 | -0,051 |
| F23 | 0,120 | 0,032 | -0,014 | 0,046 | 0,137 |
| F24 | 0,070 | 0,125 | -0,026 | -0,004 | -0,105 |
| F25 | -0,064 | 0,005 | 0,055 | -0,009 | -0,101 |
| F26 | 0,028 | -0,003 | 0,045 | -0,042 | -0,010 |
| F27 | -0,078 | 0,047 | -0,038 | -0,006 | 0,046 |
| F28 | 0,086 | 0,016 | 0,001 | -0,020 | -0,040 |
| F29 | -0,018 | 0,004 | -0,032 | 0,037 | -0,017 |
| F30 | -0,010 | -0,031 | 0,008 | -0,006 | -0,045 |
| F31 | 0,049 | -0,105 | -0,025 | 0,000 | -0,032 |
| F32 | -0,008 | -0,070 | -0,027 | 0,022 | -0,070 |
| F33 | -0,014 | -0,004 | -0,028 | -0,011 | 0,060 |
| F34 | 0,033 | 0,032 | 0,015 | 0,029 | -0,009 |
| F35 | 0,025 | 0,048 | -0,017 | -0,009 | -0,009 |
| F36 | 0,010 | 0,057 | 0,001 | 0,000 | 0,020 |
| F37 | -0,007 | 0,017 | -0,004 | 0,006 | 0,014 |
| F38 | 0,003 | 0,026 | -0,003 | 0,001 | 0,009 |
| F39 | 0,007 | 0,039 | -0,008 | 0,005 | 0,039 |
| F40 | 0,000 | 0,004 | 0,007 | 0,002 | 0,006 |

**Correlations of the variables (%):**

|  | | **Hy** | **S(-OH)** | | **SS** | **HB2** | | **M1** | | **Q'** | | **a_don** | | **ast_violation** | | **SlogP_VSA0** | | |  |
| --- | --- | --- | --- | --- | --- | --- | --- | --- | --- | --- | --- | --- | --- | --- | --- | --- | --- | --- | --- |
| F1 | | 1,109 | 1,540 | | 8,952 | 2,946 | | 7,083 | | 5,897 | | 1,572 | | 5,146 | | 1,984 | | |  |
| F2 | | 10,308 | 7,144 | | 0,471 | 3,120 | | 3,833 | | 2,461 | | 9,448 | | 1,166 | | 3,503 | | |  |
| F3 | | 0,095 | 0,331 | | 0,000 | 2,051 | | 0,333 | | 0,034 | | 0,010 | | 0,080 | | 1,243 | | |  |
| F4 | | 1,339 | 0,001 | | 1,403 | 0,190 | | 0,057 | | 0,317 | | 2,521 | | 2,432 | | 3,083 | | |  |
| F5 | | 0,668 | 6,793 | | 0,452 | 9,173 | | 0,062 | | 0,025 | | 0,030 | | 0,267 | | 3,112 | | |  |
| F6 | | 1,865 | 0,167 | | 0,036 | 5,747 | | 0,383 | | 0,299 | | 1,458 | | 0,358 | | 0,862 | | |  |
| F7 | | 0,002 | 5,203 | | 1,036 | 0,036 | | 0,024 | | 0,159 | | 0,001 | | 1,067 | | 0,001 | | |  |
| F8 | | 0,003 | 0,184 | | 0,041 | 0,166 | | 0,095 | | 0,155 | | 1,257 | | 0,309 | | 0,001 | | |  |
| F9 | | 7,583 | 5,803 | | 0,675 | 0,264 | | 0,401 | | 0,764 | | 4,287 | | 0,246 | | 10,114 | | |  |
| F10 | | 0,075 | 3,551 | | 0,362 | 0,572 | | 0,045 | | 0,865 | | 0,009 | | 0,215 | | 0,084 | | |  |
| F11 | | 5,070 | 3,446 | | 0,002 | 0,345 | | 0,124 | | 1,540 | | 2,511 | | 0,006 | | 6,037 | | |  |
| F12 | | 0,738 | 1,184 | | 0,145 | 0,404 | | 1,401 | | 5,676 | | 0,273 | | 1,209 | | 0,038 | | |  |
| F13 | | 0,005 | 0,144 | | 0,020 | 0,308 | | 0,358 | | 0,347 | | 1,508 | | 3,968 | | 0,249 | | |  |
| F14 | | 1,872 | 0,626 | | 0,406 | 0,503 | | 0,132 | | 0,449 | | 5,624 | | 0,005 | | 5,058 | | |  |
| F15 | | 0,529 | 0,716 | | 0,348 | 0,000 | | 0,026 | | 0,018 | | 0,675 | | 0,037 | | 0,159 | | |  |
| F16 | | 3,603 | 1,681 | | 0,089 | 2,494 | | 0,833 | | 0,001 | | 0,000 | | 0,010 | | 10,342 | | |  |
| F17 | | 0,320 | 0,150 | | 0,383 | 0,096 | | 0,453 | | 0,799 | | 2,061 | | 0,325 | | 1,193 | | |  |
| F18 | | 0,017 | 0,137 | | 0,018 | 3,690 | | 1,699 | | 4,441 | | 1,335 | | 1,024 | | 1,925 | | |  |
| F19 | | 0,294 | 0,527 | | 0,003 | 2,423 | | 0,303 | | 0,574 | | 1,617 | | 25,138 | | 2,233 | | |  |
| F20 | | 0,059 | 0,340 | | 0,003 | 0,023 | | 0,070 | | 0,071 | | 0,950 | | 16,041 | | 2,278 | | |  |
| F21 | | 1,723 | 5,169 | | 0,414 | 4,864 | | 1,136 | | 7,750 | | 1,568 | | 4,047 | | 1,088 | | |  |
| F22 | | 0,101 | 0,606 | | 0,005 | 3,268 | | 0,491 | | 10,250 | | 0,210 | | 2,004 | | 3,844 | | |  |
| F23 | | 0,342 | 0,602 | | 0,239 | 0,345 | | 0,172 | | 1,146 | | 0,762 | | 8,913 | | 0,687 | | |  |
| F24 | | 0,001 | 0,004 | | 0,015 | 1,546 | | 0,026 | | 4,801 | | 3,955 | | 15,852 | | 13,788 | | |  |
| F25 | | 2,848 | 1,050 | | 0,377 | 0,275 | | 0,611 | | 0,177 | | 2,395 | | 4,210 | | 12,073 | | |  |
| F26 | | 0,546 | 0,041 | | 0,005 | 0,201 | | 1,140 | | 20,703 | | 0,384 | | 0,852 | | 0,194 | | |  |
| F27 | | 0,061 | 0,548 | | 0,384 | 0,438 | | 1,459 | | 0,047 | | 1,610 | | 2,832 | | 2,427 | | |  |
| F28 | | 4,237 | 0,111 | | 2,651 | 3,066 | | 2,425 | | 2,483 | | 1,095 | | 0,188 | | 0,651 | | |  |
| F29 | | 4,455 | 0,166 | | 0,049 | 38,060 | | 2,333 | | 2,873 | | 2,863 | | 0,264 | | 0,605 | | |  |
| F30 | | 0,386 | 0,134 | | 0,509 | 3,548 | | 0,002 | | 16,459 | | 0,037 | | 0,467 | | 0,019 | | |  |
| F31 | | 1,585 | 0,746 | | 0,042 | 2,724 | | 0,025 | | 0,570 | | 0,976 | | 0,631 | | 0,610 | | |  |
| F32 | | 2,738 | 0,008 | | 0,274 | 0,833 | | 0,472 | | 0,184 | | 1,610 | | 0,100 | | 0,601 | | |  |
| F33 | | 0,047 | 0,214 | | 0,223 | 2,901 | | 1,877 | | 1,919 | | 0,088 | | 0,047 | | 0,006 | | |  |
| F34 | | 2,998 | 0,148 | | 9,790 | 0,117 | | 11,729 | | 1,793 | | 0,593 | | 0,079 | | 0,383 | | |  |
| F35 | | 5,879 | 4,108 | | 1,131 | 0,976 | | 0,112 | | 0,036 | | 10,688 | | 0,025 | | 0,637 | | |  |
| F36 | | 0,229 | 0,680 | | 0,020 | 1,689 | | 0,246 | | 2,186 | | 8,702 | | 0,257 | | 0,145 | | |  |
| F37 | | 19,406 | 1,006 | | 8,795 | 0,035 | | 11,232 | | 0,288 | | 10,539 | | 0,113 | | 1,703 | | |  |
| F38 | | 0,900 | 43,029 | | 2,126 | 0,444 | | 0,017 | | 0,149 | | 0,551 | | 0,010 | | 5,389 | | |  |
| F39 | | 15,314 | 0,997 | | 11,762 | 0,059 | | 4,838 | | 0,010 | | 13,911 | | 0,061 | | 1,617 | | |  |
| F40 | | 0,652 | 0,965 | | 46,343 | 0,060 | | 41,945 | | 1,284 | | 0,319 | | 0,000 | | 0,031 | | |  |
|  | **GCUT_PEOE_2** | | | **SMR_VSA2** | | | **a_acc** | | **GCUT_SMR_1** | | **logS** | | **RB** | | **O-061** | | **nROH** | **O-057** | |
| F1 | 0,121 | | | 1,442 | | | 3,575 | | 0,642 | | 2,433 | | 3,360 | | 0,175 | | 0,868 | 0,870 | |
| F2 | 2,140 | | | 4,723 | | | 0,321 | | 1,369 | | 3,320 | | 0,104 | | 0,468 | | 5,739 | 5,124 | |
| F3 | 7,515 | | | 0,656 | | | 6,868 | | 3,322 | | 8,469 | | 1,236 | | 1,714 | | 0,001 | 0,053 | |
| F4 | 6,152 | | | 0,482 | | | 4,357 | | 9,325 | | 0,084 | | 0,648 | | 2,021 | | 0,009 | 0,400 | |
| F5 | 1,034 | | | 0,069 | | | 1,025 | | 0,186 | | 1,755 | | 1,964 | | 1,592 | | 5,939 | 7,870 | |
| F6 | 0,606 | | | 7,642 | | | 0,289 | | 0,000 | | 3,051 | | 0,649 | | 1,706 | | 0,012 | 1,009 | |
| F7 | 10,695 | | | 0,011 | | | 1,262 | | 16,146 | | 0,542 | | 5,423 | | 0,505 | | 3,522 | 3,320 | |
| F8 | 2,947 | | | 10,681 | | | 0,148 | | 0,351 | | 0,003 | | 0,013 | | 35,814 | | 2,083 | 1,839 | |
| F9 | 0,826 | | | 2,165 | | | 5,536 | | 1,033 | | 0,587 | | 4,281 | | 0,026 | | 5,061 | 5,287 | |
| F10 | 1,961 | | | 0,367 | | | 0,334 | | 3,426 | | 0,458 | | 9,293 | | 3,781 | | 0,011 | 2,229 | |
| F11 | 2,753 | | | 0,647 | | | 0,875 | | 1,069 | | 0,001 | | 3,335 | | 5,353 | | 2,326 | 2,193 | |
| F12 | 1,688 | | | 0,260 | | | 0,803 | | 0,187 | | 3,410 | | 1,830 | | 0,432 | | 0,035 | 1,736 | |
| F13 | 0,390 | | | 0,018 | | | 0,053 | | 0,187 | | 0,155 | | 0,004 | | 1,975 | | 0,332 | 5,264 | |
| F14 | 1,066 | | | 0,000 | | | 4,512 | | 9,531 | | 0,149 | | 0,031 | | 8,783 | | 2,588 | 0,061 | |
| F15 | 0,005 | | | 2,801 | | | 2,758 | | 0,031 | | 0,001 | | 0,154 | | 1,105 | | 10,386 | 5,007 | |
| F16 | 0,238 | | | 2,215 | | | 1,158 | | 1,194 | | 0,021 | | 8,609 | | 1,177 | | 14,305 | 8,928 | |
| F17 | 0,269 | | | 0,047 | | | 0,441 | | 1,168 | | 0,899 | | 10,922 | | 1,652 | | 0,000 | 0,551 | |
| F18 | 2,663 | | | 3,778 | | | 2,386 | | 0,334 | | 0,020 | | 7,041 | | 1,305 | | 1,010 | 4,822 | |
| F19 | 0,152 | | | 6,098 | | | 1,769 | | 0,297 | | 0,099 | | 12,570 | | 0,004 | | 8,150 | 1,721 | |
| F20 | 0,022 | | | 0,826 | | | 0,039 | | 0,967 | | 0,478 | | 0,018 | | 0,182 | | 16,503 | 6,632 | |
| F21 | 2,894 | | | 27,400 | | | 6,027 | | 0,055 | | 3,118 | | 0,056 | | 3,837 | | 2,699 | 3,536 | |
| F22 | 1,430 | | | 9,831 | | | 0,002 | | 4,707 | | 3,194 | | 0,150 | | 1,912 | | 0,323 | 0,027 | |
| F23 | 9,330 | | | 1,151 | | | 0,038 | | 12,212 | | 1,936 | | 0,324 | | 0,064 | | 0,561 | 1,857 | |
| F24 | 0,002 | | | 0,247 | | | 0,177 | | 1,741 | | 1,513 | | 2,568 | | 0,325 | | 0,061 | 3,188 | |
| F25 | 5,249 | | | 5,225 | | | 0,063 | | 18,022 | | 5,175 | | 2,764 | | 1,963 | | 0,950 | 0,099 | |
| F26 | 2,643 | | | 0,638 | | | 1,063 | | 2,497 | | 6,912 | | 6,560 | | 0,085 | | 0,302 | 0,297 | |
| F27 | 18,631 | | | 0,612 | | | 6,258 | | 7,220 | | 0,117 | | 2,696 | | 0,097 | | 0,116 | 0,013 | |
| F28 | 9,831 | | | 1,796 | | | 0,613 | | 0,419 | | 25,713 | | 0,208 | | 0,144 | | 1,602 | 0,167 | |
| F29 | 2,081 | | | 2,540 | | | 2,405 | | 0,233 | | 0,140 | | 0,013 | | 3,901 | | 0,214 | 3,635 | |
| F30 | 0,071 | | | 0,308 | | | 0,605 | | 0,619 | | 2,785 | | 4,933 | | 0,461 | | 0,017 | 1,524 | |
| F31 | 1,994 | | | 0,251 | | | 14,468 | | 0,718 | | 0,746 | | 0,161 | | 1,261 | | 0,246 | 1,413 | |
| F32 | 0,574 | | | 0,194 | | | 1,769 | | 0,000 | | 0,071 | | 2,708 | | 0,092 | | 0,253 | 0,056 | |
| F33 | 1,357 | | | 0,115 | | | 2,023 | | 0,512 | | 12,774 | | 3,689 | | 0,120 | | 0,140 | 0,178 | |
| F34 | 0,083 | | | 0,202 | | | 0,488 | | 0,031 | | 6,673 | | 0,000 | | 0,113 | | 0,037 | 0,167 | |
| F35 | 0,007 | | | 0,421 | | | 9,520 | | 0,011 | | 0,877 | | 0,002 | | 1,173 | | 2,530 | 4,686 | |
| F36 | 0,201 | | | 0,224 | | | 0,643 | | 0,000 | | 0,259 | | 0,137 | | 0,601 | | 0,453 | 0,618 | |
| F37 | 0,293 | | | 0,269 | | | 7,802 | | 0,017 | | 1,376 | | 0,748 | | 0,002 | | 0,123 | 1,602 | |
| F38 | 0,027 | | | 3,144 | | | 0,562 | | 0,203 | | 0,179 | | 0,008 | | 13,691 | | 10,328 | 11,737 | |
| F39 | 0,042 | | | 0,489 | | | 6,766 | | 0,016 | | 0,508 | | 0,498 | | 0,117 | | 0,086 | 0,073 | |
| F40 | 0,017 | | | 0,016 | | | 0,198 | | 0,004 | | 0,001 | | 0,292 | | 0,273 | | 0,076 | 0,213 | |

|  | **SPI** | **EEig07x** | **GGI1** | **GATS1v** | **GATS1p** | **GATS1m** | **SaaO** | **BELe1** | **IC4** |
| --- | --- | --- | --- | --- | --- | --- | --- | --- | --- |
| F1 | 7,045 | 6,095 | 6,431 | 0,094 | 0,001 | 0,000 | 0,005 | 1,387 | 5,376 |
| F2 | 0,082 | 3,483 | 0,475 | 2,046 | 0,865 | 0,208 | 0,217 | 1,086 | 1,963 |
| F3 | 0,409 | 1,253 | 0,292 | 15,593 | 16,311 | 12,565 | 0,038 | 2,386 | 0,953 |
| F4 | 7,077 | 0,418 | 6,151 | 3,269 | 0,084 | 0,009 | 2,106 | 0,182 | 1,962 |
| F5 | 0,059 | 0,036 | 0,258 | 0,356 | 0,740 | 3,915 | 1,008 | 2,579 | 0,365 |
| F6 | 0,232 | 0,009 | 0,083 | 0,045 | 0,230 | 0,000 | 8,777 | 0,310 | 0,898 |
| F7 | 0,435 | 0,733 | 0,017 | 0,303 | 5,609 | 0,276 | 0,001 | 5,585 | 0,598 |
| F8 | 0,128 | 0,013 | 0,027 | 0,344 | 1,583 | 1,850 | 9,531 | 0,149 | 0,322 |
| F9 | 0,224 | 0,256 | 0,024 | 0,102 | 0,175 | 1,463 | 3,801 | 0,045 | 0,277 |
| F10 | 0,129 | 1,033 | 0,514 | 0,001 | 3,160 | 1,928 | 14,238 | 29,116 | 1,260 |
| F11 | 0,530 | 0,075 | 1,642 | 0,592 | 5,254 | 16,820 | 1,291 | 0,036 | 1,099 |
| F12 | 0,217 | 1,233 | 1,772 | 0,579 | 5,324 | 4,084 | 12,232 | 0,107 | 0,642 |
| F13 | 0,005 | 0,247 | 2,572 | 1,819 | 0,069 | 1,731 | 1,030 | 1,443 | 0,233 |
| F14 | 1,212 | 0,308 | 0,735 | 0,061 | 0,640 | 1,989 | 23,588 | 0,458 | 0,587 |
| F15 | 2,487 | 0,123 | 0,000 | 1,349 | 0,542 | 6,042 | 2,944 | 1,806 | 0,978 |
| F16 | 0,000 | 1,952 | 0,003 | 1,437 | 0,091 | 0,005 | 3,785 | 2,800 | 0,068 |
| F17 | 1,134 | 0,762 | 0,863 | 0,129 | 1,321 | 1,266 | 0,002 | 4,711 | 0,326 |
| F18 | 0,515 | 0,023 | 0,582 | 10,645 | 1,697 | 0,058 | 2,336 | 5,152 | 0,069 |
| F19 | 0,107 | 0,010 | 1,906 | 0,966 | 0,805 | 0,769 | 1,659 | 9,077 | 1,003 |
| F20 | 2,467 | 1,989 | 1,554 | 0,440 | 0,172 | 0,097 | 0,012 | 0,033 | 0,437 |
| F21 | 0,288 | 0,222 | 0,594 | 0,799 | 0,973 | 0,851 | 0,221 | 4,491 | 6,793 |
| F22 | 0,547 | 1,053 | 0,007 | 0,798 | 0,002 | 0,015 | 0,620 | 1,574 | 40,059 |
| F23 | 0,289 | 1,383 | 6,055 | 3,753 | 0,112 | 3,265 | 0,800 | 14,648 | 0,934 |
| F24 | 1,410 | 2,634 | 0,337 | 17,144 | 0,114 | 6,430 | 0,862 | 0,280 | 0,030 |
| F25 | 2,912 | 0,254 | 0,043 | 0,921 | 1,553 | 0,001 | 1,307 | 6,506 | 5,306 |
| F26 | 3,987 | 2,389 | 11,385 | 8,683 | 0,819 | 3,489 | 0,619 | 0,000 | 0,051 |
| F27 | 0,044 | 0,186 | 0,000 | 6,967 | 0,314 | 3,051 | 0,449 | 0,401 | 16,715 |
| F28 | 1,331 | 1,615 | 16,444 | 0,043 | 0,976 | 4,715 | 2,014 | 0,657 | 0,236 |
| F29 | 1,777 | 0,500 | 6,294 | 0,089 | 0,000 | 0,087 | 0,173 | 1,413 | 3,885 |
| F30 | 2,608 | 52,149 | 0,087 | 0,425 | 0,001 | 0,014 | 0,117 | 0,403 | 3,310 |
| F31 | 1,661 | 2,338 | 0,506 | 0,746 | 19,947 | 15,402 | 0,977 | 0,005 | 0,020 |
| F32 | 17,898 | 1,843 | 17,512 | 3,004 | 0,230 | 0,158 | 0,294 | 0,283 | 0,759 |
| F33 | 1,888 | 11,564 | 0,423 | 3,677 | 3,600 | 0,212 | 0,108 | 0,679 | 0,278 |
| F34 | 7,518 | 0,000 | 1,287 | 0,654 | 3,784 | 3,312 | 0,020 | 0,018 | 1,004 |
| F35 | 3,040 | 0,000 | 1,810 | 5,842 | 15,164 | 3,653 | 1,603 | 0,011 | 0,002 |
| F36 | 25,471 | 1,469 | 10,793 | 3,152 | 4,249 | 0,066 | 0,200 | 0,063 | 0,024 |
| F37 | 0,904 | 0,128 | 0,292 | 0,084 | 0,041 | 0,009 | 0,054 | 0,050 | 1,009 |
| F38 | 0,033 | 0,121 | 0,044 | 0,030 | 0,382 | 0,009 | 0,570 | 0,024 | 0,001 |
| F39 | 0,951 | 0,002 | 0,019 | 2,904 | 2,697 | 0,162 | 0,389 | 0,015 | 0,124 |
| F40 | 0,950 | 0,100 | 0,169 | 0,116 | 0,368 | 0,027 | 0,000 | 0,030 | 0,044 |

|  | **TIE** | **VEA2** | **GVWAI-80** | **TPSA** | **PEOE_VSA_FPOS** | **S(-CH2-)** | **S(-C=)** | **S(=N-)** |
| --- | --- | --- | --- | --- | --- | --- | --- | --- |
| F1 | 7,680 | 6,826 | 1,719 | 4,725 | 0,027 | 0,055 | 0,156 | 0,054 |
| F2 | 0,034 | 3,672 | 0,636 | 3,403 | 2,115 | 1,830 | 5,115 | 0,440 |
| F3 | 0,418 | 0,261 | 0,564 | 2,407 | 3,261 | 0,303 | 3,027 | 0,009 |
| F4 | 6,568 | 0,306 | 0,460 | 1,228 | 2,787 | 0,540 | 8,141 | 10,246 |
| F5 | 0,081 | 0,039 | 0,012 | 4,855 | 7,674 | 7,587 | 0,312 | 14,361 |
| F6 | 0,035 | 0,794 | 0,128 | 0,072 | 0,364 | 18,989 | 0,000 | 0,557 |
| F7 | 0,142 | 0,056 | 2,845 | 0,007 | 0,066 | 1,207 | 5,110 | 5,210 |
| F8 | 0,053 | 0,001 | 2,357 | 3,476 | 7,538 | 0,012 | 0,135 | 0,025 |
| F9 | 0,000 | 0,192 | 0,355 | 0,504 | 0,771 | 0,195 | 4,490 | 13,865 |
| F10 | 1,053 | 0,368 | 0,197 | 0,003 | 0,433 | 5,485 | 6,092 | 1,029 |
| F11 | 0,279 | 0,271 | 2,184 | 0,173 | 2,037 | 0,002 | 1,155 | 5,798 |
| F12 | 0,023 | 1,152 | 39,965 | 0,022 | 0,344 | 2,063 | 0,516 | 0,030 |
| F13 | 0,021 | 0,053 | 16,343 | 0,070 | 0,080 | 3,448 | 3,183 | 0,307 |
| F14 | 0,015 | 0,069 | 1,450 | 0,020 | 0,086 | 0,850 | 1,251 | 0,006 |
| F15 | 1,029 | 0,282 | 3,888 | 1,689 | 1,122 | 1,328 | 6,475 | 0,239 |
| F16 | 0,212 | 0,191 | 7,227 | 0,475 | 7,322 | 0,000 | 0,306 | 1,647 |
| F17 | 0,253 | 0,037 | 0,002 | 0,333 | 47,230 | 2,657 | 0,288 | 3,571 |
| F18 | 0,878 | 0,245 | 0,853 | 1,499 | 1,277 | 23,478 | 0,845 | 1,392 |
| F19 | 1,187 | 0,052 | 0,334 | 0,346 | 6,559 | 0,033 | 0,858 | 3,820 |
| F20 | 1,015 | 0,133 | 1,165 | 0,818 | 2,225 | 12,592 | 0,695 | 0,315 |
| F21 | 0,091 | 0,184 | 0,004 | 0,146 | 1,249 | 2,003 | 0,136 | 0,097 |
| F22 | 0,418 | 0,638 | 0,001 | 1,082 | 0,451 | 0,374 | 1,125 | 4,493 |
| F23 | 0,102 | 1,063 | 0,041 | 0,511 | 0,769 | 0,870 | 9,154 | 3,127 |
| F24 | 0,188 | 0,355 | 0,051 | 1,189 | 1,758 | 3,225 | 1,095 | 1,110 |
| F25 | 0,002 | 0,199 | 0,405 | 1,654 | 0,593 | 0,164 | 3,015 | 4,163 |
| F26 | 1,955 | 2,228 | 11,849 | 0,325 | 0,008 | 1,915 | 1,242 | 1,901 |
| F27 | 0,011 | 1,134 | 1,668 | 2,151 | 0,041 | 0,550 | 11,936 | 2,427 |
| F28 | 1,405 | 0,703 | 1,846 | 2,383 | 0,161 | 0,072 | 1,684 | 0,227 |
| F29 | 0,060 | 5,806 | 0,036 | 0,843 | 0,112 | 1,660 | 3,818 | 4,558 |
| F30 | 0,433 | 1,350 | 0,709 | 0,437 | 0,019 | 0,162 | 2,005 | 0,130 |
| F31 | 0,051 | 7,861 | 0,039 | 0,673 | 0,715 | 0,206 | 0,293 | 3,912 |
| F32 | 9,361 | 9,768 | 0,018 | 4,013 | 0,201 | 2,041 | 7,200 | 0,361 |
| F33 | 4,040 | 35,821 | 0,000 | 0,001 | 0,254 | 0,042 | 0,057 | 3,330 |
| F34 | 19,500 | 11,622 | 0,521 | 6,077 | 0,032 | 1,033 | 2,024 | 1,821 |
| F35 | 4,014 | 0,799 | 0,004 | 12,821 | 0,182 | 1,217 | 0,383 | 1,510 |
| F36 | 1,927 | 0,637 | 0,002 | 24,060 | 0,009 | 0,421 | 1,798 | 1,696 |
| F37 | 18,167 | 4,403 | 0,002 | 6,408 | 0,050 | 0,308 | 1,250 | 0,068 |
| F38 | 0,234 | 0,003 | 0,000 | 3,488 | 0,067 | 0,016 | 0,047 | 0,023 |
| F39 | 16,932 | 0,175 | 0,121 | 5,611 | 0,009 | 0,257 | 0,101 | 2,095 |
| F40 | 0,135 | 0,252 | 0,000 | 0,001 | 0,002 | 0,811 | 3,485 | 0,032 |

|  | **S(-S-)** | **S(-O-)** | **S(>C<)** | **S(>CH-)** | **S(>N-)** |
| --- | --- | --- | --- | --- | --- |
| F1 | 0,031 | 0,870 | 0,464 | 1,240 | 0,004 |
| F2 | 0,304 | 0,763 | 0,054 | 2,032 | 4,418 |
| F3 | 1,219 | 3,414 | 0,167 | 0,213 | 0,925 |
| F4 | 0,150 | 2,312 | 10,424 | 0,656 | 0,105 |
| F5 | 8,581 | 2,104 | 0,215 | 0,057 | 2,790 |
| F6 | 0,033 | 20,448 | 0,879 | 3,839 | 17,139 |
| F7 | 21,331 | 0,001 | 0,638 | 0,084 | 0,794 |
| F8 | 5,770 | 6,973 | 1,511 | 1,974 | 0,139 |
| F9 | 4,094 | 0,787 | 3,675 | 1,130 | 8,633 |
| F10 | 0,871 | 1,008 | 3,594 | 0,056 | 0,796 |
| F11 | 6,681 | 0,139 | 15,897 | 0,413 | 0,000 |
| F12 | 0,719 | 0,025 | 0,250 | 7,199 | 0,053 |
| F13 | 0,566 | 0,338 | 5,324 | 40,323 | 5,509 |
| F14 | 6,286 | 8,297 | 9,111 | 1,580 | 0,003 |
| F15 | 2,238 | 0,008 | 32,041 | 8,613 | 0,025 |
| F16 | 1,207 | 0,104 | 0,215 | 1,417 | 12,637 |
| F17 | 11,857 | 0,348 | 0,854 | 0,327 | 0,000 |
| F18 | 2,233 | 2,119 | 4,053 | 2,094 | 0,313 |
| F19 | 0,767 | 4,279 | 1,372 | 0,096 | 0,022 |
| F20 | 1,175 | 0,347 | 0,164 | 20,387 | 6,264 |
| F21 | 0,635 | 0,845 | 0,084 | 0,148 | 1,766 |
| F22 | 0,545 | 0,700 | 1,101 | 1,162 | 0,880 |
| F23 | 4,891 | 0,357 | 0,070 | 0,712 | 6,412 |
| F24 | 1,827 | 5,815 | 0,254 | 0,005 | 4,076 |
| F25 | 1,770 | 0,013 | 1,315 | 0,031 | 4,348 |
| F26 | 0,340 | 0,005 | 0,912 | 0,794 | 0,042 |
| F27 | 3,269 | 1,188 | 0,766 | 0,021 | 1,146 |
| F28 | 4,696 | 0,155 | 0,001 | 0,244 | 0,994 |
| F29 | 0,211 | 0,011 | 0,699 | 0,944 | 0,196 |
| F30 | 0,081 | 0,837 | 0,050 | 0,034 | 1,757 |
| F31 | 2,505 | 11,297 | 0,658 | 0,000 | 1,019 |
| F32 | 0,064 | 5,543 | 0,818 | 0,547 | 5,545 |
| F33 | 0,229 | 0,017 | 0,959 | 0,154 | 4,438 |
| F34 | 1,453 | 1,368 | 0,292 | 1,136 | 0,103 |
| F35 | 0,912 | 3,515 | 0,451 | 0,124 | 0,126 |
| F36 | 0,168 | 5,802 | 0,002 | 0,000 | 0,699 |
| F37 | 0,126 | 0,694 | 0,048 | 0,098 | 0,460 |
| F38 | 0,022 | 2,074 | 0,032 | 0,004 | 0,281 |
| F39 | 0,145 | 4,970 | 0,195 | 0,086 | 4,875 |
| F40 | 0,000 | 0,111 | 0,392 | 0,026 | 0,265 |

**Squared cosines of the variables:**

|  | | **Hy** | **S(-OH)** | | **SS** | **HB2** | | **M1** | | **Q'** | | **a_don** | | **ast_violation** | | **SlogP_VSA0** | | |  |
| --- | --- | --- | --- | --- | --- | --- | --- | --- | --- | --- | --- | --- | --- | --- | --- | --- | --- | --- | --- |
| F1 | | 0,107 | 0,149 | | 0,864 | 0,284 | | 0,683 | | 0,569 | | 0,152 | | 0,497 | | 0,191 | | |  |
| F2 | | 0,560 | 0,388 | | 0,026 | 0,169 | | 0,208 | | 0,134 | | 0,513 | | 0,063 | | 0,190 | | |  |
| F3 | | 0,003 | 0,012 | | 0,000 | 0,075 | | 0,012 | | 0,001 | | 0,000 | | 0,003 | | 0,045 | | |  |
| F4 | | 0,030 | 0,000 | | 0,031 | 0,004 | | 0,001 | | 0,007 | | 0,056 | | 0,054 | | 0,068 | | |  |
| F5 | | 0,013 | 0,134 | | 0,009 | 0,181 | | 0,001 | | 0,001 | | 0,001 | | 0,005 | | 0,061 | | |  |
| F6 | | 0,035 | 0,003 | | 0,001 | 0,107 | | 0,007 | | 0,006 | | 0,027 | | 0,007 | | 0,016 | | |  |
| F7 | | 0,000 | 0,079 | | 0,016 | 0,001 | | 0,000 | | 0,002 | | 0,000 | | 0,016 | | 0,000 | | |  |
| F8 | | 0,000 | 0,003 | | 0,001 | 0,002 | | 0,001 | | 0,002 | | 0,018 | | 0,004 | | 0,000 | | |  |
| F9 | | 0,093 | 0,071 | | 0,008 | 0,003 | | 0,005 | | 0,009 | | 0,053 | | 0,003 | | 0,124 | | |  |
| F10 | | 0,001 | 0,042 | | 0,004 | 0,007 | | 0,001 | | 0,010 | | 0,000 | | 0,003 | | 0,001 | | |  |
| F11 | | 0,056 | 0,038 | | 0,000 | 0,004 | | 0,001 | | 0,017 | | 0,028 | | 0,000 | | 0,066 | | |  |
| F12 | | 0,007 | 0,011 | | 0,001 | 0,004 | | 0,013 | | 0,054 | | 0,003 | | 0,011 | | 0,000 | | |  |
| F13 | | 0,000 | 0,001 | | 0,000 | 0,003 | | 0,003 | | 0,003 | | 0,014 | | 0,037 | | 0,002 | | |  |
| F14 | | 0,015 | 0,005 | | 0,003 | 0,004 | | 0,001 | | 0,003 | | 0,044 | | 0,000 | | 0,039 | | |  |
| F15 | | 0,004 | 0,005 | | 0,003 | 0,000 | | 0,000 | | 0,000 | | 0,005 | | 0,000 | | 0,001 | | |  |
| F16 | | 0,022 | 0,010 | | 0,001 | 0,015 | | 0,005 | | 0,000 | | 0,000 | | 0,000 | | 0,062 | | |  |
| F17 | | 0,002 | 0,001 | | 0,002 | 0,001 | | 0,003 | | 0,005 | | 0,012 | | 0,002 | | 0,007 | | |  |
| F18 | | 0,000 | 0,001 | | 0,000 | 0,019 | | 0,009 | | 0,023 | | 0,007 | | 0,005 | | 0,010 | | |  |
| F19 | | 0,001 | 0,003 | | 0,000 | 0,012 | | 0,001 | | 0,003 | | 0,008 | | 0,121 | | 0,011 | | |  |
| F20 | | 0,000 | 0,001 | | 0,000 | 0,000 | | 0,000 | | 0,000 | | 0,004 | | 0,061 | | 0,009 | | |  |
| F21 | | 0,006 | 0,017 | | 0,001 | 0,016 | | 0,004 | | 0,025 | | 0,005 | | 0,013 | | 0,004 | | |  |
| F22 | | 0,000 | 0,002 | | 0,000 | 0,010 | | 0,001 | | 0,031 | | 0,001 | | 0,006 | | 0,011 | | |  |
| F23 | | 0,001 | 0,002 | | 0,001 | 0,001 | | 0,001 | | 0,003 | | 0,002 | | 0,026 | | 0,002 | | |  |
| F24 | | 0,000 | 0,000 | | 0,000 | 0,004 | | 0,000 | | 0,013 | | 0,011 | | 0,043 | | 0,037 | | |  |
| F25 | | 0,007 | 0,002 | | 0,001 | 0,001 | | 0,001 | | 0,000 | | 0,006 | | 0,010 | | 0,028 | | |  |
| F26 | | 0,001 | 0,000 | | 0,000 | 0,000 | | 0,003 | | 0,046 | | 0,001 | | 0,002 | | 0,000 | | |  |
| F27 | | 0,000 | 0,001 | | 0,001 | 0,001 | | 0,003 | | 0,000 | | 0,003 | | 0,005 | | 0,004 | | |  |
| F28 | | 0,007 | 0,000 | | 0,004 | 0,005 | | 0,004 | | 0,004 | | 0,002 | | 0,000 | | 0,001 | | |  |
| F29 | | 0,007 | 0,000 | | 0,000 | 0,057 | | 0,003 | | 0,004 | | 0,004 | | 0,000 | | 0,001 | | |  |
| F30 | | 0,000 | 0,000 | | 0,001 | 0,004 | | 0,000 | | 0,019 | | 0,000 | | 0,001 | | 0,000 | | |  |
| F31 | | 0,002 | 0,001 | | 0,000 | 0,003 | | 0,000 | | 0,001 | | 0,001 | | 0,001 | | 0,001 | | |  |
| F32 | | 0,002 | 0,000 | | 0,000 | 0,001 | | 0,000 | | 0,000 | | 0,001 | | 0,000 | | 0,001 | | |  |
| F33 | | 0,000 | 0,000 | | 0,000 | 0,002 | | 0,002 | | 0,002 | | 0,000 | | 0,000 | | 0,000 | | |  |
| F34 | | 0,002 | 0,000 | | 0,007 | 0,000 | | 0,009 | | 0,001 | | 0,000 | | 0,000 | | 0,000 | | |  |
| F35 | | 0,004 | 0,003 | | 0,001 | 0,001 | | 0,000 | | 0,000 | | 0,007 | | 0,000 | | 0,000 | | |  |
| F36 | | 0,000 | 0,000 | | 0,000 | 0,001 | | 0,000 | | 0,001 | | 0,005 | | 0,000 | | 0,000 | | |  |
| F37 | | 0,008 | 0,000 | | 0,004 | 0,000 | | 0,005 | | 0,000 | | 0,004 | | 0,000 | | 0,001 | | |  |
| F38 | | 0,000 | 0,014 | | 0,001 | 0,000 | | 0,000 | | 0,000 | | 0,000 | | 0,000 | | 0,002 | | |  |
| F39 | | 0,005 | 0,000 | | 0,004 | 0,000 | | 0,001 | | 0,000 | | 0,004 | | 0,000 | | 0,000 | | |  |
| F40 | | 0,000 | 0,000 | | 0,006 | 0,000 | | 0,006 | | 0,000 | | 0,000 | | 0,000 | | 0,000 | | |  |
|  | **GCUT_PEOE_2** | | | **SMR_VSA2** | | | **a_acc** | | **GCUT_SMR_1** | | **logS** | | **RB** | | **O-061** | | **nROH** | **O-057** | |
| F1 | 0,012 | | | 0,139 | | | 0,345 | | 0,062 | | 0,235 | | 0,324 | | 0,017 | | 0,084 | 0,084 | |
| F2 | 0,116 | | | 0,257 | | | 0,017 | | 0,074 | | 0,180 | | 0,006 | | 0,025 | | 0,312 | 0,278 | |
| F3 | 0,274 | | | 0,024 | | | 0,251 | | 0,121 | | 0,309 | | 0,045 | | 0,063 | | 0,000 | 0,002 | |
| F4 | 0,136 | | | 0,011 | | | 0,096 | | 0,205 | | 0,002 | | 0,014 | | 0,045 | | 0,000 | 0,009 | |
| F5 | 0,020 | | | 0,001 | | | 0,020 | | 0,004 | | 0,035 | | 0,039 | | 0,031 | | 0,117 | 0,155 | |
| F6 | 0,011 | | | 0,142 | | | 0,005 | | 0,000 | | 0,057 | | 0,012 | | 0,032 | | 0,000 | 0,019 | |
| F7 | 0,163 | | | 0,000 | | | 0,019 | | 0,247 | | 0,008 | | 0,083 | | 0,008 | | 0,054 | 0,051 | |
| F8 | 0,042 | | | 0,154 | | | 0,002 | | 0,005 | | 0,000 | | 0,000 | | 0,516 | | 0,030 | 0,026 | |
| F9 | 0,010 | | | 0,027 | | | 0,068 | | 0,013 | | 0,007 | | 0,053 | | 0,000 | | 0,062 | 0,065 | |
| F10 | 0,023 | | | 0,004 | | | 0,004 | | 0,040 | | 0,005 | | 0,109 | | 0,044 | | 0,000 | 0,026 | |
| F11 | 0,030 | | | 0,007 | | | 0,010 | | 0,012 | | 0,000 | | 0,037 | | 0,059 | | 0,026 | 0,024 | |
| F12 | 0,016 | | | 0,002 | | | 0,008 | | 0,002 | | 0,032 | | 0,017 | | 0,004 | | 0,000 | 0,016 | |
| F13 | 0,004 | | | 0,000 | | | 0,000 | | 0,002 | | 0,001 | | 0,000 | | 0,018 | | 0,003 | 0,049 | |
| F14 | 0,008 | | | 0,000 | | | 0,035 | | 0,074 | | 0,001 | | 0,000 | | 0,068 | | 0,020 | 0,000 | |
| F15 | 0,000 | | | 0,020 | | | 0,020 | | 0,000 | | 0,000 | | 0,001 | | 0,008 | | 0,075 | 0,036 | |
| F16 | 0,001 | | | 0,013 | | | 0,007 | | 0,007 | | 0,000 | | 0,052 | | 0,007 | | 0,086 | 0,054 | |
| F17 | 0,002 | | | 0,000 | | | 0,003 | | 0,007 | | 0,005 | | 0,064 | | 0,010 | | 0,000 | 0,003 | |
| F18 | 0,014 | | | 0,019 | | | 0,012 | | 0,002 | | 0,000 | | 0,036 | | 0,007 | | 0,005 | 0,025 | |
| F19 | 0,001 | | | 0,029 | | | 0,009 | | 0,001 | | 0,000 | | 0,061 | | 0,000 | | 0,039 | 0,008 | |
| F20 | 0,000 | | | 0,003 | | | 0,000 | | 0,004 | | 0,002 | | 0,000 | | 0,001 | | 0,063 | 0,025 | |
| F21 | 0,009 | | | 0,088 | | | 0,019 | | 0,000 | | 0,010 | | 0,000 | | 0,012 | | 0,009 | 0,011 | |
| F22 | 0,004 | | | 0,029 | | | 0,000 | | 0,014 | | 0,010 | | 0,000 | | 0,006 | | 0,001 | 0,000 | |
| F23 | 0,027 | | | 0,003 | | | 0,000 | | 0,036 | | 0,006 | | 0,001 | | 0,000 | | 0,002 | 0,005 | |
| F24 | 0,000 | | | 0,001 | | | 0,000 | | 0,005 | | 0,004 | | 0,007 | | 0,001 | | 0,000 | 0,009 | |
| F25 | 0,012 | | | 0,012 | | | 0,000 | | 0,042 | | 0,012 | | 0,006 | | 0,005 | | 0,002 | 0,000 | |
| F26 | 0,006 | | | 0,001 | | | 0,002 | | 0,006 | | 0,015 | | 0,015 | | 0,000 | | 0,001 | 0,001 | |
| F27 | 0,034 | | | 0,001 | | | 0,012 | | 0,013 | | 0,000 | | 0,005 | | 0,000 | | 0,000 | 0,000 | |
| F28 | 0,016 | | | 0,003 | | | 0,001 | | 0,001 | | 0,041 | | 0,000 | | 0,000 | | 0,003 | 0,000 | |
| F29 | 0,003 | | | 0,004 | | | 0,004 | | 0,000 | | 0,000 | | 0,000 | | 0,006 | | 0,000 | 0,005 | |
| F30 | 0,000 | | | 0,000 | | | 0,001 | | 0,001 | | 0,003 | | 0,006 | | 0,001 | | 0,000 | 0,002 | |
| F31 | 0,002 | | | 0,000 | | | 0,014 | | 0,001 | | 0,001 | | 0,000 | | 0,001 | | 0,000 | 0,001 | |
| F32 | 0,001 | | | 0,000 | | | 0,002 | | 0,000 | | 0,000 | | 0,002 | | 0,000 | | 0,000 | 0,000 | |
| F33 | 0,001 | | | 0,000 | | | 0,002 | | 0,000 | | 0,010 | | 0,003 | | 0,000 | | 0,000 | 0,000 | |
| F34 | 0,000 | | | 0,000 | | | 0,000 | | 0,000 | | 0,005 | | 0,000 | | 0,000 | | 0,000 | 0,000 | |
| F35 | 0,000 | | | 0,000 | | | 0,006 | | 0,000 | | 0,001 | | 0,000 | | 0,001 | | 0,002 | 0,003 | |
| F36 | 0,000 | | | 0,000 | | | 0,000 | | 0,000 | | 0,000 | | 0,000 | | 0,000 | | 0,000 | 0,000 | |
| F37 | 0,000 | | | 0,000 | | | 0,003 | | 0,000 | | 0,001 | | 0,000 | | 0,000 | | 0,000 | 0,001 | |
| F38 | 0,000 | | | 0,001 | | | 0,000 | | 0,000 | | 0,000 | | 0,000 | | 0,004 | | 0,003 | 0,004 | |
| F39 | 0,000 | | | 0,000 | | | 0,002 | | 0,000 | | 0,000 | | 0,000 | | 0,000 | | 0,000 | 0,000 | |
| F40 | 0,000 | | | 0,000 | | | 0,000 | | 0,000 | | 0,000 | | 0,000 | | 0,000 | | 0,000 | 0,000 | |

|  | **SPI** | **EEig07x** | **GGI1** | **GATS1v** | **GATS1p** | **GATS1m** | **SaaO** | **BELe1** | **IC4** |
| --- | --- | --- | --- | --- | --- | --- | --- | --- | --- |
| F1 | 0,680 | 0,588 | 0,621 | 0,009 | 0,000 | 0,000 | 0,000 | 0,134 | 0,519 |
| F2 | 0,004 | 0,189 | 0,026 | 0,111 | 0,047 | 0,011 | 0,012 | 0,059 | 0,107 |
| F3 | 0,015 | 0,046 | 0,011 | 0,569 | 0,595 | 0,458 | 0,001 | 0,087 | 0,035 |
| F4 | 0,156 | 0,009 | 0,135 | 0,072 | 0,002 | 0,000 | 0,046 | 0,004 | 0,043 |
| F5 | 0,001 | 0,001 | 0,005 | 0,007 | 0,015 | 0,077 | 0,020 | 0,051 | 0,007 |
| F6 | 0,004 | 0,000 | 0,002 | 0,001 | 0,004 | 0,000 | 0,164 | 0,006 | 0,017 |
| F7 | 0,007 | 0,011 | 0,000 | 0,005 | 0,086 | 0,004 | 0,000 | 0,085 | 0,009 |
| F8 | 0,002 | 0,000 | 0,000 | 0,005 | 0,023 | 0,027 | 0,137 | 0,002 | 0,005 |
| F9 | 0,003 | 0,003 | 0,000 | 0,001 | 0,002 | 0,018 | 0,047 | 0,001 | 0,003 |
| F10 | 0,002 | 0,012 | 0,006 | 0,000 | 0,037 | 0,023 | 0,167 | 0,342 | 0,015 |
| F11 | 0,006 | 0,001 | 0,018 | 0,007 | 0,058 | 0,185 | 0,014 | 0,000 | 0,012 |
| F12 | 0,002 | 0,012 | 0,017 | 0,005 | 0,050 | 0,039 | 0,116 | 0,001 | 0,006 |
| F13 | 0,000 | 0,002 | 0,024 | 0,017 | 0,001 | 0,016 | 0,010 | 0,013 | 0,002 |
| F14 | 0,009 | 0,002 | 0,006 | 0,000 | 0,005 | 0,015 | 0,183 | 0,004 | 0,005 |
| F15 | 0,018 | 0,001 | 0,000 | 0,010 | 0,004 | 0,044 | 0,021 | 0,013 | 0,007 |
| F16 | 0,000 | 0,012 | 0,000 | 0,009 | 0,001 | 0,000 | 0,023 | 0,017 | 0,000 |
| F17 | 0,007 | 0,004 | 0,005 | 0,001 | 0,008 | 0,007 | 0,000 | 0,028 | 0,002 |
| F18 | 0,003 | 0,000 | 0,003 | 0,055 | 0,009 | 0,000 | 0,012 | 0,026 | 0,000 |
| F19 | 0,001 | 0,000 | 0,009 | 0,005 | 0,004 | 0,004 | 0,008 | 0,044 | 0,005 |
| F20 | 0,009 | 0,008 | 0,006 | 0,002 | 0,001 | 0,000 | 0,000 | 0,000 | 0,002 |
| F21 | 0,001 | 0,001 | 0,002 | 0,003 | 0,003 | 0,003 | 0,001 | 0,014 | 0,022 |
| F22 | 0,002 | 0,003 | 0,000 | 0,002 | 0,000 | 0,000 | 0,002 | 0,005 | 0,120 |
| F23 | 0,001 | 0,004 | 0,018 | 0,011 | 0,000 | 0,010 | 0,002 | 0,043 | 0,003 |
| F24 | 0,004 | 0,007 | 0,001 | 0,046 | 0,000 | 0,017 | 0,002 | 0,001 | 0,000 |
| F25 | 0,007 | 0,001 | 0,000 | 0,002 | 0,004 | 0,000 | 0,003 | 0,015 | 0,012 |
| F26 | 0,009 | 0,005 | 0,025 | 0,019 | 0,002 | 0,008 | 0,001 | 0,000 | 0,000 |
| F27 | 0,000 | 0,000 | 0,000 | 0,013 | 0,001 | 0,006 | 0,001 | 0,001 | 0,031 |
| F28 | 0,002 | 0,003 | 0,026 | 0,000 | 0,002 | 0,008 | 0,003 | 0,001 | 0,000 |
| F29 | 0,003 | 0,001 | 0,009 | 0,000 | 0,000 | 0,000 | 0,000 | 0,002 | 0,006 |
| F30 | 0,003 | 0,059 | 0,000 | 0,000 | 0,000 | 0,000 | 0,000 | 0,000 | 0,004 |
| F31 | 0,002 | 0,002 | 0,000 | 0,001 | 0,019 | 0,015 | 0,001 | 0,000 | 0,000 |
| F32 | 0,016 | 0,002 | 0,016 | 0,003 | 0,000 | 0,000 | 0,000 | 0,000 | 0,001 |
| F33 | 0,002 | 0,009 | 0,000 | 0,003 | 0,003 | 0,000 | 0,000 | 0,001 | 0,000 |
| F34 | 0,005 | 0,000 | 0,001 | 0,000 | 0,003 | 0,002 | 0,000 | 0,000 | 0,001 |
| F35 | 0,002 | 0,000 | 0,001 | 0,004 | 0,010 | 0,002 | 0,001 | 0,000 | 0,000 |
| F36 | 0,014 | 0,001 | 0,006 | 0,002 | 0,002 | 0,000 | 0,000 | 0,000 | 0,000 |
| F37 | 0,000 | 0,000 | 0,000 | 0,000 | 0,000 | 0,000 | 0,000 | 0,000 | 0,000 |
| F38 | 0,000 | 0,000 | 0,000 | 0,000 | 0,000 | 0,000 | 0,000 | 0,000 | 0,000 |
| F39 | 0,000 | 0,000 | 0,000 | 0,001 | 0,001 | 0,000 | 0,000 | 0,000 | 0,000 |
| F40 | 0,000 | 0,000 | 0,000 | 0,000 | 0,000 | 0,000 | 0,000 | 0,000 | 0,000 |

|  | **TIE** | **VEA2** | **GVWAI-80** | **TPSA** | **PEOE_VSA_FPOS** | **S(-CH2-)** | **S(-C=)** | **S(=N-)** |
| --- | --- | --- | --- | --- | --- | --- | --- | --- |
| F1 | 0,741 | 0,659 | 0,166 | 0,456 | 0,003 | 0,005 | 0,015 | 0,005 |
| F2 | 0,002 | 0,199 | 0,035 | 0,185 | 0,115 | 0,099 | 0,278 | 0,024 |
| F3 | 0,015 | 0,010 | 0,021 | 0,088 | 0,119 | 0,011 | 0,110 | 0,000 |
| F4 | 0,145 | 0,007 | 0,010 | 0,027 | 0,061 | 0,012 | 0,179 | 0,226 |
| F5 | 0,002 | 0,001 | 0,000 | 0,096 | 0,151 | 0,150 | 0,006 | 0,283 |
| F6 | 0,001 | 0,015 | 0,002 | 0,001 | 0,007 | 0,354 | 0,000 | 0,010 |
| F7 | 0,002 | 0,001 | 0,043 | 0,000 | 0,001 | 0,018 | 0,078 | 0,080 |
| F8 | 0,001 | 0,000 | 0,034 | 0,050 | 0,109 | 0,000 | 0,002 | 0,000 |
| F9 | 0,000 | 0,002 | 0,004 | 0,006 | 0,009 | 0,002 | 0,055 | 0,170 |
| F10 | 0,012 | 0,004 | 0,002 | 0,000 | 0,005 | 0,064 | 0,072 | 0,012 |
| F11 | 0,003 | 0,003 | 0,024 | 0,002 | 0,022 | 0,000 | 0,013 | 0,064 |
| F12 | 0,000 | 0,011 | 0,379 | 0,000 | 0,003 | 0,020 | 0,005 | 0,000 |
| F13 | 0,000 | 0,000 | 0,151 | 0,001 | 0,001 | 0,032 | 0,029 | 0,003 |
| F14 | 0,000 | 0,001 | 0,011 | 0,000 | 0,001 | 0,007 | 0,010 | 0,000 |
| F15 | 0,007 | 0,002 | 0,028 | 0,012 | 0,008 | 0,010 | 0,047 | 0,002 |
| F16 | 0,001 | 0,001 | 0,044 | 0,003 | 0,044 | 0,000 | 0,002 | 0,010 |
| F17 | 0,001 | 0,000 | 0,000 | 0,002 | 0,278 | 0,016 | 0,002 | 0,021 |
| F18 | 0,005 | 0,001 | 0,004 | 0,008 | 0,007 | 0,121 | 0,004 | 0,007 |
| F19 | 0,006 | 0,000 | 0,002 | 0,002 | 0,032 | 0,000 | 0,004 | 0,018 |
| F20 | 0,004 | 0,001 | 0,004 | 0,003 | 0,008 | 0,048 | 0,003 | 0,001 |
| F21 | 0,000 | 0,001 | 0,000 | 0,000 | 0,004 | 0,006 | 0,000 | 0,000 |
| F22 | 0,001 | 0,002 | 0,000 | 0,003 | 0,001 | 0,001 | 0,003 | 0,013 |
| F23 | 0,000 | 0,003 | 0,000 | 0,001 | 0,002 | 0,003 | 0,027 | 0,009 |
| F24 | 0,001 | 0,001 | 0,000 | 0,003 | 0,005 | 0,009 | 0,003 | 0,003 |
| F25 | 0,000 | 0,000 | 0,001 | 0,004 | 0,001 | 0,000 | 0,007 | 0,010 |
| F26 | 0,004 | 0,005 | 0,026 | 0,001 | 0,000 | 0,004 | 0,003 | 0,004 |
| F27 | 0,000 | 0,002 | 0,003 | 0,004 | 0,000 | 0,001 | 0,022 | 0,004 |
| F28 | 0,002 | 0,001 | 0,003 | 0,004 | 0,000 | 0,000 | 0,003 | 0,000 |
| F29 | 0,000 | 0,009 | 0,000 | 0,001 | 0,000 | 0,002 | 0,006 | 0,007 |
| F30 | 0,000 | 0,002 | 0,001 | 0,000 | 0,000 | 0,000 | 0,002 | 0,000 |
| F31 | 0,000 | 0,008 | 0,000 | 0,001 | 0,001 | 0,000 | 0,000 | 0,004 |
| F32 | 0,008 | 0,009 | 0,000 | 0,004 | 0,000 | 0,002 | 0,006 | 0,000 |
| F33 | 0,003 | 0,029 | 0,000 | 0,000 | 0,000 | 0,000 | 0,000 | 0,003 |
| F34 | 0,014 | 0,009 | 0,000 | 0,004 | 0,000 | 0,001 | 0,001 | 0,001 |
| F35 | 0,003 | 0,001 | 0,000 | 0,009 | 0,000 | 0,001 | 0,000 | 0,001 |
| F36 | 0,001 | 0,000 | 0,000 | 0,013 | 0,000 | 0,000 | 0,001 | 0,001 |
| F37 | 0,007 | 0,002 | 0,000 | 0,003 | 0,000 | 0,000 | 0,001 | 0,000 |
| F38 | 0,000 | 0,000 | 0,000 | 0,001 | 0,000 | 0,000 | 0,000 | 0,000 |
| F39 | 0,005 | 0,000 | 0,000 | 0,002 | 0,000 | 0,000 | 0,000 | 0,001 |
| F40 | 0,000 | 0,000 | 0,000 | 0,000 | 0,000 | 0,000 | 0,000 | 0,000 |

|  | **S(-S-)** | **S(-O-)** | **S(>C<)** | **S(>CH-)** | **S(>N-)** |
| --- | --- | --- | --- | --- | --- |
| F1 | 0,003 | 0,084 | 0,045 | 0,120 | 0,000 |
| F2 | 0,016 | 0,041 | 0,003 | 0,110 | 0,240 |
| F3 | 0,044 | 0,125 | 0,006 | 0,008 | 0,034 |
| F4 | 0,003 | 0,051 | 0,230 | 0,014 | 0,002 |
| F5 | 0,169 | 0,042 | 0,004 | 0,001 | 0,055 |
| F6 | 0,001 | 0,381 | 0,016 | 0,072 | 0,319 |
| F7 | 0,326 | 0,000 | 0,010 | 0,001 | 0,012 |
| F8 | 0,083 | 0,100 | 0,022 | 0,028 | 0,002 |
| F9 | 0,050 | 0,010 | 0,045 | 0,014 | 0,106 |
| F10 | 0,010 | 0,012 | 0,042 | 0,001 | 0,009 |
| F11 | 0,073 | 0,002 | 0,175 | 0,005 | 0,000 |
| F12 | 0,007 | 0,000 | 0,002 | 0,068 | 0,001 |
| F13 | 0,005 | 0,003 | 0,049 | 0,373 | 0,051 |
| F14 | 0,049 | 0,064 | 0,071 | 0,012 | 0,000 |
| F15 | 0,016 | 0,000 | 0,231 | 0,062 | 0,000 |
| F16 | 0,007 | 0,001 | 0,001 | 0,009 | 0,076 |
| F17 | 0,070 | 0,002 | 0,005 | 0,002 | 0,000 |
| F18 | 0,011 | 0,011 | 0,021 | 0,011 | 0,002 |
| F19 | 0,004 | 0,021 | 0,007 | 0,000 | 0,000 |
| F20 | 0,004 | 0,001 | 0,001 | 0,078 | 0,024 |
| F21 | 0,002 | 0,003 | 0,000 | 0,000 | 0,006 |
| F22 | 0,002 | 0,002 | 0,003 | 0,003 | 0,003 |
| F23 | 0,014 | 0,001 | 0,000 | 0,002 | 0,019 |
| F24 | 0,005 | 0,016 | 0,001 | 0,000 | 0,011 |
| F25 | 0,004 | 0,000 | 0,003 | 0,000 | 0,010 |
| F26 | 0,001 | 0,000 | 0,002 | 0,002 | 0,000 |
| F27 | 0,006 | 0,002 | 0,001 | 0,000 | 0,002 |
| F28 | 0,007 | 0,000 | 0,000 | 0,000 | 0,002 |
| F29 | 0,000 | 0,000 | 0,001 | 0,001 | 0,000 |
| F30 | 0,000 | 0,001 | 0,000 | 0,000 | 0,002 |
| F31 | 0,002 | 0,011 | 0,001 | 0,000 | 0,001 |
| F32 | 0,000 | 0,005 | 0,001 | 0,000 | 0,005 |
| F33 | 0,000 | 0,000 | 0,001 | 0,000 | 0,004 |
| F34 | 0,001 | 0,001 | 0,000 | 0,001 | 0,000 |
| F35 | 0,001 | 0,002 | 0,000 | 0,000 | 0,000 |
| F36 | 0,000 | 0,003 | 0,000 | 0,000 | 0,000 |
| F37 | 0,000 | 0,000 | 0,000 | 0,000 | 0,000 |
| F38 | 0,000 | 0,001 | 0,000 | 0,000 | 0,000 |
| F39 | 0,000 | 0,002 | 0,000 | 0,000 | 0,001 |
| F40 | 0,000 | 0,000 | 0,000 | 0,000 | 0,000 |


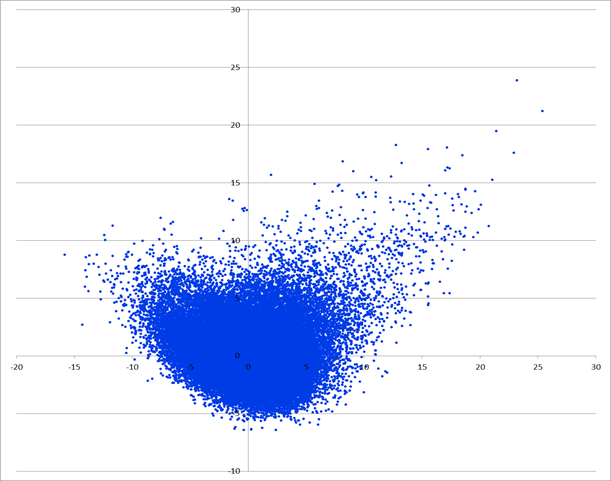


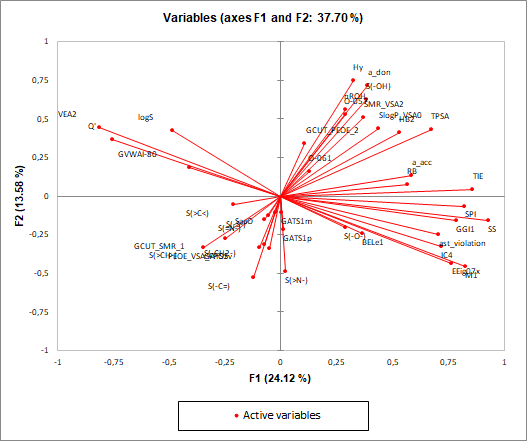

Supplement: Supplementary file 12 [file Table_7.docx]
